# Supplementary material for: Protocol for the Psychosis Immune Mechanism Stratified Medicine (PIMS) trial: a randomised double-blind placebo-controlled trial of single-dose tocilizumab in patients with psychosis
Source: BMJ Open. 2023 Mar 24;13(3):e067944. doi: 10.1136/bmjopen-2022-067944 (PMC10040013; doi:10.1136/bmjopen-2022-067944)
Supplement: Supplementary data [file bmjopen-2022-067944supp001.pdf]

Supplementary Material

Foley et al. Protocol for the Psychosis Immune Mechanism Stratified Medicine (PIMS) trial: A randomised double-blind placebo-controlled trial of single dose tocilizumab in patients with psychosis.

Table of Contents

eTable1: SPIRIT 2013 Checklist 2

Appendix I – Consent Form for Screening: All Participants 8

Appendix II – Informed Consent Form for Study Participation: Patients 10

Appendix III – Informed Consent Form for Study Participation: Healthy Controls 13

Foley ÉM, *et al. BMJ Open* 2023; 13:e067944. doi: 10.1136/bmjopen-2022-067944

eTable1: SPIRIT 2013 Checklist – Recommended items to address in a clinical trial protocol and related documents*

SPIRIT
STANDARD PROTOCOL ITEMS: RECOMMENDATIONS FOR INTERVENTIONAL TRIALS

Section/item	Item No	Description	Addressed on page number
**Administrative information**			
Title	1	Descriptive title identifying the study design, population, interventions, and, if applicable, trial acronym	1
Trial registration	2a	Trial identifier and registry name. If not yet registered, name of intended registry	4
	2b	All items from the World Health Organization Trial Registration Data Set	4 (ISRCTN 23256704)
Protocol version	3	Date and version identifier	-
Funding	4	Sources and types of financial, material, and other support	24 - 25
Roles and responsibilities	5a	Names, affiliations, and roles of protocol contributors	1, 24
	5b	Name and contact information for the trial sponsor	23
	5c	Role of study sponsor and funders, if any, in study design; collection, management, analysis, and interpretation of data; writing of the report; and the decision to submit the report for publication, including whether they will have ultimate authority over any of these activities	23-25

	5d	Composition, roles, and responsibilities of the coordinating centre, steering committee, endpoint adjudication committee, data management team, and other individuals or groups overseeing the trial, if applicable (see Item 21a for data monitoring committee)	23 - 25
**Introduction**			
Background and rationale	6a	Description of research question and justification for undertaking the trial, including summary of relevant studies (published and unpublished) examining benefits and harms for each intervention	6-9
	6b	Explanation for choice of comparators	6-9
Objectives	7	Specific objectives or hypotheses	9-10
Trial design	8	Description of trial design including type of trial (eg, parallel group, crossover, factorial, single group), allocation ratio, and framework (eg, superiority, equivalence, noninferiority, exploratory)	9
**Methods: Participants, interventions, and outcomes**			
Study setting	9	Description of study settings (e.g., community clinic, academic hospital) and list of countries where data will be collected. Reference to where list of study sites can be obtained	9, 11
Eligibility criteria	10	Inclusion and exclusion criteria for participants. If applicable, eligibility criteria for study centres and individuals who will perform the interventions (eg, surgeons, psychotherapists)	12, Table 1
Interventions	11a	Interventions for each group with sufficient detail to allow replication, including how and when they will be administered	11, 19, 21-22, Figure 1
	11b	Criteria for discontinuing or modifying allocated interventions for a given trial participant (eg, drug dose change in response to harms, participant request, or improving/worsening disease)	22
	11c	Strategies to improve adherence to intervention protocols, and any procedures for monitoring adherence (eg, drug tablet return, laboratory tests)	-
	11d	Relevant concomitant care and interventions that are permitted or prohibited during the trial	12, Table 1

Outcomes	12	Primary, secondary, and other outcomes, including the specific measurement variable (eg, systolic blood pressure), analysis metric (eg, change from baseline, final value, time to event), method of aggregation (eg, median, proportion), and time point for each outcome. Explanation of the clinical relevance of chosen efficacy and harm outcomes is strongly recommended	9-10, Figure 1, Table 2
Participant timeline	13	Time schedule of enrolment, interventions (including any run-ins and washouts), assessments, and visits for participants. A schematic diagram is highly recommended (see Figure)	18-19, Figure 1
Sample size	14	Estimated number of participants needed to achieve study objectives and how it was determined, including clinical and statistical assumptions supporting any sample size calculations	17
Recruitment	15	Strategies for achieving adequate participant enrolment to reach target sample size	18
**Methods: Assignment of interventions (for controlled trials)**			
Allocation:			
Sequence generation	16a	Method of generating the allocation sequence (eg, computer-generated random numbers), and list of any factors for stratification. To reduce predictability of a random sequence, details of any planned restriction (eg, blocking) should be provided in a separate document that is unavailable to those who enrol participants or assign interventions	17
Allocation concealment mechanism	16b	Mechanism of implementing the allocation sequence (eg, central telephone; sequentially numbered, opaque, sealed envelopes), describing any steps to conceal the sequence until interventions are assigned	17
Implementation	16c	Who will generate the allocation sequence, who will enrol participants, and who will assign participants to interventions	17
Blinding (masking)	17a	Who will be blinded after assignment to interventions (eg, trial participants, care providers, outcome assessors, data analysts), and how	17
	17b	If blinded, circumstances under which unblinding is permissible, and procedure for revealing a participant’s allocated intervention during the trial	22

**Methods: Data collection, management, and analysis**			
Data collection methods	18a	Plans for assessment and collection of outcome, baseline, and other trial data, including any related processes to promote data quality (eg, duplicate measurements, training of assessors) and a description of study instruments (eg, questionnaires, laboratory tests) along with their reliability and validity, if known. Reference to where data collection forms can be found, if not in the protocol	10-15, 18-19, Table 2
	18b	Plans to promote participant retention and complete follow-up, including list of any outcome data to be collected for participants who discontinue or deviate from intervention protocols	17-18
Data management	19	Plans for data entry, coding, security, and storage, including any related processes to promote data quality (eg, double data entry; range checks for data values). Reference to where details of data management procedures can be found, if not in the protocol	23-24
Statistical methods	20a	Statistical methods for analysing primary and secondary outcomes. Reference to where other details of the statistical analysis plan can be found, if not in the protocol	17-18
	20b	Methods for any additional analyses (eg, subgroup and adjusted analyses)	17-18
	20c	Definition of analysis population relating to protocol non-adherence (eg, as randomised analysis), and any statistical methods to handle missing data (eg, multiple imputation)	-
**Methods: Monitoring**			
Data monitoring	21a	Composition of data monitoring committee (DMC); summary of its role and reporting structure; statement of whether it is independent from the sponsor and competing interests; and reference to where further details about its charter can be found, if not in the protocol. Alternatively, an explanation of why a DMC is not needed	23
	21b	Description of any interim analyses and stopping guidelines, including who will have access to these interim results and make the final decision to terminate the trial	-
Harms	22	Plans for collecting, assessing, reporting, and managing solicited and spontaneously reported adverse events and other unintended effects of trial interventions or trial conduct	20-22

Foley ÉM, *et al. BMJ Open* 2023; 13:e067944. doi: 10.1136/bmjopen-2022-067944

Auditing	23	Frequency and procedures for auditing trial conduct, if any, and whether the process will be independent from investigators and the sponsor	-
**Ethics and dissemination**			
Research ethics approval	24	Plans for seeking research ethics committee/institutional review board (REC/IRB) approval	22
Protocol amendments	25	Plans for communicating important protocol modifications (eg, changes to eligibility criteria, outcomes, analyses) to relevant parties (eg, investigators, REC/IRBs, trial participants, trial registries, journals, regulators)	22
Consent or assent	26a	Who will obtain informed consent or assent from potential trial participants or authorised surrogates, and how (see Item 32)	22-23
	26b	Additional consent provisions for collection and use of participant data and biological specimens in ancillary studies, if applicable	22-23
Confidentiality	27	How personal information about potential and enrolled participants will be collected, shared, and maintained in order to protect confidentiality before, during, and after the trial	23-24
Declaration of interests	28	Financial and other competing interests for principal investigators for the overall trial and each study site	25
Access to data	29	Statement of who will have access to the final trial dataset, and disclosure of contractual agreements that limit such access for investigators	23
Ancillary and post-trial care	30	Provisions, if any, for ancillary and post-trial care, and for compensation to those who suffer harm from trial participation	22
Dissemination policy	31a	Plans for investigators and sponsor to communicate trial results to participants, healthcare professionals, the public, and other relevant groups (eg, via publication, reporting in results databases, or other data sharing arrangements), including any publication restrictions	24
	31b	Authorship eligibility guidelines and any intended use of professional writers	24
	31c	Plans, if any, for granting public access to the full protocol, participant-level dataset, and statistical code	-

Foley ÉM, *et al. BMJ Open* 2023; 13:e067944. doi: 10.1136/bmjopen-2022-067944

**Appendices**			
Informed consent materials	32	Model consent form and other related documentation given to participants and authorised surrogates	Appendix I, II and III
Biological specimens	33	Plans for collection, laboratory evaluation, and storage of biological specimens for genetic or molecular analysis in the current trial and for future use in ancillary studies, if applicable	23-24

*It is strongly recommended that this checklist be read in conjunction with the SPIRIT 2013 Explanation & Elaboration for important clarification on the items. Amendments to the protocol should be tracked and dated. The SPIRIT checklist is copyrighted by the SPIRIT Group under the Creative Commons "Attribution-NonCommercial-NoDerivs 3.0 Unported" license.

Appendix I – Consent Form for Screening: All Participants

Informed Consent Form for Screening

REC No. 22/EE/0010, Date: 08.02.2022, Version: 1.2

Psychosis Immune Mechanism Stratified Medicine Trial: The PIMS Trial

PARTICIPANT ID: ________

Thank you for considering taking part in the PIMS Trial. The research team must explain the eligibility assessment of the study to you before you agree to take part. If you have any questions arising from the Participant Information Sheet or from the explanation already given to you, please ask a member of the research team before you decide to participate. You will be given a copy of this Informed Consent Form to keep for future reference.

Please read the statements below and insert your initial in the box next to each statement if you agree with them:

**Statement**	**Initial Here**	
1. I have read and understood the information sheet version XX, DD.MM.YYYY and have had the opportunity to ask questions.		
2. I understand that my participation is voluntary and I am free to withdraw at any time without giving a reason, without my current or future medical care or legal rights being affected.		
3. I agree to provide blood samples for eligibility screening. I understand my blood samples will be analysed to test for evidence of immune activation. The purposes and possible risks of donating these samples have been explained to me. I understand that donated samples will be considered a gift but I will have the right to withdraw permission for analysis.		
4. I agree that my GP can be told that I am participating in the eligibility assessment of the PIMS Trial, and can be informed if any unexpected results are found pertaining specifically to my health.		
5. I consent for my GP/Psychiatrist to share information from my medical record in order to confirm my eligibility to take part in this study. The study team may access my GP/Psychiatrist records if necessary.		
Optional (Not agreeing to these will not exclude you from this study). Please tick Yes / No (as appropriate)	Yes	No
6. I agree that the samples can be stored after completion of the screening analysis, for use in future, ethically approved, non-genetic studies, even if I am deemed non-eligible to partake in the PIMS study.		
7. I agree that data and samples can be stored after completion of the PIMS Trial for use in future, ethically approved, genetic studies. This includes the main stocks of any genetic material collected, such as DNA and RNA.		

If you want to participate in the screening session of the PIMS Trial, please sign your name below:

Participant Signature ______________________

Participant Full Name _____________________

8

Date ____/ ____/ _______

The researcher who has explained this study to you also needs to sign this form:

Staff Signature ______________________

Staff Full Name _____________________

Date ____/ ____/ _______

Thank you for your help.

By completing and returning this form, you are giving us your consent that the personal information you provide will be treated as strictly confidential and handled in accordance with the provisions of the UK Data Protection Act 2018.

**When completed: 1 for participant; and 1 for researcher site file.*

Foley ÉM, *et al. BMJ Open* 2023; 13:e067944. doi: 10.1136/bmjopen-2022-067944

Appendix II – Informed Consent Form for Study Participation: Patients

Informed Consent Form for Study Participation

REC No. 22/EE/0010, Date: 24.08.2022, Version: 1.5

Psychosis Immune Mechanism Stratified Medicine Trial: The PIMS Trial

PARTICIPANT ID: ________

Thank you for taking part in the PIMS Trial eligibility assessment. Based on this assessment, you are eligible to take part in the PIMS Trial. Before you agree to take part, the research team must explain the study to you. If you have any questions arising from the Participant Information Sheet or from the explanation already given to you, please ask a member of the research team before you decide to participate. You will be given a copy of this Informed Consent Form to keep for future reference.

Please read the statements below and insert your initial in the box next to each statement if you agree with them:

Statement	Initial Here
1. I have read and understood the information sheet version XX dated DD.MM.YYYY and have had the opportunity to ask questions.	
2. I agree to take part in the PIMS Trial. I understand that my participation is voluntary and I am free to withdraw at any time without giving a reason, without my current or future medical care or legal rights being affected.	
3. I understand that confidentiality and anonymity will be maintained and it will not be possible to identify me in any publications.	
4. I agree to partake in interviews, complete questionnaires, and cognitive tests as part of this study. I understand what will happen during the study assessments.	
5. I agree to provide blood samples. The purposes and possible risks of donating these samples have been explained to me. I understand that donated samples will be considered a gift but I will have the right to withdraw permission for analysis.	
6. I understand that blood samples collected from me will be used to measure non-genetic factors such as biochemical changes in the blood.	
7. I agree that the samples and information I provide can be stored, used and shared between PIMS Trial sites and with collaborators/contractors for the purpose of the study.	
8. I understand that blood samples collected will be stored at PIMS Trial centres.	
9. I understand that any of my samples (labelled with an anonymous ID only), or any information obtained from them, including the sequence of my genetic material, may be sent to specialist research laboratories in the UK and abroad for analyses and the results returned to PIMS Trial centres. Researchers at these laboratories have no access to personal information about study participants.	
10. I agree, if necessary, to provide blood/urine samples to test for pregnancy, COVID-19 immunity, Hepatitis B, Hepatitis C, HIV, VZV and Tuberculosis, and to undergo a chest X ray.	
11. I agree to being randomised into the tocilizumab or placebo group if deemed eligible to take part.	

Foley ÉM, *et al. BMJ Open* 2023; 13:e067944. doi: 10.1136/bmjopen-2022-067944

12. I understand that taking part will involve the administration of a single intravenous infusion of the anti-inflammatory drug tocilizumab or normal saline.		
13. I agree that my GP can be told that I am participating in this study, and about any findings that require further attention.		
14. I understand that information related to my participation in this study may be accessed by responsible individuals from the sponsors of this study for quality control purposes. I give permission for these individuals to have access to this data.		
15. I understand that relevant sections of my medical notes and data collected during the study, may be looked at by individuals from research team, from regulatory authorities or from the NHS Trust, where it is relevant to my taking part in this research. I give permission for these individuals to have access to my records.		
Optional (Not agreeing to these will not exclude you from this study). Please tick Yes / No (as appropriate)	Yes	No
16. I agree to undergo brain scans as part of the PIMS Trial.		
17. I agree to be contacted in future by researchers to participate in follow up studies to this project, or in future studies of a similar nature.		
18. I understand that researchers may use the blood samples for genetic analysis.		
19. I agree that the samples can be stored after completion of the PIMS Trial for use in future, ethically approved, non-genetic studies.		
20. I agree that the information I give can be stored after completion of the PIMS Trial for use in future, ethically approved, non-genetic studies.		
21. I agree that data and samples can be stored after completion of the PIMS Trial for use in future, ethically approved, genetic studies. This includes the main stocks of any genetic material collected, such as DNA and RNA.		

If you want to participate in the PIMS Trial, please sign your name below:

Participant Signature ______________________

Participant Full Name _____________________

Date ____/ ____/ _______

Research staff who has explained this study to you also needs to sign this form:

Staff Signature ______________________

Staff Full Name _____________________

Date ____/ ____/ _______

Foley ÉM, *et al. BMJ Open* 2023; 13:e067944. doi: 10.1136/bmjopen-2022-067944

Thank you for your help.

By completing and returning this form, you are giving us your consent that the personal information you provide will be treated as strictly confidential and handled in accordance with the provisions of the UK Data Protection Act 2018.

**When completed: 1 for participant; and 1 for researcher site file.*

Appendix III – Informed Consent Form for Study Participation: Healthy Controls

Healthy Controls Informed Consent Form for Study Participation

REC No. 22/EE/0010, Date: 17.02.2022, Version: 1

Psychosis Immune Mechanism Stratified Medicine Trial: The PIMS Trial

PARTICIPANT ID: ________

Thank you for taking part in the PIMS Trial eligibility assessment. Based on this assessment, you are eligible to take part in the PIMS Trial. Before you agree to take part, the research team must explain the study to you. If you have any questions arising from the Participant Information Sheet or from the explanation already given to you, please ask a member of the research team before you decide to participate. You will be given a copy of this Informed Consent Form to keep for future reference.

Please read the statements below and insert your initial in the box next to each statement if you agree with them:

**Statement**	**Initial Here**	
1. I have read and understood the information sheet version XX dated DD.MM.YYYY and have had the opportunity to ask questions.		
2. I agree to take part in the PIMS Trial. I understand that my participation is voluntary, and I am free to withdraw at any time without giving a reason, without my current or future medical care or legal rights being affected.		
3. I understand that confidentiality and anonymity will be maintained, and it will not be possible to identify me in any publications.		
4. I agree to partake in interviews, complete questionnaires, and cognitive tests as part of this study. I understand what will happen during the study assessments.		
5. I agree to provide blood samples. The purposes and possible risks of donating these samples have been explained to me. I understand that donated samples will be considered a gift, but I will have the right to withdraw permission for analysis.		
6. I understand that my blood samples collected will be stored at PIMS Trial centres.		
7. I understand that blood samples collected from me will be used to measure non-genetic factors such as biochemical changes in the blood.		
8. I understand that any of my samples (labelled with an anonymous ID only), or any information obtained from them, including the sequence of my genetic material, may be sent to specialist research laboratories in the UK and abroad for analyses and the results returned to PIMS Trial centres. Researchers at these laboratories have no access to personal information about study participants.		
9. I agree that the samples and information I provide can be stored, used and shared between PIMS Trial sites and with collaborators/contractors for the purpose of the study.		
10. I understand that information related to my participation in this study may be accessed by responsible individuals from the sponsors of this study for quality control purposes. I give permission for these individuals to have access to this data.		
Optional (Not agreeing to these will not exclude you from this study). Please tick Yes / No (as appropriate)	Yes	No

11. I agree to undergo brain scans as part of the PIMS Trial.		
12. I agree to be contacted in future by researchers to participate in follow up studies to this project, or in future studies of a similar nature.		
13. I understand that researchers may use the blood samples for genetic analysis.		
14. I agree that the samples can be stored after completion of the PIMS Trial for use in future, ethically approved, non-genetic studies.		
15. I agree that the information I give can be stored after completion of the PIMS Trial for use in future, ethically approved, non-genetic studies.		
16. I agree that data and samples can be stored after completion of the PIMS Trial for use in future, ethically approved, genetic studies. This includes the main stocks of any genetic material collected, such as DNA and RNA.		

If you want to participate in the PIMS Trial, please sign your name below:

Participant Signature ______________________

Participant Full Name _____________________

Date ____/ ____/ _______

Research staff who has explained this study to you also needs to sign this form:

Staff Signature ______________________

Staff Full Name _____________________

Date ____/ ____/ _______

Thank you for your help.

By completing and returning this form, you are giving us your consent that the personal information you provide will be treated as strictly confidential and handled in accordance with the provisions of the UK Data Protection Act 2018.

**When completed: 1 for participant; and 1 for researcher site file.*

Foley ÉM, *et al. BMJ Open* 2023; 13:e067944. doi: 10.1136/bmjopen-2022-067944
