# Supplementary material for: Protocol for the Psychosis Immune Mechanism Stratified Medicine (PIMS) trial: a randomised double-blind placebo-controlled trial of single-dose tocilizumab in patients with psychosis
Source: BMJ Open. 2023 Mar 24;13(3):e067944. doi: 10.1136/bmjopen-2022-067944 (PMC10040013; doi:10.1136/bmjopen-2022-067944)
Supplement: Author's manuscript [file bmjopen-2022-067944.draft_revisions.pdf]

BMJ Open is committed to open peer review. As part of this commitment we make the peer review history of every article we publish publicly available.

When an article is published we post the peer reviewers' comments and the authors' responses online. We also post the versions of the paper that were used during peer review. These are the versions that the peer review comments apply to.

The versions of the paper that follow are the versions that were submitted during the peer review process. They are not the versions of record or the final published versions. They should not be cited or distributed as the published version of this manuscript.

BMJ Open is an open access journal and the full, final, typeset and author-corrected version of record of the manuscript is available on our site with no access controls, subscription charges or pay-per-view fees (http://bmjopen.bmj.com).

If you have any questions on BMJ Open's open peer review process please email info.bmjopen@bmj.com

BMJ Open

Protocol for the Psychosis Immune Mechanism Stratified Medicine (PIMS) trial: A randomised double-blind placebo-controlled trial of single dose tocilizumab in patients with psychosis

Journal:	*BMJ Open*
Manuscript ID	bmjopen-2022-067944
Article Type:	Protocol
Date Submitted by the Author:	02-Sep-2022

Complete List of Authors:	Foley, Éimear; University of Bristol, MRC Integrative Epidemiology Unit, Population Health Sciences; Bristol Medical School, Centre for Academic Mental Health, Population Health Sciences<br>Griffiths, Sian Lowri; University of Birmingham, Institute for Mental Health and Centre for Human Brain Health<br>Murray, Alexander; University of Birmingham, Institute for Mental Health and Centre for Human Brain Health<br>Rogers, Jack; University of Birmingham, Institute for Mental Health and Centre for Human Brain Health<br>Corsi-Zuelli, Fabiana; University of Birmingham, Institute for Mental Health and Centre for Human Brain Health; University of Sao Paulo, Department of Neuroscience and Behaviour<br>Hickinbotham, Hannah; University of Cambridge, Department of Psychiatry<br>Kaser, Muzaffer; University of Cambridge, Psychiatry; Cambridgeshire and Peterborough NHS Foundation Trust<br>Murray, Graham K.; University of Cambridge, Psychiatry; Cambridgeshire and Peterborough NHS Foundation Trust<br>Deakin, Bill; The University of Manchester, Faculty of Medicine, Biology and Health<br>Jadon, Deepak; University of Cambridge, Medicine<br>Suckling, John; University of Cambridge, Psychiatry; Cambridgeshire and Peterborough NHS Foundation Trust<br>Barnes, Nicholas ; University of Birmingham, Institute of Clinical Sciences<br>Upthegrove, Rachel; University of Birmingham, Institute for Mental Health and Centre for Human Brain Health; Early Intervention Service, Birmingham Women's and Children's NHS Foundation Trust<br>Khandaker, Golam M.; University of Bristol, MRC Integrative Epidemiology Unit, Population Health Sciences; NIHR Bristol Biomedical Research Centre

Keywords:	Schizophrenia & psychotic disorders < PSYCHIATRY, IMMUNOLOGY, Clinical trials < THERAPEUTICS, Magnetic resonance imaging < RADIOLOGY & IMAGING

SCHOLARONE™
Manuscripts

Protocol for the Psychosis Immune Mechanism Stratified Medicine (PIMS) trial: A randomised double-blind placebo-controlled trial of single dose tocilizumab in patients with psychosis

Éimear M. Foley[a,b]; Sian Lowri Griffiths[c]; Alexander Murray[c]; Jack Rogers[c]; Fabiana Corsi-Zuelli[c,d,e]; Hannah Hickinbotham[f]; Muzaffer Kaser[f,g]; Graham K. Murray[f,g]; Bill Deakin[h]; Deepak Jadon[i]; John Suckling[f,g]; Nicholas M. Barnes[e;] Rachel Upthegrove[c,j,†]; and Golam M. Khandaker[a,b,k,l,†,*]; for the PIMS Collaboration

[a] MRC Integrative Epidemiology Unit, Population Health Sciences, Bristol Medical School, University of Bristol, Bristol, United Kingdom

[b] Centre for Academic Mental Health, Population Health Sciences, Bristol Medical School, University of Bristol, Bristol, United Kingdom

[c] Institute for Mental Health and Centre for Human Brain Health, University of Birmingham, Birmingham, United Kingdom

[d] Department of Neuroscience and Behaviour, Division of Psychiatry, Ribeirão Preto Medical School, University of São Paulo, São Paulo, Brazil

[e] Institute of Clinical Sciences, College of Medical and Dental Sciences, University of Birmingham, United Kingdom

[f] Department of Psychiatry, University of Cambridge, United Kingdom

[g] Cambridgeshire and Peterborough NHS Foundation Trust, United Kingdom

[h] Faculty of Biology, Medicine and Health, Division of Neuroscience and Experimental Psychology, School of Biological Sciences, University of Manchester, Manchester Academic Health Science Centre, Manchester, United Kingdom

[i] Department of Medicine, University of Cambridge, United Kingdom

For peer review only

[j] Early Intervention Service, Birmingham Women's and Children's NHS Foundation Trust

[k] NIHR Bristol Biomedical Research Centre, Bristol, United Kingdom

[l] Avon and Wiltshire Mental Health Partnership NHS Trust, Bristol, United Kingdom

† Joint senior authors

* **Corresponding author and address:** Prof Golam M. Khandaker, MRC Integrative Epidemiology Unit, Oakfield House, Oakfield Grove, Clifton, Bristol, BS8 2BN, UK

Email: golam.khandaker@bristol.ac.uk

For peer review only

For peer review only - http://bmjopen.bmj.com/site/about/guidelines.xhtml

ABSTRACT

Introduction: Evidence suggests a potentially causal role of interleukin 6 (IL-6), a pleiotropic cytokine that generally promotes inflammation, in the pathogenesis of psychosis, but no interventional studies elucidating potential mechanisms in patients with psychosis, stratified using inflammatory markers, have been conducted. Tocilizumab is a humanised monoclonal antibody targeting the IL-6 receptor to inhibit IL-6 signalling licensed in the UK for treatment of rheumatoid arthritis. The primary objective of this study is to test whether IL-6 contributes to the pathogenesis of psychosis, and to examine potential mechanisms by which IL-6 affects psychotic symptoms. A secondary objective is to examine characteristics of inflammation-associated psychosis.

Methods and analysis: A proof-of-concept study employing a randomised, parallel-group, double-blind, placebo-controlled design testing the effect of IL-6 inhibition on anhedonia in patients with psychosis. Approximately 60 participants with diagnosis of schizophrenia and related psychotic disorders (ICD-10 codes F20, F22, F25, F28, F29) with evidence of low-grade inflammation (IL-6 ≥0.7pg/ml) will receive either one intravenous infusion of tocilizumab (4.0mg/kg; max 800mg) or normal saline. Psychiatric measures and blood samples will be collected at baseline, and 7-, 14-, and 28-days post-infusion. Cognitive and neuroimaging data will be collected at baseline and 14 days post-infusion. In addition, approximately 30 patients with psychosis without evidence of inflammation (IL-6 <0.7pg/ml) and 30 matched healthy controls will be recruited to complete identical baseline assessments to allow for comparison of the characteristic features of inflammation-associated psychosis.

Ethics and dissemination: The study is sponsored by the University of Bristol and has been approved by the Cambridge East Research Ethics Committee (reference: 22/EE/0010; IRAS project ID: 301682). Study findings will be published in peer-review journals. Findings will be also disseminated by scientific presentation and by other means.

For peer review only

For peer review only - http://bmjopen.bmj.com/site/about/guidelines.xhtml

Trial registration number: ISRCTN 23256704

KEYWORDS: Psychotic Disorders; Negative Symptoms; Interleukin 6; Immunotherapy; Tocilizumab; Clinical Trial.

For peer review only

For peer review only - http://bmjopen.bmj.com/site/about/guidelines.xhtml

ARTICLE SUMMARY

Strengths and limitations of this study

- Adopting a randomised controlled trial (RCT) design and patient selection based on elevated level of IL-6 (in addition to other criteria) will help examine the causal role of IL-6, and the therapeutic potential of targeting IL-6 pathway, in psychosis.
- The use of target specific intervention (anti-IL6R monoclonal antibody tocilizumab) will help assess the clinical relevance of IL-6 and related up- and downstream inflammatory cytokines in psychosis.
- The use of neuroimaging, cognitive tests, and extensive peripheral blood biomarker exploration before and after tocilizumab treatment to assess potential mechanisms of effect.
- One dose of tocilizumab is unlikely to be sufficient to test the efficacy of this drug as potential treatment for psychosis.
- Tocilizumab inhibits both anti-inflammatory (classic) and pro-inflammatory (trans) pathways of IL-6 that may have complementary or differential effects relevant to potential therapeutic effects.

For peer review only - http://bmjopen.bmj.com/site/about/guidelines.xhtml

Word count: 3,956

INTRODUCTION

Scientific background and study rationale

The neuroimmune hypothesis of schizophrenia proposes that mild peripheral immune activation gives rise to an inflammatory response in the brain and neurobiological changes associated with psychotic illness [1–4]. Meta-analytic evidence is clear that circulating concentrations of interleukin 6 (IL-6) and other inflammatory proteins, such as C-reactive protein (CRP), are increased in patients with psychosis, including treatment naïve first episode psychosis (FEP) [5], compared with controls [6–9]. Prospective cohort studies show that these indices of mild immune activation precede the onset of symptoms [10,11]. Furthermore, genetic variants known to increase IL-6 concentrations are associated with genetic risk of schizophrenia [12,13]. These Mendelian randomization studies eliminate the possibility that raised IL-6 concentrations are a consequence of environmental exposures associated with schizophrenia, such as obesity and smoking and instead suggest that IL-6 has a causal role in psychosis. Extending this approach using the UK Biobank population, we found that genetically-predicted levels of IL-6 were associated with reduced grey matter primarily in the middle temporal gyrus, a region whose gene expression profile is enriched for IL-6 pathway proteins and for neuropsychiatric disorder ontologies [14]. Moreover, clinical studies report correlations between IL-6 levels and structural brain changes in individuals with schizophrenia [15], with reduced grey matter volume being exaggerated in patients with psychosis and elevated inflammatory cytokines [16]. Though this causal evidence strongly implicates IL-6, only an intervention study in patients can test the causal hypothesis.

The neuroimmune hypothesis generally assumes that microglia, the brain's resident immune cells, are activated and pathogenic in schizophrenia. This is supported by traditional

For peer review only

For peer review only - http://bmjopen.bmj.com/site/about/guidelines.xhtml

neuropathological studies and initial in-vivo PET imaging studies [17–19], possibly reflecting impaired cellular control of inflammation or oxidative defence. Inflammatory damage may also account for evidence of oxidative stress from MRS glutathione studies [20]. However, whether microglia are the direct target of IL-6 is unclear and it is not certain that IL-6 can cross the blood-brain barrier and/or increase its permeability to circulating inflammatory cells, cytokines, and chemokines [2,21]. Additionally, it is increasingly uncertain whether microglial inflammation, as traditionally understood, occurs in schizophrenia [22,23]. Recent meta-analyses of PET radioligand binding studies report decreased rather than increased radioligand binding to activated microglia [24,25]. This may account for the unexpected lack of therapeutic benefit of the anti-microglial antibiotic, minocycline, in recent large clinical trials [26,27]. Furthermore, large transcriptomic studies in post-mortem brains report no change or reduction in microglial gene expression but increases in astrocytic expression [23,28–32]. It is increasingly understood that both peripheral immune responses and brain glial function are regulated by specialised T cells (Tregs), a subset of which reside in brain parenchyma [33,34]. A novel proposal is that Treg hypofunction accounts for mild peripheral immune disinhibition and dysregulated astroglial-microglial interaction, such that microglia are driven into a developmental, synapse-pruning phenotype while astroglia disrupt neurotransmitter function [33,34]. Importantly, there are bidirectional interactions between IL-6 and Treg function [34]. Crucially, we will measure IL-6 in addition to cellular and molecular markers of immune function and investigate how they correlate with central markers and clinical state.

Previous attempts testing the inflammatory hypothesis in therapeutic clinical trials have been attempted. However, little evidence of overall efficacy has been found [35]. These trials have generally tested broad spectrum agents, such as non-steroidal anti-inflammatory drugs, with no attempt to stratify patients according to evidence of inflammation. A trial

For peer review only - http://bmjopen.bmj.com/site/about/guidelines.xhtml

using tocilizumab, a humanised monoclonal antibody (mAb) against the IL-6 receptor currently licensed in the UK for treatment of rheumatoid arthritis (RA) and severe coronavirus disease, reported no improvements in any clinical measure in a small sample of 36 patients with established schizophrenia [36]. However, as mentioned previously, no stratification by inflammatory markers or any mechanistic immune measures was applied. Low-grade inflammation is associated with poor response to antipsychotic drugs [37], but immunotherapy is unlikely to be relevant for all patients with psychosis. Meta-analysis suggests that evidence of immune activation, defined by elevated CRP levels, is present in a quarter to one third of patients with schizophrenia [38]. A randomised controlled trial of infliximab, an anti-tumour necrosis factor alpha (TNF-α) mAb, reported that antidepressant response was associated with higher CRP levels at baseline [39], suggesting that patients with evidence of immune activation may be better candidates for immunotherapy trials. As far as we are aware, no previous clinical trial has selected patients with schizophrenia based on evidence of immune activation.

Selection of patients with particular symptom profiles and/or stage of illness may also be a useful strategy that needs to be employed in immunotherapy trials for schizophrenia. A wide variety of symptoms occur in schizophrenia such as hallucinations, delusions, anhedonia, cognitive dysfunction, and affective symptoms and presentation of these symptoms differ from one individual to another. Some symptoms may be more related to inflammation than others. For instance, a recent study from the ALSPAC birth cohort reported that out of 20 positive and negative symptoms, CRP is particularly associated with anhedonia and auditory hallucinations [40]. Anhedonia and amotivation are strongly associated with poor functional outcomes in depression and schizophrenia, and present a formidable barrier to returning to work or building relationships [41,42]. Patients with psychotic disorders also present with cognitive deficits in a range of domains [43]. Available

For peer review only - http://bmjopen.bmj.com/site/about/guidelines.xhtml

antipsychotic medications have a limited effects on poor cognitive functioning in psychosis [44]. Illness stage may also be of relevance. Meta-analytic data has revealed no differences in IL-6 levels between stable, medicated patients with schizophrenia and controls, although compared with controls, IL-6 levels were similarly elevated in patients with FEP and those with acute relapse [7]. A separate meta-analysis found evidence of elevated blood cytokine levels in acutely and chronically ill patients with schizophrenia [6]. Focusing on particular inflammation-related symptoms and/or illness stage may increase the chance of success for immunotherapy trials.

For peer review only

Proposed study

The proposed two-year study is a UK multi-site (Bristol, Birmingham, and Cambridge) proof-of-concept, randomised, parallel-group, double-blind, placebo-controlled trial.

Study aims and hypotheses

The primary aim of this trial is to examine potential mechanisms by which IL-6 affects anhedonia, psychotic symptoms, and cognition. Our primary hypothesis is that inhibition of IL-6 signalling with a single intravenous infusion of anti-IL6R monoclonal antibody, tocilizumab, in individuals with psychosis and elevated IL-6 at baseline will attenuate symptoms of anhedonia and amotivation in patients with psychosis, relative to placebo. This will provide further evidence for a potential causal role of inflammation in psychosis. Our secondary hypothesis is that reduction in peripheral inflammation after tocilizumab infusion in patients with psychosis and evidence of inflammation will be associated with central measures of oxidative stress and relevant resting state brain function.

We will also conduct deep immunophenotyping of peripheral blood mononuclear cell subsets ($CD4^+$, $CD8^+$, Tregs, natural killer and natural killer-T cells, monocytes, and B cells)

For peer review only - http://bmjopen.bmj.com/site/about/guidelines.xhtml

to characterise their absolute number, frequency, and function. Our primary mechanistic outcome is the level of IL-6/STAT3 signalling inhibition within both innate and adaptive immune cells using multi-colour flow cytometry with an established optimised pSTAT3 phosflow assay. This will help identify the potential cellular impact of peripheral inflammation in psychosis, which is largely unknown. Functional assessment of IL-6/STAT3 signalling in immune cell subsets and their response to exogenous IL-6 stimulation will inform abnormal immune response in psychosis and allow measurement of response to tocilizumab at the cellular level.

A secondary objective is to carry out an observational study to examine clinical and biomarker differences and similarities between patients with psychotic disorder with and without evidence of inflammation and healthy controls (HCs). We hypothesise that individuals with psychotic disorder and evidence of inflammation, compared to those without evidence of inflammation and HCs, will have increased symptoms of anhedonia and amotivation, poorer cognitive functioning, and cellular and brain-based measures of immune dysfunction.

METHODS

This protocol has been prepared in accordance with the Standard Protocol Items: Recommendations for Interventional Trials (SPIRIT) 2013 statement [45]. Please see supplementary eTable 1 for the SPIRIT checklist.

Patient and public involvement

The study protocol was prepared in collaboration with individuals with lived experience of mental illness who contributed to the development of participant information sheet, consent forms, and data collection procedures.

For peer review only - http://bmjopen.bmj.com/site/about/guidelines.xhtml

Study design and sample

See Figure 1 for an overview of study design. Individuals residing in Bristol, Birmingham, or Cambridge in the United Kingdom will be recruited. Approximately 60 participants with psychosis and evidence of inflammation (i.e., IL-6 ≥0.7pg/ml) will be randomised to receive either one intravenous infusion of tocilizumab (drug) or normal saline (placebo). For the secondary, observational study, we will compare baseline characteristics of the intervention cohort with approximately 30 participants with psychosis without evidence of inflammation (i.e., IL-6 <0.7pg/ml), and approximately 30 HCs. Participants without evidence of inflammation and controls will not be randomised as they will not receive any intervention. Neuroimaging will only be undertaken by those without MRI contraindications who have given specific informed consent for MRI. Participants not eligible or not consenting for MRI will take part in all other aspects of the study.

Intervention

Single intravenous infusion of tocilizumab (4.0mg/kg; max 800mg in total) or normal saline given to participants with psychosis and evidence of inflammation. Tocilizumab blocks both IL-6 classic and trans-signalling – the latter being responsible for most of the inflammatory effects of IL-6 – providing broad inhibition of IL-6 signalling and a strong test of a casual role for IL-6 in psychosis [46]. Tocilizumab is the first-in-class, humanized monoclonal antibody against the IL-6R, commercially available and licensed in the UK for treatment of RA. Approved dosage of tocilizumab for treatment of RA is 2, 4, or 8mg/kg; max 800mg in total. In RA, a single tocilizumab infusion has shown to improve clinical and laboratory measures within 48 hours, with most noticeable results in one-to-two weeks [47,48]. The follow-up schedule for our study is in keeping with this observation.

For peer review only

For peer review only - http://bmjopen.bmj.com/site/about/guidelines.xhtml

Eligibility criteria

We will recruit participants aged 18-40 years. Patient participants must meet International Classification of Diseases 10th Revision (ICD-10) criteria for a diagnosis of schizophrenia and related psychoses (ICD-10 code F20, F22, F25, F28, F29) at the time of eligibility assessment, be within three years of first diagnosis of psychotic disorder, be on a stable treatment regime with no recent (within two weeks) initiation, cessation, or change in class of antipsychotic medication, and have a Positive and Negative Syndrome Scale (PANSS) item score ≥3 on P1 (delusions), P2 (conceptual disorganisation), P3 (hallucinatory behaviour), or P6 (suspiciousness/persecution). Additionally, patients recruited to the interventional arm will be required to have serum IL-6 levels ≥0.7pg/ml and a Temporal Experience of Pleasure Scale (TEPS) anticipatory pleasure score ≤41 (based on item numbers 1, 3, 7, 11, 12, 14, 15, 16, 17, and 8) and consummatory pleasure score ≤36 (based on item numbers 2, 4, 5, 6, 8, 9, 10, and 13). Finally, COVID-19 anti-body titre test will be used to determine adequate levels of immune response via the following cut-offs (for poor response): 400IU Roche/700IU Abbot assay.

HCs will have no current or lifetime history of psychiatric diagnosis, as determined by the Mini-International Neuropsychiatric Interview (MINI). See Table 1 for complete inclusion and exclusion criteria. HCs will be matched to patient participants at the group level by age and sex.

For peer review only - http://bmjopen.bmj.com/site/about/guidelines.xhtml

Table 1. PIMS Trial inclusion and exclusion criteria.

Group	Inclusion criteria	Exclusion criteria
All participants	- Provide informed consent.<br>- Understand written and spoken English.<br>- Able and willing to consent to blood sampling.<br>- Willing to abstain from strenuous exercise for 72 hours prior to assessment.	- Pregnancy (confirmed by urine pregnancy test) or breast feeding.<br>- Body mass index >35.<br>- Current or lifetime diagnosis of antisocial personality disorder, autism or other neurodevelopmental disorder, major traumatic brain injury.<br>- Currently active diagnosed eating disorder likely to compromise ability to take part.<br>- History of alcohol or substance use disorder (abuse/dependence) within six months prior to eligibility assessment (nicotine and caffeine dependence are not exclusionary).<br>- Current use of medication likely to compromise interpretation of immunological data.<br>- Known active current or history of recurrent bacterial, viral, fungal, mycobacterial, or other opportunistic infections.<br>- Current infection with VZV, TB, Hepatitis B, Hepatitis C, or HIV confirmed by blood test. Chest X-ray will also be performed to assess for TB.<br>- Any major episode of infection requiring hospitalisation or treatment with IV antibiotics within 4 weeks of eligibility assessment.<br>- Unstable cardiac, pulmonary, renal, hepatic, endocrine, hematologic, or active infectious disease, including current or prior malignancy.<br>- Diverticulitis, inflammatory bowel disease, or uncontrolled gastric/duodenal ulcer.<br>- Concomitant auto-immune or auto-inflammatory rheumatological disease.<br>- Concomitant treatment with any biologic drugs.<br>- Current and active ischemic heart disease.<br>- Uncontrolled hypertension defined as systolic blood pressure > 170 or diastolic blood pressure > 110.<br>- History of severe allergic or anaphylactic reactions to human, humanized or murine monoclonal antibodies.<br>- No history of chicken pox infection or no history of varicella zoster immunity.

For peer review only

For peer review only - http://bmjopen.bmj.com/site/about/guidelines.xhtml

Additional criteria for neuroimaging (optional)	- Able and willing to consent to MRI scanning	- Contraindications to MRI.
Additional criterion for healthy controls	- No current or lifetime psychiatric diagnosis.	
Additional criteria for all individuals with psychosis	- Meet ICD-10 criteria for a diagnosis of schizophrenia and related psychoses (code F20, F22, F25, F28, F29) at the time of eligibility assessment, as determined by the Mini-International Neuropsychiatric Interview.<br>- Be within three years of first diagnosis of psychotic disorder.<br>- On stable treatment regime with no recent (within 2 weeks) initiation, cessation, or change in class of antipsychotic medication.<br>- No indication or other reason for preclusion into research (e.g., significant risk of suicidal behaviour or risk to others) as determined by their clinical team.<br>- Positive and Negative Syndrome Scale item score ≥3 on P1 (delusions), P2 (conceptual disorganisation), P3 (hallucinatory behaviour), OR P6 (suspiciousness/persecution).	
Additional criteria for intervention group	- Serum IL-6 level ≥0.7pg/ml at eligibility and baseline assessment.<br>- Temporal Experience of Pleasure Scale anticipatory pleasure score ≤41 (based upon item numbers 1, 3, 7, 11, 12, 14, 15, 16, 17, and 8) and consummatory pleasure score ≤36 (based upon item numbers 2, 4, 5, 6, 8, 9, 10, and 13).<br>- Evidence of COVID-19 immunity required prior to infusion, confirmed before randomisation using evidence of vaccination and antibody titre test.	
Additional criterion for patients with psychosis without inflammation	- Serum IL-6 level <0.7pg/ml at eligibility and baseline assessment.	

For peer review only

Study outcomes

The primary outcome is anhedonia, defined as anticipatory and consummatory pleasure scores, assessed by the TEPS [49] at approximately day 14 post-infusion. The primary mechanistic outcome is the level of IL-6/STAT3 signalling inhibition post-tocilizumab infusion in both innate and adaptive immune cells using multi-colour flow cytometry and an established optimised pSTAT3 phosflow assay. We will also collect data on several tertiary/exploratory measures including positive and negative symptoms of psychosis, depressive symptoms, fatigue, quality of life and subjective wellbeing, cognitive function, peripheral blood inflammatory markers, cortisol, cell expression, including DNA and RNA sequencing, functionality, and neuroimaging measures, including functional resting state MRI and MRS outcomes (Table 2).

For peer review only

Table 2. PIMS trial study measures

Domain	Tool	Source	Validated Tool	Time of assessment
Sociodemographic/lifestyle	Screening questionnaire	Self-report		Screening
	Medical History Questionnaire	Self-report/General practice		Eligibility
	Substance Use Questionnaire	Self-report		Eligibility
	Physical Measurements Form	Self-report		Baseline
	Sociodemographic Questionnaire	Self-report		Baseline
Psychiatric	The Temporal Experience of Pleasure Scale	Self-report	✓	Eligibility, baseline, follow-ups
	The Positive and Negative Syndrome Scale	Interviewer assessed	✓	Eligibility, baseline, follow-ups
	The Mini-International Neuropsychiatric Interview	Interviewer assessed	✓	Eligibility
	Psychiatric History Questionnaire	Self-report		Baseline

For peer review only - http://bmjopen.bmj.com/site/about/guidelines.xhtml

	The Scale for the Assessment of Negative Symptoms	Self-report	✓	Baseline, follow-ups
	The Calgary Depression Scale for Schizophrenia	Interviewer assessed	✓	Baseline, follow-ups
	Multi-dimensional Fatigue Inventory	Self-report	✓	Baseline, follow-ups
	European Quality of Life-5 Dimensions Three-Level Version	Self-report	✓	Baseline, follow-ups
	Visual Analogue Scale for Subjective Wellbeing	Self-report	✓	Baseline, follow-ups
Cognitive	National Adult Reading Test for estimated premorbid IQ	Interviewer assessed	✓	Baseline, follow-up 2
	CANTAB Reaction Time test	Computer task	✓	Baseline, follow-up 2
	Symbol Coding Test	Paper task	✓	Baseline, follow-up 2
	CANTAB Rapid Visual Information Processing test	Computer task	✓	Baseline, follow-up 2
	CANTAB Paired Associates Learning test	Computer task	✓	Baseline, follow-up 2
	CANTAB One Touch Stockings of Cambridge test	Computer task	✓	Baseline, follow-up 2
Biologic	Inflammatory markers, cardiometabolic markers, IDO activation, white cell phenotyping	Laboratory tests		Baseline, follow-ups
Genetic	Gene expression/genotyping	Blood (RNA, DNA)		Baseline, follow-ups
Neuroimaging	MRI Screening Questionnaire			Baseline, follow-up 2
	Structural MRI, 1H-MRS measure of glutathione in the prefrontal cortex area, resting state fMRI			Baseline, follow-up 2

For peer review only

Sample size and statistical power

We will recruit approximately 60 patients with psychosis. However, currently there are no trials of immunotherapies for anhedonia in schizophrenia making accurate power calculation difficult. This study is a proof-of-concept experiment designed to test whether inhibition of IL-6 signalling leads to changes in psychotic symptoms. It could also inform likely statistical power for future trials testing efficacy of the drug as a treatment of schizophrenia, which is not the intention of this study

Randomisation and blinding

An external agency independent of the study team will arrange random allocation to tocilizumab or normal saline group 1:1, ensuring two groups are comparable regarding anhedonia severity and sex. Randomisation will be stratified by site. Randomising agency will provide the randomisation code to the relevant hospital pharmacy who will dispense tocilizumab or normal saline according to the randomisation schedule. Dispensing pharmacies will keep a log of products dispensed. Infusions will be prepared and administered at clinical research facilities (CRFs). Infusion packs will be prepared by trained staff not part of the core study team, ensuring blinding of treatment allocation. Infusion packs containing drug or placebo will be visually indistinguishable from each other, ensuring that both participants and study team remain blind regarding treatment allocation.

Statistical analysis

For randomised participants, an intention-to-treat approach will be taken for data analysis by including all randomised participants in statistical analyses, regardless of the treatment they received (if any). We will compare outcome measures between treatment and placebo groups controlling for baseline scores. This mechanistic experiment will focus on overall pattern of

For peer review only

results and their effect sizes rather than *P*-values for individual tests of statistical significance. The secondary mechanistic and observational analysis will compare psychotic symptoms, cognitive function, blood, neuroimaging, and other biomarkers between and across study groups using appropriate statistical tests.

STUDY PROCEDURE

An overview of study procedures is presented in Figure 1 and all study measures are detailed in Table 2. Recruitment will take place in Bristol, Birmingham, and Cambridge and assessments at University and NHS research facilities.

Participant identification

Potential participants with psychosis will be identified by NHS Psychosis Early Intervention (EI) teams. HCs will be recruited through advertisement methods in Birmingham and Cambridge. Potential participants will complete a screening questionnaire to confirm their eligibility to participate. If deemed eligible, participants will be invited to an appointment to complete a full eligibility assessment.

Eligibility assessment

Assessments will be carried out to establish eligibility and to obtain informed consent. Patients will complete the MINI to confirm ICD-10 diagnosis of schizophrenia and related psychoses, the PANSS to confirm the presence of positive symptoms of psychosis, and the TEPS to confirm eligibility based on anticipatory and consummatory pleasure sum scores. A blood sample will be collected from patients for serum IL-6 measurement. An MRI screening questionnaire will be administered to those willing to give informed consent for neuroimaging.

For peer review only

For peer review only - http://bmjopen.bmj.com/site/about/guidelines.xhtml

Baseline assessment

All participants (60 inflamed psychosis, 30 non-inflamed psychosis, and 30 HCs) will attend a baseline assessment comprising psychiatric measures, cognitive tasks, blood sampling, and neuroimaging (optional). This will be the final study contact for patients without evidence of inflammation and HCs. Patients with evidence of inflammation will undergo further tests to establish safety/eligibility to receive tocilizumab, including a chest X-ray and blood tests to exclude pregnancy and certain infections, such as TB, HIV, and COVID-19. Eligible participants will be randomised and invited for infusion.

Intervention

Intravenous infusion of tocilizumab or normal saline will be given continuously over one hour at CRFs in Bristol, Birmingham, and Cambridge by trained clinical staff under the supervision of a designated study doctor. Participants will remain under clinical observation for a further 1-hour period after the end of infusion.

Follow-up assessments

Follow-up assessments will take place approximately 7-, 14-, and 28-days post-infusion, and will collect similar data to the baseline assessment. Cognitive tasks and neuroimaging (optional) will be administered only on day 14. Around 42 days post-infusion, participants will be contacted by phone to provide a final debrief; at which point they will exit the study.

RISK MANAGEMENT

Psychosis-related risks

All patients will be under the care of a specialist NHS psychosis EI service. Participation will not involve any treatment modifications or significant delays in receiving treatment. If a

For peer review only - http://bmjopen.bmj.com/site/about/guidelines.xhtml

patient becomes distressed during an assessment, or does not wish to continue for any reason, the researcher will stop the assessment. Participants may withdraw at any time without giving a reason. If there is any concern for the participant's safety, the research team will liaise with participant's GP and/or mental health team as needed.

Procedure-related risks

Venepuncture

Blood taking is associated with mild discomfort and other side effects are rare. Efforts will be made to minimise discomfort. Blood taking will be performed by a nurse, doctor, or research team member trained in venepuncture.

Chest X-ray

This study will use a typical effective radiation dose of 0.014 mSv; equivalent to 2.5 days of average natural background radiation in the UK. The risk of developing cancer as a consequence of participating in this study is 0.0001%. Only non-pregnant, adult participants will be included.

Neuroimaging

Discomfort during MRI will be minimised by using mirrors to allow participants to view outside of the machine, providing ear plugs and a panic button, and allowing participants to communicate with the researcher and scan operator throughout. Mild transient vertigo may be experienced when being moved into the MRI machine. Risk of dislodgement or malfunction of medical implants or metallic foreign objects will be minimised by screening participants to ensure no metal is present on or within the body.

For peer review only

For peer review only - http://bmjopen.bmj.com/site/about/guidelines.xhtml

IL-6 levels

Some participants will show evidence of inflammation in the blood (IL-6 ≥0.7pg/ml). This is not necessarily a cause for concern. In people with FEP, ~50% have serum IL-6 levels >0.7pg/ml. Reasons for this in the absence of an acute infection or chronic inflammatory illness could include obesity, smoking, alcohol use, and lack of exercise, so knowledge of 'inflammation status' may prompt participants to adopt a healthier lifestyle. If serum IL-6 level is high (i.e., IL-6 ≥0.7pg/ml) along with elevated CRP (>20mg/L) without any apparent explanation, such as infection or chronic inflammatory illness, we will inform the participant's GP and the participant will be excluded from the study.

Risk to research staff

Staff will follow local safety procedures when lone working. No other risks are anticipated.

Safety considerations for infusion and monitoring of adverse reaction

Before infusion

Participants will be selected based on strict inclusion and exclusion criteria. Additionally, we will carry out tests for TB, HIV, VZV antibody, and Hepatitis B and C because, though unlikely after a single dose, tocilizumab could make these infections worse. Female participants of childbearing age will be given a pregnancy test, which must be negative. Participants who are sexually active will be asked to use at least one form of effective contraception for six weeks post-infusion. Male participants will also be asked not to donate sperm samples for six weeks post-infusion.

For peer review only - http://bmjopen.bmj.com/site/about/guidelines.xhtml

During infusion

Infusions will be given under supervision of a designated study doctor. Participants will be monitored for possible side effects, which will be managed in line with use of tocilizumab for treating patients with RA.

After infusion

Participants will remain under observation for one-hour post-infusion. Participants will be advised to seek help if they feel unwell after leaving the assessment centre and will be given an information sheet containing a telephone number their health professionals can call. If necessary, we will unblind the participant and inform their health professional whether they received tocilizumab or normal saline. Adverse reactions will be recorded at each follow-up visit. Additional, safety blood tests will be done at second follow-up (e.g., WBC count, liver function, lipids).

ETHICS AND DISSEMINATION

The study will be conducted in accordance with the REC, Health Research Authority (HRA), and local Research and Development (R&D) department approvals and guidelines (REC reference: 22/EE/0010). The study team will prepare protocol amendments as required and ethics approval will be sought before implementing any changes to the approved protocol. The ISRCTN Trial Registry and the Research Governance Office will be informed of any amendments to the protocol.

Consent

Informed consent will be obtained prior to eligibility assessments for participation in the study. This will include consent to randomise, for contact with their GP to inform them about

For peer review only

participation, access GP/psychiatric records to verify medical history to establish eligibility, and to inform the participant's GP any results/outcomes as necessary. Consent for additional tests to establish safety for tocilizumab infusion and for storing biological samples will also be obtained.

Study management

The study is sponsored by the University of Bristol. The sponsor, the Chief Investigator (GMK), and the co-Lead (RU) will have overall responsibility for the study. A named principal investigator will take clinical responsibility for study activities at each site. The study does not require the formal arrangement of a steering committee because, according to the HRA, it is not a Clinical Trial of an Investigational Medicinal Product. However, to enhance monitoring of the study, a study management group will be established, comprising academic and clinical experts in psychiatry, rheumatology, neuroscience, and immunology.

Data management and retention of samples

All potential participants will be assigned a unique study-specific participant ID number. All data will be subject to good practice as laid down in the Data Protection Act. Each study stage is tracked so that participant's (de-identified) status within the study is known, and assessment and other appointment dates are forecasted. This information is held on a secure, password-protected database. Anonymised data from assessments will be uploaded to a secure, password-protected database using secure web-based data entry systems. Minimal personal data (age, sex) will be indexed by each participant's unique ID number. Blood samples collected in this study may be stored for up to 10 years after the completion for additional research. Stored samples will be coded throughout the sample storage and analysis

For peer review only - http://bmjopen.bmj.com/site/about/guidelines.xhtml

process and will not be labelled with personal identifiers. Participants may withdraw their consent for their samples to be stored for future research.

Dissemination plan

Study results will be published in peer-review journals and will conform to the guidelines of the International Committee of Medical Journal Editors. Findings will be disseminated at conferences, departmental talks, and via social and traditional media.

AUTHORS CONTRIBUTIONS

ÉMF wrote first draft of the PIMS trial protocol and of this manuscript. SLG, MK, GKM, BD, DJ, JS, and NMB contributed to study design and protocol development and revised manuscript drafts. RU contributed to study design and study protocol, and revised manuscript drafts. GMK devised study design and trial protocol, and revised drafts. ÉMF and SLG developed study materials and liaised with REC and HRA regarding approvals. AM, JR, FCZ, and HH contributed to the revision of the manuscript and validation of operating procedures and mechanistic protocols. RU and GMK co-lead the MRC grant that funds the PIMS trial and provide overall supervision and oversight for the project.

FUNDING STATEMENT

The PIMS trial is funded by a Medical Research Council (MRC) grant to RU and GMK; Grant Ref: MR/S037675/1. ÉMF is supported by an MRC Integrative Epidemiology Unit PhD Studentship. AM is supported by funding from the MRC for doctoral training (MR/2434208). FCZ receives a PhD Fellowship from the São Paulo Research Foundation (2019/13229-2 and 2021/07448-3). HH is supported by the PIMS Trial MRC grant (MR/S037675/1). DJ is supported by the Cambridge Arthritis Research Endeavour and the

For peer review only

468 National Institute for Health Research (NIHR) Cambridge Biomedical Research Centre
469 (BRC-1215-20014). NMB acknowledges funding support from the MRC (MR/R006008/1
470 and MR/N019016/1), Ministry of Defence (702931454), Diabetes UK (20/0006296), NIHR
471 (14/WM/0093), and Innovate UK (84361). RU has grants from MRC, NIHR: Health
472 Technology Assessment, European Commission - Research: The Seventh Framework
473 Programme, and personal fees from Sunovion, outside the submitted work. GMK
474 acknowledges funding support from the Wellcome Trust (Grant No. 201486/Z/16/Z). The
475 funders had no role in the design of this study.

477 COMPETING INTERESTS STATEMENT

478 ÉMF, SLG, AM, JR, FCZ, HH, MK, GKM, BD, DJ, JS, RU, and GMK have no conflicts of
479 interest to report. NMB holds shares and is a Director of Celentyx Ltd.

480

For peer review only

For peer review only - http://bmjopen.bmj.com/site/about/guidelines.xhtml

REFERENCES

1 Khandaker GM, Cousins L, Deakin J, *et al.* Inflammation and immunity in schizophrenia: implications for pathophysiology and treatment. *Lancet Psychiatry* 2015;**2**:258–70. doi:10.1016/S2215-0366(14)00122-9

2 Khandaker GM, Dantzer R. Is there a role for immune-to-brain communication in schizophrenia? *Psychopharmacology* 2016;**233**:1559–73. doi:10.1007/s00213-015-3975-1

3 Khandaker GM, Dantzer R, Jones PB. Immunopsychiatry: important facts. *Psychol Med* 2017;**47**:2229–37. doi:10.1017/S0033291717000745

4 Upthegrove R, Khandaker G. Cytokines, Oxidative Stress and Cellular Markers of Inflammation in Schizophrenia. *Current topics in behavioral neurosciences* 2020;**44**:49–66. doi:10.1007/7854_2018_88

5 Upthegrove R, Manzanares-Teson N, Barnes NM. Cytokine function in medication-naive first episode psychosis: a systematic review and meta-analysis. *Schizophr Res* 2014;**155**:101–8. doi:10.1016/j.schres.2014.03.005

6 Goldsmith DR, Rapaport MH, Miller BJ. A meta-analysis of blood cytokine network alterations in psychiatric patients: comparisons between schizophrenia, bipolar disorder and depression. *Mol Psychiatry* 2016;**21**:1696–709. doi:10.1038/mp.2016.3

7 Miller BJ, Buckley P, Seabolt W, *et al.* Meta-analysis of cytokine alterations in schizophrenia: clinical status and antipsychotic effects. *Biological psychiatry* 2011;**70**:663–71. doi:10.1016/j.biopsych.2011.04.013

8 Potvin S, Stip E, Sepehry AA, *et al.* Inflammatory cytokine alterations in schizophrenia: a systematic quantitative review. *Biol Psychiatry* 2008;**63**:801–8. doi:10.1016/j.biopsych.2007.09.024

9 Wang AK, Miller BJ. Meta-analysis of Cerebrospinal Fluid Cytokine and Tryptophan Catabolite Alterations in Psychiatric Patients: Comparisons Between Schizophrenia, Bipolar Disorder, and Depression. *Schizophr Bull* 2018;**44**:75–83. doi:10.1093/schbul/sbx035

10 Khandaker GM, Zimbron J, Dalman C, *et al.* Childhood infection and adult schizophrenia: a meta-analysis of population-based studies. *Schizophr Res* 2012;**139**:161–8. doi:10.1016/j.schres.2012.05.023

11 Khandaker GM, Zimbron J, Lewis G, *et al.* Prenatal maternal infection, neurodevelopment and adult schizophrenia: a systematic review of population-based studies. *Psychol Med* 2013;**43**:239–57. doi:10.1017/S0033291712000736

12 Khandaker GM, Zammit S, Burgess S, *et al.* Association between a functional interleukin 6 receptor genetic variant and risk of depression and psychosis in a population-based birth cohort. *Brain Behav Immun* 2018;**69**:264–72. doi:10.1016/j.bbi.2017.11.020

13 Perry BI, Upthegrove R, Kappelmann N, *et al.* Associations of immunological proteins/traits with schizophrenia, major depression and bipolar disorder: A bi-directional

For peer review only

two-sample mendelian randomization study. *Brain, Behavior, and Immunity* 2021;**97**:176–85. doi:10.1016/j.bbi.2021.07.009

14 Williams JA, Burgess S, Suckling J, *et al.* Inflammation and Brain Structure in Schizophrenia and Other Neuropsychiatric Disorders: A Mendelian Randomization Study. *JAMA Psychiatry* 2022;**79**:498. doi:10.1001/jamapsychiatry.2022.0407

15 Kose M, Pariante CM, Dazzan P, *et al.* The Role of Peripheral Inflammation in Clinical Outcome and Brain Imaging Abnormalities in Psychosis: A Systematic Review. *Front Psychiatry* 2021;**12**:612471. doi:10.3389/fpsyt.2021.612471

16 Lesh TA, Careaga M, Rose DR, *et al.* Cytokine alterations in first-episode schizophrenia and bipolar disorder: relationships to brain structure and symptoms. *Journal of Neuroinflammation* 2018;**15**:165. doi:10.1186/s12974-018-1197-2

17 Trépanier MO, Hopperton KE, Mizrahi R, *et al.* Postmortem evidence of cerebral inflammation in schizophrenia: a systematic review. *Mol Psychiatry* 2016;**21**:1009–26. doi:10.1038/mp.2016.90

18 van Kesteren CFMG, Gremmels H, de Witte LD, *et al.* Immune involvement in the pathogenesis of schizophrenia: a meta-analysis on postmortem brain studies. *Transl Psychiatry* 2017;**7**:e1075–e1075. doi:10.1038/tp.2017.4

19 Marques TR, Ashok AH, Pillinger T, *et al.* Neuroinflammation in schizophrenia: meta-analysis of in vivo microglial imaging studies. *Psychological Medicine* 2019;**49**:2186–96. doi:10.1017/S0033291718003057

20 Murray AJ, Rogers JC, Katshu MZUH, *et al.* Oxidative Stress and the Pathophysiology and Symptom Profile of Schizophrenia Spectrum Disorders. *Frontiers in Psychiatry* 2021;**12**.https://www.frontiersin.org/articles/10.3389/fpsyt.2021.703452 (accessed 9 Aug 2022).

21 Upthegrove R. The immune system and schizophrenia: An update for clinicians. *Advances in Psychiatric Treatment* 2014;**20**:83–91. doi:10.1192/apt.bp.113.011452

22 Conen S, Gregory CJ, Hinz R, *et al.* Neuroinflammation as measured by positron emission tomography in patients with recent onset and established schizophrenia: implications for immune pathogenesis. *Mol Psychiatry* 2021;**26**:5398–406. doi:10.1038/s41380-020-0829-y

23 van Mierlo HC, Schot A, Boks MPM, *et al.* The association between schizophrenia and the immune system: Review of the evidence from unbiased 'omic-studies.' *Schizophrenia Research* 2020;**217**:114–23. doi:10.1016/j.schres.2019.05.028

24 Plavén-Sigray P, Matheson GJ, Collste K, *et al.* Positron Emission Tomography Studies of the Glial Cell Marker Translocator Protein in Patients With Psychosis: A Meta-analysis Using Individual Participant Data. *Biological Psychiatry* 2018;**84**:433–42. doi:10.1016/j.biopsych.2018.02.1171

25 Plavén-Sigray P, Matheson GJ, Coughlin JM, *et al.* Meta-analysis of the Glial Marker TSPO in Psychosis Revisited: Reconciling Inconclusive Findings of Patient-Control Differences. *Biol Psychiatry* 2021;**89**:e5–8. doi:10.1016/j.biopsych.2020.05.028

For peer review only - http://bmjopen.bmj.com/site/about/guidelines.xhtml

26 Deakin B, Suckling J, Barnes TRE, *et al.* The benefit of minocycline on negative symptoms of schizophrenia in patients with recent-onset psychosis (BeneMin): a randomised, double-blind, placebo-controlled trial. *The Lancet Psychiatry* 2018;**5**:885–94. doi:10.1016/S2215-0366(18)30345-6

27 Weiser M, Levi L, Burshtein S, *et al.* The effect of minocycline on symptoms in schizophrenia: Results from a randomized controlled trial. *Schizophrenia Research* 2019;**206**:325–32. doi:10.1016/j.schres.2018.10.023

28 Ramaker RC, Bowling KM, Lasseigne BN, *et al.* Post-mortem molecular profiling of three psychiatric disorders. *Genome Medicine* 2017;**9**:72. doi:10.1186/s13073-017-0458-5

29 González-Peñas J, Costas J, Villamayor MJG, *et al.* Enrichment of rare genetic variants in astrocyte gene enriched co-expression modules altered in postmortem brain samples of schizophrenia. *Neurobiology of Disease* 2019;**121**:305–14. doi:10.1016/j.nbd.2018.10.013

30 Gandal MJ, Zhang P, Hadjimichael E, *et al.* Transcriptome-wide isoform-level dysregulation in ASD, schizophrenia, and bipolar disorder. *Science* 2018;**362**:eaat8127. doi:10.1126/science.aat8127

31 Gandal MJ, Haney JR, Parikshak NN, *et al.* Shared molecular neuropathology across major psychiatric disorders parallels polygenic overlap. *Science* 2018;**359**:693–7. doi:10.1126/science.aad6469

32 Toker L, Mancarci BO, Tripathy S, *et al.* Transcriptomic Evidence for Alterations in Astrocytes and Parvalbumin Interneurons in Subjects With Bipolar Disorder and Schizophrenia. *Biological Psychiatry* 2018;**84**:787–96. doi:10.1016/j.biopsych.2018.07.010

33 Corsi-Zuelli F, Deakin B. Impaired regulatory T cell control of astroglial overdrive and microglial pruning in schizophrenia. *Neuroscience & Biobehavioral Reviews* 2021;**125**:637–53. doi:10.1016/j.neubiorev.2021.03.004

34 Corsi-Zuelli F, Deakin B, de Lima MHF, *et al.* T regulatory cells as a potential therapeutic target in psychosis? Current challenges and future perspectives. *Brain, Behavior, & Immunity - Health* 2021;**17**:100330. doi:10.1016/j.bbih.2021.100330

35 Jeppesen R, Christensen RHB, Pedersen EMJ, *et al.* Efficacy and safety of anti-inflammatory agents in treatment of psychotic disorders – A comprehensive systematic review and meta-analysis. *Brain, Behavior, and Immunity* 2020;**90**:364–80. doi:10.1016/j.bbi.2020.08.028

36 Girgis RR, Ciarleglio A, Choo T, *et al.* A Randomized, Double-Blind, Placebo-Controlled Clinical Trial of Tocilizumab, An Interleukin-6 Receptor Antibody, For Residual Symptoms in Schizophrenia. *Neuropsychopharmacology : official publication of the American College of Neuropsychopharmacology* 2018;**43**:1317–23. doi:10.1038/npp.2017.258

For peer review only

For peer review only - http://bmjopen.bmj.com/site/about/guidelines.xhtml

37 Mondelli V, Ciufolini S, Belvederi Murri M, *et al.* Cortisol and Inflammatory Biomarkers Predict Poor Treatment Response in First Episode Psychosis. *Schizophrenia bulletin* 2015;**41**:1162–70. doi:10.1093/schbul/sbv028

38 Miller BJ, Culpepper N, Rapaport MH. C-reactive protein levels in schizophrenia: a review and meta-analysis. *Clinical schizophrenia & related psychoses* 2014;**7**:223–30. doi:10.3371/CSRP.MICU.020813

39 Raison CL, Rutherford RE, Woolwine BJ, *et al.* A randomized controlled trial of the tumor necrosis factor antagonist infliximab for treatment-resistant depression: The role of baseline inflammatory biomarkers. *JAMA Psychiatry* 2013;**70**:31–41. doi:10.1001/2013.jamapsychiatry.4

40 Khandaker GM, Stochl J, Zammit S, *et al.* Association between circulating levels of C-reactive protein and positive and negative symptoms of psychosis in adolescents in a general population birth cohort. *J Psychiatr Res* 2021;**143**:534–42. doi:10.1016/j.jpsychires.2020.11.028

41 Edwards CJ, Cella M, Tarrier N, *et al.* Investigating the empirical support for therapeutic targets proposed by the temporal experience of pleasure model in schizophrenia: A systematic review. *Schizophrenia research* 2015;**168**:120–44. doi:10.1016/j.schres.2015.08.013

42 Lalousis PA, Schmaal L, Wood SJ, *et al.* Neurobiologically Based Stratification of Recent-Onset Depression and Psychosis: Identification of Two Distinct Transdiagnostic Phenotypes. *Biological Psychiatry* 2022;:S0006322322011568. doi:10.1016/j.biopsych.2022.03.021

43 Fioravanti M, Carlone O, Vitale B, *et al.* A Meta-Analysis of Cognitive Deficits in Adults with a Diagnosis of Schizophrenia. *Neuropsychol Rev* 2005;**15**:73–95. doi:10.1007/s11065-005-6254-9

44 Keefe RSE, Bilder RM, Davis SM, *et al.* Neurocognitive Effects of Antipsychotic Medications in Patients With Chronic Schizophrenia in the CATIE Trial. *Archives of General Psychiatry* 2007;**64**:633–47. doi:10.1001/archpsyc.64.6.633

45 Chan A-W, Tetzlaff JM, Altman DG, *et al.* SPIRIT 2013 Statement: defining standard protocol items for clinical trials. *Rev Panam Salud Publica* 2015;**38**:506–14.

46 Calabrese LH, Rose-John S. IL-6 biology: implications for clinical targeting in rheumatic disease. *Nature reviews Rheumatology* 2014;**10**:720–7. doi:10.1038/nrrheum.2014.127

47 Choy EHS, Isenberg DA, Garrood T, *et al.* Therapeutic benefit of blocking interleukin-6 activity with an anti-interleukin-6 receptor monoclonal antibody in rheumatoid arthritis: a randomized, double-blind, placebo-controlled, dose-escalation trial. *Arthritis and rheumatism* 2002;**46**:3143–50. doi:10.1002/art.10623

48 Woo P, Wilkinson N, Prieur A-M, *et al.* Open label phase II trial of single, ascending doses of MRA in Caucasian children with severe systemic juvenile idiopathic arthritis: proof of principle of the efficacy of IL-6 receptor blockade in this type of arthritis and demonstration of prolonged clinical improvement. *Arthritis research & therapy* 2005;**7**:R1281-8. doi:10.1186/ar1826

For peer review only - http://bmjopen.bmj.com/site/about/guidelines.xhtml

49 Gard DE, Gard MG, Kring AM, *et al.* Anticipatory and consummatory components of the experience of pleasure: A scale development study. *Journal of Research in Personality* 2006;**40**:1086–102. doi:10.1016/j.jrp.2005.11.001

For peer review only - http://bmjopen.bmj.com/site/about/guidelines.xhtml

FIGURE LEGEND

Figure 1. Overview of study design

For peer review only

For peer review only - http://bmjopen.bmj.com/site/about/guidelines.xhtml

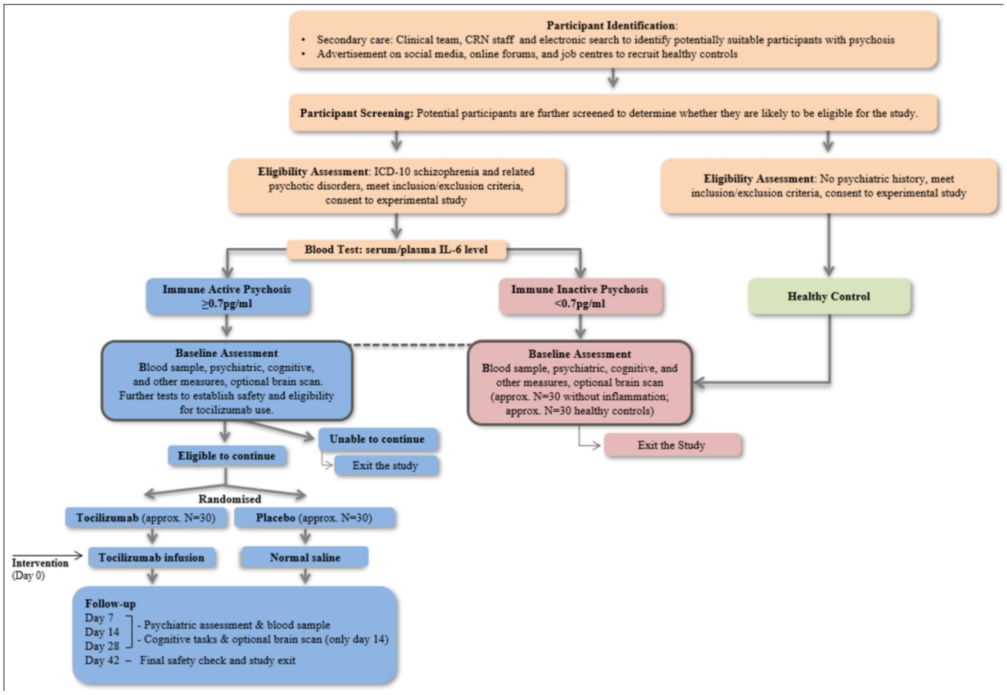

Figure 1. Overview of study design

240x165mm (144 x 144 DPI)

SPIRIT

STANDARD PROTOCOL ITEMS: RECOMMENDATIONS FOR INTERVENTIONAL TRIALS

Foley et al. Protocol for the Psychosis Immune Mechanism Stratified Medicine (PIMS) trial: A randomised double-blind placebo-controlled trial of single dose tocilizumab in patients with psychosis.

eTable1: SPIRIT 2013 Checklist – Recommended items to address in a clinical trial protocol and related documents*

Section/item	Item No	Description	Addressed on page number
**Administrative information**			
Title	1	Descriptive title identifying the study design, population, interventions, and, if applicable, trial acronym	1
Trial registration	2a	Trial identifier and registry name. If not yet registered, name of intended registry	4
	2b	All items from the World Health Organization Trial Registration Data Set	4 (ISRCTN 23256704)
Protocol version	3	Date and version identifier	-
Funding	4	Sources and types of financial, material, and other support	24 - 25
Roles and responsibilities	5a	Names, affiliations, and roles of protocol contributors	1, 24
	5b	Name and contact information for the trial sponsor	23
	5c	Role of study sponsor and funders, if any, in study design; collection, management, analysis, and interpretation of data; writing of the report; and the decision to submit the report for publication, including whether they will have ultimate authority over any of these activities	23-25

For peer review only

For peer review only - http://bmjopen.bmj.com/site/about/guidelines.xhtml

	5d	Composition, roles, and responsibilities of the coordinating centre, steering committee, endpoint adjudication committee, data management team, and other individuals or groups overseeing the trial, if applicable (see Item 21a for data monitoring committee)	23 - 25
**Introduction**			
Background and rationale	6a	Description of research question and justification for undertaking the trial, including summary of relevant studies (published and unpublished) examining benefits and harms for each intervention	6-9
	6b	Explanation for choice of comparators	6-9
Objectives	7	Specific objectives or hypotheses	9-10
Trial design	8	Description of trial design including type of trial (eg, parallel group, crossover, factorial, single group), allocation ratio, and framework (eg, superiority, equivalence, noninferiority, exploratory)	9
**Methods: Participants, interventions, and outcomes**			
Study setting	9	Description of study settings (e.g., community clinic, academic hospital) and list of countries where data will be collected. Reference to where list of study sites can be obtained	9, 11
Eligibility criteria	10	Inclusion and exclusion criteria for participants. If applicable, eligibility criteria for study centres and individuals who will perform the interventions (eg, surgeons, psychotherapists)	12, Table 1
Interventions	11a	Interventions for each group with sufficient detail to allow replication, including how and when they will be administered	11, 19, 21-22, Figure 1
	11b	Criteria for discontinuing or modifying allocated interventions for a given trial participant (eg, drug dose change in response to harms, participant request, or improving/worsening disease)	22
	11c	Strategies to improve adherence to intervention protocols, and any procedures for monitoring adherence (eg, drug tablet return, laboratory tests)	-
	11d	Relevant concomitant care and interventions that are permitted or prohibited during the trial	12, Table 1

For peer review only - http://bmjopen.bmj.com/site/about/guidelines.xhtml

Outcomes	12	Primary, secondary, and other outcomes, including the specific measurement variable (eg, systolic blood pressure), analysis metric (eg, change from baseline, final value, time to event), method of aggregation (eg, median, proportion), and time point for each outcome. Explanation of the clinical relevance of chosen efficacy and harm outcomes is strongly recommended	9-10, Figure 1, Table 2
Participant timeline	13	Time schedule of enrolment, interventions (including any run-ins and washouts), assessments, and visits for participants. A schematic diagram is highly recommended (see Figure)	18-19, Figure 1
Sample size	14	Estimated number of participants needed to achieve study objectives and how it was determined, including clinical and statistical assumptions supporting any sample size calculations	17
Recruitment	15	Strategies for achieving adequate participant enrolment to reach target sample size	18
**Methods: Assignment of interventions (for controlled trials)**			
Allocation:			
Sequence generation	16a	Method of generating the allocation sequence (eg, computer-generated random numbers), and list of any factors for stratification. To reduce predictability of a random sequence, details of any planned restriction (eg, blocking) should be provided in a separate document that is unavailable to those who enrol participants or assign interventions	17
Allocation concealment mechanism	16b	Mechanism of implementing the allocation sequence (eg, central telephone; sequentially numbered, opaque, sealed envelopes), describing any steps to conceal the sequence until interventions are assigned	17
Implementation	16c	Who will generate the allocation sequence, who will enrol participants, and who will assign participants to interventions	17
Blinding (masking)	17a	Who will be blinded after assignment to interventions (eg, trial participants, care providers, outcome assessors, data analysts), and how	17
	17b	If blinded, circumstances under which unblinding is permissible, and procedure for revealing a participant's allocated intervention during the trial	22

**Methods: Data collection, management, and analysis**			
Data collection methods	18a	Plans for assessment and collection of outcome, baseline, and other trial data, including any related processes to promote data quality (eg, duplicate measurements, training of assessors) and a description of study instruments (eg, questionnaires, laboratory tests) along with their reliability and validity, if known. Reference to where data collection forms can be found, if not in the protocol	10-15, 18-19, Table 2
	18b	Plans to promote participant retention and complete follow-up, including list of any outcome data to be collected for participants who discontinue or deviate from intervention protocols	17-18
Data management	19	Plans for data entry, coding, security, and storage, including any related processes to promote data quality (eg, double data entry; range checks for data values). Reference to where details of data management procedures can be found, if not in the protocol	23-24
Statistical methods	20a	Statistical methods for analysing primary and secondary outcomes. Reference to where other details of the statistical analysis plan can be found, if not in the protocol	17-18
	20b	Methods for any additional analyses (eg, subgroup and adjusted analyses)	17-18
	20c	Definition of analysis population relating to protocol non-adherence (eg, as randomised analysis), and any statistical methods to handle missing data (eg, multiple imputation)	-
**Methods: Monitoring**			
Data monitoring	21a	Composition of data monitoring committee (DMC); summary of its role and reporting structure; statement of whether it is independent from the sponsor and competing interests; and reference to where further details about its charter can be found, if not in the protocol. Alternatively, an explanation of why a DMC is not needed	23
	21b	Description of any interim analyses and stopping guidelines, including who will have access to these interim results and make the final decision to terminate the trial	-
Harms	22	Plans for collecting, assessing, reporting, and managing solicited and spontaneously reported adverse events and other unintended effects of trial interventions or trial conduct	20-22

For peer review only - http://bmjopen.bmj.com/site/about/guidelines.xhtml

Auditing	23	Frequency and procedures for auditing trial conduct, if any, and whether the process will be independent from investigators and the sponsor	-
**Ethics and dissemination**			
Research ethics approval	24	Plans for seeking research ethics committee/institutional review board (REC/IRB) approval	22
Protocol amendments	25	Plans for communicating important protocol modifications (eg, changes to eligibility criteria, outcomes, analyses) to relevant parties (eg, investigators, REC/IRBs, trial participants, trial registries, journals, regulators)	22
Consent or assent	26a	Who will obtain informed consent or assent from potential trial participants or authorised surrogates, and how (see Item 32)	22-23
	26b	Additional consent provisions for collection and use of participant data and biological specimens in ancillary studies, if applicable	22-23
Confidentiality	27	How personal information about potential and enrolled participants will be collected, shared, and maintained in order to protect confidentiality before, during, and after the trial	23-24
Declaration of interests	28	Financial and other competing interests for principal investigators for the overall trial and each study site	25
Access to data	29	Statement of who will have access to the final trial dataset, and disclosure of contractual agreements that limit such access for investigators	23
Ancillary and post-trial care	30	Provisions, if any, for ancillary and post-trial care, and for compensation to those who suffer harm from trial participation	22
Dissemination policy	31a	Plans for investigators and sponsor to communicate trial results to participants, healthcare professionals, the public, and other relevant groups (eg, via publication, reporting in results databases, or other data sharing arrangements), including any publication restrictions	24
	31b	Authorship eligibility guidelines and any intended use of professional writers	24
	31c	Plans, if any, for granting public access to the full protocol, participant-level dataset, and statistical code	-

For peer review

**Appendices**			
Informed consent materials	32	Model consent form and other related documentation given to participants and authorised surrogates	Available in full protocol
Biological specimens	33	Plans for collection, laboratory evaluation, and storage of biological specimens for genetic or molecular analysis in the current trial and for future use in ancillary studies, if applicable	23-24

*It is strongly recommended that this checklist be read in conjunction with the SPIRIT 2013 Explanation & Elaboration for important clarification on the items. Amendments to the protocol should be tracked and dated. The SPIRIT checklist is copyrighted by the SPIRIT Group under the Creative Commons "Attribution-NonCommercial-NoDerivs 3.0 Unported" license.

For peer review only

BMJ Open

Protocol for the Psychosis Immune Mechanism Stratified Medicine (PIMS) trial: A randomised double-blind placebo-controlled trial of single dose tocilizumab in patients with psychosis

Journal:	*BMJ Open*
Manuscript ID	bmjopen-2022-067944.R1
Article Type:	Protocol
Date Submitted by the Author:	28-Nov-2022
Complete List of Authors:	Foley, Éimear; University of Bristol, MRC Integrative Epidemiology Unit, Population Health Sciences; Bristol Medical School, Centre for Academic Mental Health, Population Health Sciences<br>Griffiths, Sian Lowri; University of Birmingham, Institute for Mental Health and Centre for Human Brain Health<br>Murray, Alexander; University of Birmingham, Institute for Mental Health and Centre for Human Brain Health<br>Rogers, Jack; University of Birmingham, Institute for Mental Health and Centre for Human Brain Health<br>Corsi-Zuelli, Fabiana; University of Birmingham, Institute for Mental Health and Centre for Human Brain Health; University of Sao Paulo, Department of Neuroscience and Behaviour<br>Hickinbotham, Hannah; University of Cambridge, Department of Psychiatry<br>Warwick, Ella; University of Birmingham, Institute for Mental Health and Centre for Human Brain Health<br>Wilson, Martin; University of Birmingham, Institute for Mental Health and Centre for Human Brain Health<br>Kaser, Muzaffer; University of Cambridge, Psychiatry; Cambridgeshire and Peterborough NHS Foundation Trust<br>Murray, Graham K.; University of Cambridge, Psychiatry; Cambridgeshire and Peterborough NHS Foundation Trust<br>Deakin, Bill; The University of Manchester, Faculty of Medicine, Biology and Health<br>Jadon, Deepak; University of Cambridge, Medicine<br>Suckling, John; University of Cambridge, Psychiatry; Cambridgeshire and Peterborough NHS Foundation Trust<br>Barnes, Nicholas ; University of Birmingham, Institute of Clinical Sciences<br>Upthegrove, Rachel; University of Birmingham, Institute for Mental Health and Centre for Human Brain Health; Early Intervention Service, Birmingham Women's and Children's NHS Foundation Trust<br>Khandaker, Golam M.; University of Bristol, MRC Integrative Epidemiology Unit, Population Health Sciences; NIHR Bristol Biomedical Research Centre
**Primary Subject Heading**:	Mental health

Secondary Subject Heading:	Immunology (including allergy)
Keywords:	Schizophrenia & psychotic disorders < PSYCHIATRY, IMMUNOLOGY, Clinical trials < THERAPEUTICS, Magnetic resonance imaging < RADIOLOGY & IMAGING

SCHOLARONE™
Manuscripts

Protocol for the Psychosis Immune Mechanism Stratified Medicine (PIMS) trial: A randomised double-blind placebo-controlled trial of single dose tocilizumab in patients with psychosis

Éimear M. Foley[a,b]; Sian Lowri Griffiths[c]; Alexander Murray[c]; Jack Rogers[c]; Fabiana Corsi-Zuelli[c,d,e]; Hannah Hickinbotham[f]; Ella Warwick[c]; Martin Wilson[c]; Muzaffer Kaser[f,g]; Graham K. Murray[f,g]; Bill Deakin[h]; Deepak Jadon[i]; John Suckling[f,g]; Nicholas M. Barnes[e;] Rachel Upthegrove[c,j,†]; and Golam M. Khandaker[a,b,k,l,†,*]; for the PIMS Collaboration

[a] MRC Integrative Epidemiology Unit, Population Health Sciences, Bristol Medical School, University of Bristol, Bristol, United Kingdom

[b] Centre for Academic Mental Health, Population Health Sciences, Bristol Medical School, University of Bristol, Bristol, United Kingdom

[c] Institute for Mental Health and Centre for Human Brain Health, University of Birmingham, Birmingham, United Kingdom

[d] Department of Neuroscience and Behaviour, Division of Psychiatry, Ribeirão Preto Medical School, University of São Paulo, São Paulo, Brazil

[e] Institute of Clinical Sciences, College of Medical and Dental Sciences, University of Birmingham, United Kingdom

[f] Department of Psychiatry, University of Cambridge, United Kingdom

[g] Cambridgeshire and Peterborough NHS Foundation Trust, United Kingdom

[h] Faculty of Biology, Medicine and Health, Division of Neuroscience and Experimental Psychology, School of Biological Sciences, University of Manchester, Manchester Academic Health Science Centre, Manchester, United Kingdom

[i] Department of Medicine, University of Cambridge, United Kingdom

For peer review only

[j] Early Intervention Service, Birmingham Women's and Children's NHS Foundation Trust

[k] NIHR Bristol Biomedical Research Centre, Bristol, United Kingdom

[l] Avon and Wiltshire Mental Health Partnership NHS Trust, Bristol, United Kingdom

† Joint senior authors

* **Corresponding author and address:** Prof Golam M. Khandaker, MRC Integrative Epidemiology Unit, Oakfield House, Oakfield Grove, Clifton, Bristol, BS8 2BN, UK

Email: golam.khandaker@bristol.ac.uk

For peer review only

ABSTRACT

Introduction: Evidence suggests a potentially causal role of interleukin 6 (IL-6), a pleiotropic cytokine that generally promotes inflammation, in the pathogenesis of psychosis, but no interventional studies elucidating potential mechanisms in patients with psychosis, stratified using inflammatory markers, have been conducted. Tocilizumab is a humanised monoclonal antibody targeting the IL-6 receptor to inhibit IL-6 signalling licensed in the UK for treatment of rheumatoid arthritis. The primary objective of this study is to test whether IL-6 contributes to the pathogenesis of psychosis, and to examine potential mechanisms by which IL-6 affects psychotic symptoms. A secondary objective is to examine characteristics of inflammation-associated psychosis.

Methods and analysis: A proof-of-concept study employing a randomised, parallel-group, double-blind, placebo-controlled design testing the effect of IL-6 inhibition on anhedonia in patients with psychosis. Approximately 60 participants with diagnosis of schizophrenia and related psychotic disorders (ICD-10 codes F20, F22, F25, F28, F29) with evidence of low-grade inflammation (IL-6 ≥0.7pg/ml) will receive either one intravenous infusion of tocilizumab (4.0mg/kg; max 800mg) or normal saline. Psychiatric measures and blood samples will be collected at baseline, and 7-, 14-, and 28-days post-infusion. Cognitive and neuroimaging data will be collected at baseline and 14 days post-infusion. In addition, approximately 30 patients with psychosis without evidence of inflammation (IL-6 <0.7pg/ml) and 30 matched healthy controls will be recruited to complete identical baseline assessments to allow for comparison of the characteristic features of inflammation-associated psychosis.

Ethics and dissemination: The study is sponsored by the University of Bristol and has been approved by the Cambridge East Research Ethics Committee (reference: 22/EE/0010; IRAS project ID: 301682). Study findings will be published in peer-review journals. Findings will be also disseminated by scientific presentation and by other means.

For peer review only

Trial registration number: ISRCTN 23256704

KEYWORDS: Psychotic Disorders; Negative Symptoms; Interleukin 6; Immunotherapy; Tocilizumab; Clinical Trial.

For peer review only

ARTICLE SUMMARY

Strengths and limitations of this study

- Adopting a randomised controlled trial (RCT) design and patient selection based on elevated level of IL-6 (in addition to other criteria) will help examine the causal role of IL-6, and the therapeutic potential of targeting IL-6 pathway, in psychosis.
- The use of target specific intervention (anti-IL6R monoclonal antibody tocilizumab) will help assess the clinical relevance of IL-6 and related up- and downstream inflammatory cytokines in psychosis.
- The use of neuroimaging, cognitive tests, and extensive peripheral blood biomarker exploration before and after tocilizumab treatment to assess potential mechanisms of effect.
- One dose of tocilizumab is unlikely to be sufficient to test the efficacy of this drug as potential treatment for psychosis.
- Tocilizumab inhibits both anti-inflammatory (classic) and pro-inflammatory (trans) pathways of IL-6 that may have complementary or differential effects relevant to potential therapeutic effects.

For peer review only - http://bmjopen.bmj.com/site/about/guidelines.xhtml

Word count: 4,245

INTRODUCTION

Scientific background and study rationale

The neuroimmune hypothesis of schizophrenia proposes that mild peripheral immune activation gives rise to an inflammatory response in the brain and neurobiological changes associated with psychotic illness [1–4]. Meta-analytic evidence is clear that circulating concentrations of interleukin 6 (IL-6) and other inflammatory proteins, such as C-reactive protein (CRP), are increased in patients with psychosis, including treatment naïve first episode psychosis (FEP) [5], compared with controls [6–9]. Prospective cohort studies show that these indices of mild immune activation precede the onset of symptoms [10,11]. Furthermore, genetic variants known to increase IL-6 concentrations are associated with genetic risk of schizophrenia [12,13]. These Mendelian randomization studies eliminate the possibility that raised IL-6 concentrations are a consequence of environmental exposures associated with schizophrenia, such as obesity and smoking and instead suggest that IL-6 has a causal role in psychosis. Extending this approach using the UK Biobank population, we found that genetically-predicted levels of IL-6 were associated with reduced grey matter primarily in the middle temporal gyrus, a region whose gene expression profile is enriched for IL-6 pathway proteins and for neuropsychiatric disorder ontologies [14]. Moreover, clinical studies report correlations between IL-6 levels and structural brain changes in individuals with schizophrenia [15], with reduced grey matter volume being exaggerated in patients with psychosis and elevated inflammatory cytokines [16]. Though this causal evidence strongly implicates IL-6, only an intervention study in patients can test the causal hypothesis.

The neuroimmune hypothesis generally assumes that microglia, the brain's resident immune cells, are activated and pathogenic in schizophrenia. This is supported by traditional

For peer review only

For peer review only - http://bmjopen.bmj.com/site/about/guidelines.xhtml

neuropathological studies and initial in-vivo PET imaging studies [17–19], possibly reflecting impaired cellular control of inflammation or oxidative defence. Inflammatory damage may also account for evidence of oxidative stress from MRS glutathione studies [20]. However, whether microglia are the direct target of IL-6 is unclear and it is not certain that IL-6 can cross the blood-brain barrier and/or increase its permeability to circulating inflammatory cells, cytokines, and chemokines [2,21]. Additionally, it is increasingly uncertain whether microglial inflammation, as traditionally understood, occurs in schizophrenia [22,23]. Recent meta-analyses of PET radioligand binding studies report decreased rather than increased radioligand binding to activated microglia [24,25]. This may account for the unexpected lack of therapeutic benefit of the anti-microglial antibiotic, minocycline, in recent large clinical trials [26,27]. Furthermore, large transcriptomic studies in post-mortem brains report no change or reduction in microglial gene expression but increases in astrocytic expression [23,28–32]. It is increasingly understood that both peripheral immune responses and brain glial function are regulated by specialised T cells (Tregs), a subset of which reside in brain parenchyma [33,34]. A novel proposal is that Treg hypofunction accounts for mild peripheral immune disinhibition and dysregulated astroglial-microglial interaction, such that microglia are driven into a developmental, synapse-pruning phenotype while astroglia disrupt neurotransmitter function [33,34]. Importantly, there are bidirectional interactions between IL-6 and Treg function [34]. Crucially, we will measure IL-6 in addition to cellular and molecular markers of immune function and investigate how they correlate with central markers and clinical state.

Previous attempts testing the inflammatory hypothesis in therapeutic clinical trials have been attempted. However, little evidence of overall efficacy has been found [35]. These trials have generally tested broad spectrum agents, such as non-steroidal anti-inflammatory drugs, with no attempt to stratify patients according to evidence of inflammation. A trial

For peer review only

For peer review only - http://bmjopen.bmj.com/site/about/guidelines.xhtml

using tocilizumab, a humanised monoclonal antibody (mAb) against the IL-6 receptor currently licensed in the UK for treatment of rheumatoid arthritis (RA) and severe coronavirus disease, reported no improvements in any clinical measure in a small sample of 36 patients with established schizophrenia [36]. However, as mentioned previously, no stratification by inflammatory markers or any mechanistic immune measures was applied. Low-grade inflammation is associated with poor response to antipsychotic drugs [37], but immunotherapy is unlikely to be relevant for all patients with psychosis. Meta-analysis suggests that evidence of immune activation, defined by elevated CRP levels, is present in a quarter to one third of patients with schizophrenia [38]. A randomised controlled trial of infliximab, an anti-tumour necrosis factor alpha (TNF-α) mAb, reported that antidepressant response was associated with higher CRP levels at baseline [39], suggesting that patients with evidence of immune activation may be better candidates for immunotherapy trials. As far as we are aware, no previous clinical trial has selected patients with schizophrenia based on evidence of immune activation.

Selection of patients with particular symptom profiles and/or stage of illness may also be a useful strategy that needs to be employed in immunotherapy trials for schizophrenia. A wide variety of symptoms occur in schizophrenia such as hallucinations, delusions, anhedonia, cognitive dysfunction, and affective symptoms and presentation of these symptoms differ from one individual to another. Some symptoms may be more related to inflammation than others. For instance, meta-analytic data suggests that elevated proinflammatory cytokines are associated with negative psychotic symptomatology [40]. Moreover, a recent study from the ALSPAC birth cohort reported that out of 20 positive and negative symptoms, CRP is particularly associated with anhedonia and auditory hallucinations [41]. Lastly, results from work we have completed to date as part of the MRC-funded larger PIMS collaboration (MR/S037675/1), suggest that anhedonia may be a

For peer review only - http://bmjopen.bmj.com/site/about/guidelines.xhtml

promising target in early phases of established psychotic disorder. Anhedonia and amotivation are strongly associated with poor functional outcomes in depression and schizophrenia, and present a formidable barrier to returning to work or building relationships [42,43]. Patients with psychotic disorders also present with cognitive deficits in a range of domains [44]. Available antipsychotic medications have a limited effects on poor cognitive functioning in psychosis [45]. Illness stage may also be of relevance. Meta-analytic data has revealed no differences in IL-6 levels between stable, medicated patients with schizophrenia and controls, although compared with controls, IL-6 levels were similarly elevated in patients with FEP and those with acute relapse [7]. A separate meta-analysis found evidence of elevated blood cytokine levels in acutely and chronically ill patients with schizophrenia [6]. Focusing on particular inflammation-related symptoms, such as anhedonia, and/or illness stage may increase the chance of success for immunotherapy trials.

Proposed study

The proposed two-year study is a UK multi-site (Bristol, Birmingham, and Cambridge) proof-of-concept, randomised, parallel-group, double-blind, placebo-controlled trial.

Study aims and hypotheses

The primary aim of this trial is to examine potential mechanisms by which IL-6 affects anhedonia, psychotic symptoms, and cognition. Our primary hypothesis is that inhibition of IL-6 signalling with a single intravenous infusion of anti-IL6R monoclonal antibody, tocilizumab, in individuals with psychosis and elevated IL-6 at baseline will attenuate symptoms of anhedonia and amotivation in patients with psychosis, relative to placebo. This will provide further evidence for a potential causal role of inflammation in psychosis. Our secondary hypothesis is that reduction in peripheral inflammation after tocilizumab infusion

For peer review only

in patients with psychosis and evidence of inflammation will be associated with central measures of oxidative stress and relevant resting state brain function.

We will also conduct deep immunophenotyping of peripheral blood mononuclear cell subsets ($CD4^+$, $CD8^+$, Tregs, natural killer and natural killer-T cells, monocytes, and B cells) to characterise their absolute number, frequency, and function. Our primary mechanistic outcome is the level of IL-6/STAT3 signalling inhibition within both innate and adaptive immune cells using multi-colour flow cytometry with an established optimised pSTAT3 phosflow assay. This will help identify the potential cellular impact of peripheral inflammation in psychosis, which is largely unknown. Functional assessment of IL-6/STAT3 signalling in immune cell subsets and their response to exogenous IL-6 stimulation will inform abnormal immune response in psychosis and allow measurement of response to tocilizumab at the cellular level.

A secondary objective is to carry out an observational study to examine clinical and biomarker differences and similarities between patients with psychotic disorder with and without evidence of inflammation and healthy controls (HCs). We hypothesise that individuals with psychotic disorder and evidence of inflammation, compared to those without evidence of inflammation and HCs, will have increased symptoms of anhedonia and amotivation, poorer cognitive functioning, and cellular and brain-based measures of immune dysfunction.

METHODS

This protocol has been prepared in accordance with the Standard Protocol Items: Recommendations for Interventional Trials (SPIRIT) 2013 statement [46]. Please see supplementary eTable 1 for the SPIRIT checklist. The planned start date for the PIMS trial was 1st November 2021, however, this was delayed due to the COVID-19 pandemic. We

For peer review only

For peer review only - http://bmjopen.bmj.com/site/about/guidelines.xhtml

began recruiting at our site in Birmingham in November 2022, and we soon expect recruitment to begin at our Bristol and Cambridge sites. The planned end date is 31st May 2024.

Patient and public involvement

The study protocol was prepared in collaboration with individuals with lived experience of mental illness who contributed to the development of participant information sheet, consent forms (Appendix I, II, and III), and data collection procedures.

Study design and sample

See Figure 1 for an overview of study design. Individuals residing in Bristol, Birmingham, or Cambridge in the United Kingdom will be recruited. Approximately 60 participants with psychosis and evidence of inflammation (i.e., IL-6 ≥0.7pg/ml) will be randomised to receive either one intravenous infusion of tocilizumab (drug) or normal saline (placebo). For the secondary, observational study, we will compare baseline characteristics of the intervention cohort with approximately 30 participants with psychosis without evidence of inflammation (i.e., IL-6 <0.7pg/ml), and approximately 30 HCs. Participants without evidence of inflammation and controls will not be randomised as they will not receive any intervention. Neuroimaging will only be undertaken by those without MRI contraindications who have given specific informed consent for MRI. Participants not eligible or not consenting for MRI will take part in all other aspects of the study.

Intervention

Single intravenous infusion of tocilizumab (4.0mg/kg; max 800mg in total) or normal saline given to participants with psychosis and evidence of inflammation. Tocilizumab blocks both

For peer review only

For peer review only - http://bmjopen.bmj.com/site/about/guidelines.xhtml

IL-6 classic and trans-signalling – the latter being responsible for most of the inflammatory effects of IL-6 – providing broad inhibition of IL-6 signalling and a strong test of a casual role for IL-6 in psychosis [47]. Tocilizumab is the first-in-class, humanized monoclonal antibody against the IL-6R, commercially available and licensed in the UK for treatment of RA. Approved dosage of tocilizumab for treatment of RA is 2, 4, or 8mg/kg; max 800mg in total. In RA, a single tocilizumab infusion has shown to improve clinical and laboratory measures within 48 hours, with most noticeable results in one-to-two weeks [48,49]. The follow-up schedule for our study is in keeping with this observation.

For peer review only

Eligibility criteria

We will recruit participants aged 18-40 years. Patient participants must meet International Classification of Diseases 10th Revision (ICD-10) criteria for a diagnosis of schizophrenia and related psychoses (ICD-10 code F20, F22, F25, F28, F29) at the time of eligibility assessment, be within three years of first diagnosis of psychotic disorder, be on a stable treatment regime with no recent (within two weeks) initiation, cessation, or change in class of antipsychotic medication, and have a Positive and Negative Syndrome Scale (PANSS) item score ≥3 on P1 (delusions), P2 (conceptual disorganisation), P3 (hallucinatory behaviour), or P6 (suspiciousness/persecution). Additionally, patients recruited to the interventional arm will be required to have serum IL-6 levels ≥0.7pg/ml and a Temporal Experience of Pleasure Scale (TEPS) anticipatory pleasure score ≤41 (based on item numbers 1, 3, 7, 11, 12, 14, 15, 16, 17, and 8) and consummatory pleasure score ≤36 (based on item numbers 2, 4, 5, 6, 8, 9, 10, and 13). The threshold of serum IL-6 ≥0.7pg/mL as evidence of inflammation for this particular trial was chosen based on observations from the Personalised Prognostic Tools for Early Psychosis Management (PRONIA) cohort [https://www.pronia.eu]. In 192 first-episode psychosis patients included in the PRONIA study, the median value of serum IL-6 was

For peer review only - http://bmjopen.bmj.com/site/about/guidelines.xhtml

0.49pg/mL (25th percentile 0.22pg/mL; 75th percentile 1.11pg/mL), and the mean was 0.79pg/mL (SD ± 0.84). Based on these observations, we chose the cut-off of 0.7pg/mL for patient selection in current trial. Finally, COVID-19 anti-body titre test will be used to determine adequate levels of immune response via the following cut-offs (for poor response): 400IU Roche/700IU Abbot assay.

HCs will have no current or lifetime history of psychiatric diagnosis, as determined by the Mini-International Neuropsychiatric Interview (MINI). See Table 1 for complete inclusion and exclusion criteria. HCs will be matched to patient participants at the group level by age and sex.

Table 1. PIMS Trial inclusion and exclusion criteria.

Group	Inclusion criteria	Exclusion criteria
All participants	- Provide informed consent.<br>- Understand written and spoken English.<br>- Able and willing to consent to blood sampling.<br>- Willing to abstain from strenuous exercise for 72 hours prior to assessment.	- Pregnancy (confirmed by urine pregnancy test) or breast feeding.<br>- Body mass index >35.<br>- Current or lifetime diagnosis of antisocial personality disorder, autism or other neurodevelopmental disorder, major traumatic brain injury.<br>- Currently active diagnosed eating disorder likely to compromise ability to take part.<br>- History of alcohol or substance use disorder (abuse/dependence) within six months prior to eligibility assessment (nicotine and caffeine dependence are not exclusionary).<br>- Current use of medication likely to compromise interpretation of immunological data.<br>- Known active current or history of recurrent bacterial, viral, fungal, mycobacterial, or other opportunistic infections.<br>- Current infection with VZV, TB, Hepatitis B, Hepatitis C, or HIV confirmed by blood test. Chest X-ray will also be performed to assess for TB.<br>- Any major episode of infection requiring hospitalisation or treatment with IV antibiotics within 4 weeks of eligibility assessment.

For peer review only

For peer review only - http://bmjopen.bmj.com/site/about/guidelines.xhtml

		- Unstable cardiac, pulmonary, renal, hepatic, endocrine, hematologic, or active infectious disease, including current or prior malignancy.<br>- Diverticulitis, inflammatory bowel disease, or uncontrolled gastric/duodenal ulcer.<br>- Concomitant auto-immune or auto-inflammatory rheumatological disease.<br>- Concomitant treatment with any biologic drugs.<br>- Current and active ischemic heart disease.<br>- Uncontrolled hypertension defined as systolic blood pressure > 170 or diastolic blood pressure > 110.<br>- History of severe allergic or anaphylactic reactions to human, humanized or murine monoclonal antibodies.<br>- No history of chicken pox infection or no history of varicella zoster immunity.
Additional criteria for neuroimaging (optional)	- Able and willing to consent to MRI scanning	- Contraindications to MRI.
Additional criterion for healthy controls	- No current or lifetime psychiatric diagnosis.	
Additional criteria for all individuals with psychosis	- Meet ICD-10 criteria for a diagnosis of schizophrenia and related psychoses (code F20, F22, F25, F28, F29) at the time of eligibility assessment, as determined by the Mini-International Neuropsychiatric Interview.<br>- Be within three years of first diagnosis of psychotic disorder.<br>- On stable treatment regime with no recent (within 2 weeks) initiation, cessation, or change in class of antipsychotic medication.<br>- No indication or other reason for preclusion into research (e.g., significant risk of suicidal behaviour or risk to others) as determined by their clinical team.<br>- Positive and Negative Syndrome Scale item score ≥3 on P1 (delusions), P2 (conceptual disorganisation), P3 (hallucinatory behaviour), OR P6 (suspiciousness/persecution).	

For peer review only - http://bmjopen.bmj.com/site/about/guidelines.xhtml

Additional criteria for intervention group	- Serum IL-6 level ≥0.7pg/ml at eligibility and baseline assessment.<br>- Temporal Experience of Pleasure Scale anticipatory pleasure score ≤41 (based upon item numbers 1, 3, 7, 11, 12, 14, 15, 16, 17, and 8) and consummatory pleasure score ≤36 (based upon item numbers 2, 4, 5, 6, 8, 9, 10, and 13).<br>- Evidence of COVID-19 immunity required prior to infusion, confirmed before randomisation using evidence of vaccination and antibody titre test.	
Additional criterion for patients with psychosis without inflammation	- Serum IL-6 level <0.7pg/ml at eligibility and baseline assessment.	

For peer review only

Study outcomes

The primary outcome is anhedonia, defined as anticipatory and consummatory pleasure scores, assessed by the TEPS [50] at approximately day 14 post-infusion. The primary mechanistic outcome is the level of IL-6/STAT3 signalling inhibition post-tocilizumab infusion in both innate and adaptive immune cells using multi-colour flow cytometry and an established optimised pSTAT3 phosflow assay. We will also collect data on several tertiary/exploratory measures including positive and negative symptoms of psychosis, depressive symptoms, fatigue, quality of life and subjective wellbeing, cognitive function (psychomotor speed, attention and memory, and executive function), peripheral blood inflammatory markers, cortisol, cell expression, including DNA and RNA sequencing, functionality, and neuroimaging measures, including functional resting state MRI and MRS outcomes (Table 2). Where possible, blood samples will be collected during working hours

For peer review only - http://bmjopen.bmj.com/site/about/guidelines.xhtml

and time of sampling will be recorded. However, a specified time window will not be given to ease burden on patients and to maximise participation.

Table 2. PIMS trial study measures

Domain	Tool	Source	Validated Tool	Time of assessment
Sociodemographic/lifestyle	Screening questionnaire	Self-report		Screening
	Medical History Questionnaire	Self-report/General practice		Eligibility
	Substance Use Questionnaire	Self-report		Eligibility
	Physical Measurements Form	Self-report		Baseline
	Sociodemographic Questionnaire	Self-report		Baseline
Psychiatric	The Temporal Experience of Pleasure Scale	Self-report	✓	Eligibility, baseline, follow-ups
	The Positive and Negative Syndrome Scale	Interviewer assessed	✓	Eligibility, baseline, follow-ups
	The Mini-International Neuropsychiatric Interview	Interviewer assessed	✓	Eligibility
	Psychiatric History Questionnaire	Self-report		Baseline
	The Scale for the Assessment of Negative Symptoms	Self-report	✓	Baseline, follow-ups
	The Calgary Depression Scale for Schizophrenia	Interviewer assessed	✓	Baseline, follow-ups
	Multi-dimensional Fatigue Inventory	Self-report	✓	Baseline, follow-ups
	European Quality of Life-5 Dimensions Three-Level Version	Self-report	✓	Baseline, follow-ups
	Visual Analogue Scale for Subjective Wellbeing	Self-report	✓	Baseline, follow-ups
Cognitive	National Adult Reading Test for estimated premorbid IQ	Interviewer assessed	✓	Baseline, follow-up 2

For peer review only

For peer review only - http://bmjopen.bmj.com/site/about/guidelines.xhtml

	CANTAB Reaction Time test	Computer task	✓	Baseline, follow-up 2
	Symbol Coding Test	Paper task	✓	Baseline, follow-up 2
	CANTAB Rapid Visual Information Processing test	Computer task	✓	Baseline, follow-up 2
	CANTAB Paired Associates Learning test	Computer task	✓	Baseline, follow-up 2
	CANTAB One Touch Stockings of Cambridge test	Computer task	✓	Baseline, follow-up 2
Biologic	Inflammatory markers, cardiometabolic markers, IDO activation, white cell phenotyping	Laboratory tests		Baseline, follow-ups
Genetic	Gene expression/genotyping	Blood (RNA, DNA)		Baseline, follow-ups
Neuroimaging	MRI Screening Questionnaire			Baseline, follow-up 2
	Structural MRI, 1H-MRS measure of glutathione in the prefrontal cortex area, resting state fMRI			Baseline, follow-up 2

For peer review only

Sample size and statistical power

We will recruit approximately 60 patients with psychosis. However, currently there are no trials of immunotherapies for anhedonia in schizophrenia making accurate power calculation difficult. This study is a proof-of-concept experiment designed to test whether inhibition of IL-6 signalling leads to changes in psychotic symptoms. It could also inform likely statistical power for future trials testing efficacy of the drug as a treatment of schizophrenia, which is not the intention of this study. The exact statistical tests and techniques that will be applied to the data will depend on the objective of specific analysis and data characteristics (e.g.,

For peer review only - http://bmjopen.bmj.com/site/about/guidelines.xhtml

variable type, distribution). These details will be specified in analysis plans and registered online before participants are unblinded and any data analysis is performed.

Randomisation and blinding

An external agency independent of the study team will arrange random allocation to tocilizumab or normal saline group 1:1, ensuring two groups are comparable regarding anhedonia severity and sex. Randomisation will be stratified by site. Randomising agency will provide the randomisation code to the relevant hospital pharmacy who will dispense tocilizumab or normal saline according to the randomisation schedule. Dispensing pharmacies will keep a log of products dispensed. Infusions will be prepared and administered at clinical research facilities (CRFs). Infusion packs will be prepared by trained staff not part of the core study team, ensuring blinding of treatment allocation. Infusion packs containing drug or placebo will be visually indistinguishable from each other, ensuring that both participants and study team remain blind regarding treatment allocation.

For peer review only

Statistical analysis

For randomised participants, an intention-to-treat approach will be taken for data analysis by including all randomised participants in statistical analyses, regardless of the treatment they received (if any). We will compare outcome measures between treatment and placebo groups controlling for baseline scores. This mechanistic experiment will focus on overall pattern of results and their effect sizes rather than *P*-values for individual tests of statistical significance. The secondary mechanistic and observational analysis will compare psychotic symptoms, cognitive function, blood, neuroimaging, and other biomarkers between and across study groups using appropriate statistical tests.

STUDY PROCEDURE

An overview of study procedures is presented in Figure 1 and all study measures are detailed in Table 2. Recruitment will take place in Bristol, Birmingham, and Cambridge and assessments at University and NHS research facilities.

Participant identification

Potential participants with psychosis will be identified by NHS Psychosis Early Intervention (EI) teams. HCs will be recruited through advertisement methods in Birmingham and Cambridge. Potential participants will complete a screening questionnaire to confirm their eligibility to participate. If deemed eligible, participants will be invited to an appointment to complete a full eligibility assessment.

Eligibility assessment

Assessments will be carried out to establish eligibility and to obtain informed consent. Patients will complete the MINI to confirm ICD-10 diagnosis of schizophrenia and related psychoses, the PANSS to confirm the presence of positive symptoms of psychosis, and the TEPS to confirm eligibility based on anticipatory and consummatory pleasure sum scores. A blood sample will be collected from patients for serum IL-6 measurement. An MRI screening questionnaire will be administered to those willing to give informed consent for neuroimaging.

Baseline assessment

All participants (60 inflamed psychosis, 30 non-inflamed psychosis, and 30 HCs) will attend a baseline assessment comprising psychiatric measures, cognitive tasks, blood sampling, and neuroimaging (optional). This will be the final study contact for patients without evidence of

For peer review only

For peer review only - http://bmjopen.bmj.com/site/about/guidelines.xhtml

inflammation and HCs. Patients with evidence of inflammation will undergo further tests to establish safety/eligibility to receive tocilizumab, including a chest X-ray and blood tests to exclude pregnancy and certain infections, such as TB, HIV, and COVID-19. Eligible participants will be randomised and invited for infusion.

Intervention

Intravenous infusion of tocilizumab or normal saline will be given continuously over one hour at CRFs in Bristol, Birmingham, and Cambridge by trained clinical staff under the supervision of a designated study doctor. Participants will remain under clinical observation for a further 1-hour period after the end of infusion.

Follow-up assessments

Follow-up assessments will take place approximately 7-, 14-, and 28-days post-infusion, and will collect similar data to the baseline assessment. Cognitive tasks and neuroimaging (optional) will be administered only on day 14. Around 42 days post-infusion, participants will be contacted by phone to provide a final debrief; at which point they will exit the study.

RISK MANAGEMENT

Psychosis-related risks

All patients will be under the care of a specialist NHS psychosis EI service. Participation will not involve any treatment modifications or significant delays in receiving treatment. If a patient becomes distressed during an assessment, or does not wish to continue for any reason, the researcher will stop the assessment. Participants may withdraw at any time without giving a reason. If there is any concern for the participant’s safety, the research team will liaise with participant’s GP and/or mental health team as needed.

For peer review only

For peer review only - http://bmjopen.bmj.com/site/about/guidelines.xhtml

Procedure-related risks

Venepuncture

Blood taking is associated with mild discomfort and other side effects are rare. Efforts will be made to minimise discomfort. Blood taking will be performed by a nurse, doctor, or research team member trained in venepuncture.

Chest X-ray

This study will use a typical effective radiation dose of 0.014 mSv; equivalent to 2.5 days of average natural background radiation in the UK. The risk of developing cancer as a consequence of participating in this study is 0.0001%. Only non-pregnant, adult participants will be included.

Neuroimaging

Discomfort during MRI will be minimised by using mirrors to allow participants to view outside of the machine, providing ear plugs and a panic button, and allowing participants to communicate with the researcher and scan operator throughout. Mild transient vertigo may be experienced when being moved into the MRI machine. Risk of dislodgement or malfunction of medical implants or metallic foreign objects will be minimised by screening participants to ensure no metal is present on or within the body.

IL-6 levels

Some participants will show evidence of inflammation in the blood (IL-6 ≥0.7pg/ml). This is not necessarily a cause for concern. In people with FEP, ~50% have serum IL-6 levels >0.7pg/ml. Reasons for this in the absence of an acute infection or chronic inflammatory

For peer review only - http://bmjopen.bmj.com/site/about/guidelines.xhtml

For peer review only

illness could include obesity, smoking, alcohol use, and lack of exercise, so knowledge of ‘inflammation status’ may prompt participants to adopt a healthier lifestyle. If serum IL-6 level is high (i.e., IL-6 $\geq$0.7pg/ml) along with elevated CRP (>20mg/L) without any apparent explanation, such as infection or chronic inflammatory illness, we will inform the participant’s GP and the participant will be excluded from the study.

Risk to research staff

Staff will follow local safety procedures when lone working. No other risks are anticipated.

Safety considerations for infusion and monitoring of adverse reaction

Before infusion

Participants will be selected based on strict inclusion and exclusion criteria. Additionally, we will carry out tests for TB, HIV, VZV antibody, and Hepatitis B and C because, though unlikely after a single dose, tocilizumab could make these infections worse. Female participants of childbearing age will be given a pregnancy test, which must be negative. Participants who are sexually active will be asked to use at least one form of effective contraception for six weeks post-infusion. Male participants will also be asked not to donate sperm samples for six weeks post-infusion.

During infusion

Infusions will be given under supervision of a designated study doctor. Participants will be monitored for possible side effects, which will be managed in line with use of tocilizumab for treating patients with RA.

After infusion

For peer review only

Participants will remain under observation for one-hour post-infusion. Participants will be advised to seek help if they feel unwell after leaving the assessment centre and will be given an information sheet containing a telephone number their health professionals can call. If necessary, we will unblind the participant and inform their health professional whether they received tocilizumab or normal saline. Adverse reactions will be recorded at each follow-up visit. Additional, safety blood tests will be done at second follow-up (e.g., WBC count, liver function, lipids).

ETHICS AND DISSEMINATION

The study will be conducted in accordance with the REC, Health Research Authority (HRA), and local Research and Development (R&D) department approvals and guidelines (REC reference: 22/EE/0010). The study team will prepare protocol amendments as required and ethics approval will be sought before implementing any changes to the approved protocol. The ISRCTN Trial Registry and the Research Governance Office will be informed of any amendments to the protocol.

Consent

Informed consent will be obtained prior to eligibility assessments for participation in the study (Appendix I, II, and II). This will include consent to randomise, for contact with their GP to inform them about participation, access GP/psychiatric records to verify medical history to establish eligibility, and to inform the participant's GP any results/outcomes as necessary. Consent for additional tests to establish safety for tocilizumab infusion and for storing biological samples will also be obtained.

Study management

For peer review only - http://bmjopen.bmj.com/site/about/guidelines.xhtml

The study is sponsored by the University of Bristol. The sponsor, the Chief Investigator (GMK), and the co-Lead (RU) will have overall responsibility for the study. A named principal investigator will take clinical responsibility for study activities at each site. The study does not require the formal arrangement of a steering committee because, according to the HRA, it is not a Clinical Trial of an Investigational Medicinal Product. However, to enhance monitoring of the study, a study management group will be established, comprising academic and clinical experts in psychiatry, rheumatology, neuroscience, and immunology.

Data management and retention of samples

All potential participants will be assigned a unique study-specific participant ID number. All data will be subject to good practice as laid down in the Data Protection Act. Each study stage is tracked so that participant's (de-identified) status within the study is known, and assessment and other appointment dates are forecasted. This information is held on a secure, password-protected database. Anonymised data from assessments will be uploaded to a secure, password-protected database using secure web-based data entry systems. Minimal personal data (age, sex) will be indexed by each participant's unique ID number. Blood samples collected in this study may be stored for up to 10 years after the completion for additional research. Stored samples will be coded throughout the sample storage and analysis process and will not be labelled with personal identifiers. Participants may withdraw their consent for their samples to be stored for future research.

Dissemination plan

Study results will be published in peer-review journals and will conform to the guidelines of the International Committee of Medical Journal Editors. Findings will be disseminated at conferences, departmental talks, and via social and traditional media.

For peer review only

For peer review only - http://bmjopen.bmj.com/site/about/guidelines.xhtml

AUTHORS CONTRIBUTIONS

ÉMF wrote first draft of the PIMS trial protocol and of this manuscript. SLG, MK, GKM, BD, DJ, JS, and NMB contributed to study design and protocol development and revised manuscript drafts. RU contributed to study design and study protocol, and revised manuscript drafts. GMK devised study design and trial protocol, and revised drafts. ÉMF and SLG developed study materials and liaised with REC and HRA regarding approvals. AM, JR, FCZ, HH, EW, and MW contributed to the revision of the manuscript and validation of operating procedures and mechanistic protocols. RU and GMK co-lead the MRC grant that funds the PIMS trial and provide overall supervision and oversight for the project.

FUNDING STATEMENT

The PIMS trial is funded by a Medical Research Council (MRC) grant to RU and GMK; Grant Ref: MR/S037675/1. ÉMF is supported by an MRC Integrative Epidemiology Unit PhD Studentship. AM is supported by funding from the MRC for doctoral training (MR/2434208). FCZ receives a PhD Fellowship from the São Paulo Research Foundation (2019/13229-2 and 2021/07448-3). HH is supported by the PIMS Trial MRC grant (MR/S037675/1). DJ is supported by the Cambridge Arthritis Research Endeavour and the National Institute for Health Research (NIHR) Cambridge Biomedical Research Centre (BRC-1215-20014). NMB acknowledges funding support from the MRC (MR/R006008/1 and MR/N019016/1), Ministry of Defence (702931454), Diabetes UK (20/0006296), NIHR (14/WM/0093), and Innovate UK (84361). RU has grants from MRC, NIHR: Health Technology Assessment, European Commission - Research: The Seventh Framework Programme, and personal fees from Sunovion, outside the submitted work. GMK

For peer review only

For peer review only - http://bmjopen.bmj.com/site/about/guidelines.xhtml

acknowledges funding support from the Wellcome Trust (Grant No. 201486/Z/16/Z). The funders had no role in the design of this study.

COMPETING INTERESTS STATEMENT

ÉMF, SLG, AM, JR, FCZ, HH, EW, MW, MK, GKM, BD, DJ, JS, RU, and GMK have no conflicts of interest to report. NMB holds shares and is a Director of Celentyx Ltd.

For peer review only

For peer review only - http://bmjopen.bmj.com/site/about/guidelines.xhtml

REFERENCES

1 Khandaker GM, Cousins L, Deakin J, *et al.* Inflammation and immunity in schizophrenia: implications for pathophysiology and treatment. *Lancet Psychiatry* 2015;**2**:258–70. doi:10.1016/S2215-0366(14)00122-9

2 Khandaker GM, Dantzer R. Is there a role for immune-to-brain communication in schizophrenia? *Psychopharmacology* 2016;**233**:1559–73. doi:10.1007/s00213-015-3975-1

3 Khandaker GM, Dantzer R, Jones PB. Immunopsychiatry: important facts. *Psychol Med* 2017;**47**:2229–37. doi:10.1017/S0033291717000745

4 Upthegrove R, Khandaker G. Cytokines, Oxidative Stress and Cellular Markers of Inflammation in Schizophrenia. *Current topics in behavioral neurosciences* 2020;**44**:49–66. doi:10.1007/7854_2018_88

5 Upthegrove R, Manzanares-Teson N, Barnes NM. Cytokine function in medication-naive first episode psychosis: a systematic review and meta-analysis. *Schizophr Res* 2014;**155**:101–8. doi:10.1016/j.schres.2014.03.005

6 Goldsmith DR, Rapaport MH, Miller BJ. A meta-analysis of blood cytokine network alterations in psychiatric patients: comparisons between schizophrenia, bipolar disorder and depression. *Mol Psychiatry* 2016;**21**:1696–709. doi:10.1038/mp.2016.3

7 Miller BJ, Buckley P, Seabolt W, *et al.* Meta-analysis of cytokine alterations in schizophrenia: clinical status and antipsychotic effects. *Biological psychiatry* 2011;**70**:663–71. doi:10.1016/j.biopsych.2011.04.013

8 Potvin S, Stip E, Sepehry AA, *et al.* Inflammatory cytokine alterations in schizophrenia: a systematic quantitative review. *Biol Psychiatry* 2008;**63**:801–8. doi:10.1016/j.biopsych.2007.09.024

9 Wang AK, Miller BJ. Meta-analysis of Cerebrospinal Fluid Cytokine and Tryptophan Catabolite Alterations in Psychiatric Patients: Comparisons Between Schizophrenia, Bipolar Disorder, and Depression. *Schizophr Bull* 2018;**44**:75–83. doi:10.1093/schbul/sbx035

10 Khandaker GM, Zimbron J, Dalman C, *et al.* Childhood infection and adult schizophrenia: a meta-analysis of population-based studies. *Schizophr Res* 2012;**139**:161–8. doi:10.1016/j.schres.2012.05.023

11 Khandaker GM, Zimbron J, Lewis G, *et al.* Prenatal maternal infection, neurodevelopment and adult schizophrenia: a systematic review of population-based studies. *Psychol Med* 2013;**43**:239–57. doi:10.1017/S0033291712000736

12 Khandaker GM, Zammit S, Burgess S, *et al.* Association between a functional interleukin 6 receptor genetic variant and risk of depression and psychosis in a population-based birth cohort. *Brain Behav Immun* 2018;**69**:264–72. doi:10.1016/j.bbi.2017.11.020

13 Perry BI, Upthegrove R, Kappelmann N, *et al.* Associations of immunological proteins/traits with schizophrenia, major depression and bipolar disorder: A bi-directional

For peer review only - http://bmjopen.bmj.com/site/about/guidelines.xhtml

two-sample mendelian randomization study. *Brain, Behavior, and Immunity* 2021;**97**:176–85. doi:10.1016/j.bbi.2021.07.009

14 Williams JA, Burgess S, Suckling J, *et al.* Inflammation and Brain Structure in Schizophrenia and Other Neuropsychiatric Disorders: A Mendelian Randomization Study. *JAMA Psychiatry* 2022;**79**:498. doi:10.1001/jamapsychiatry.2022.0407

15 Kose M, Pariante CM, Dazzan P, *et al.* The Role of Peripheral Inflammation in Clinical Outcome and Brain Imaging Abnormalities in Psychosis: A Systematic Review. *Front Psychiatry* 2021;**12**:612471. doi:10.3389/fpsyt.2021.612471

16 Lesh TA, Careaga M, Rose DR, *et al.* Cytokine alterations in first-episode schizophrenia and bipolar disorder: relationships to brain structure and symptoms. *Journal of Neuroinflammation* 2018;**15**:165. doi:10.1186/s12974-018-1197-2

17 Trépanier MO, Hopperton KE, Mizrahi R, *et al.* Postmortem evidence of cerebral inflammation in schizophrenia: a systematic review. *Mol Psychiatry* 2016;**21**:1009–26. doi:10.1038/mp.2016.90

18 van Kesteren CFMG, Gremmels H, de Witte LD, *et al.* Immune involvement in the pathogenesis of schizophrenia: a meta-analysis on postmortem brain studies. *Transl Psychiatry* 2017;**7**:e1075–e1075. doi:10.1038/tp.2017.4

19 Marques TR, Ashok AH, Pillinger T, *et al.* Neuroinflammation in schizophrenia: meta-analysis of in vivo microglial imaging studies. *Psychological Medicine* 2019;**49**:2186–96. doi:10.1017/S0033291718003057

20 Murray AJ, Rogers JC, Katshu MZUH, *et al.* Oxidative Stress and the Pathophysiology and Symptom Profile of Schizophrenia Spectrum Disorders. *Frontiers in Psychiatry* 2021;**12**.https://www.frontiersin.org/articles/10.3389/fpsyt.2021.703452 (accessed 9 Aug 2022).

21 Upthegrove R. The immune system and schizophrenia: An update for clinicians. *Advances in Psychiatric Treatment* 2014;**20**:83–91. doi:10.1192/apt.bp.113.011452

22 Conen S, Gregory CJ, Hinz R, *et al.* Neuroinflammation as measured by positron emission tomography in patients with recent onset and established schizophrenia: implications for immune pathogenesis. *Mol Psychiatry* 2021;**26**:5398–406. doi:10.1038/s41380-020-0829-y

23 van Mierlo HC, Schot A, Boks MPM, *et al.* The association between schizophrenia and the immune system: Review of the evidence from unbiased 'omic-studies.' *Schizophrenia Research* 2020;**217**:114–23. doi:10.1016/j.schres.2019.05.028

24 Plavén-Sigray P, Matheson GJ, Collste K, *et al.* Positron Emission Tomography Studies of the Glial Cell Marker Translocator Protein in Patients With Psychosis: A Meta-analysis Using Individual Participant Data. *Biological Psychiatry* 2018;**84**:433–42. doi:10.1016/j.biopsych.2018.02.1171

25 Plavén-Sigray P, Matheson GJ, Coughlin JM, *et al.* Meta-analysis of the Glial Marker TSPO in Psychosis Revisited: Reconciling Inconclusive Findings of Patient-Control Differences. *Biol Psychiatry* 2021;**89**:e5–8. doi:10.1016/j.biopsych.2020.05.028

For peer review only

26 Deakin B, Suckling J, Barnes TRE, *et al.* The benefit of minocycline on negative symptoms of schizophrenia in patients with recent-onset psychosis (BeneMin): a randomised, double-blind, placebo-controlled trial. *The Lancet Psychiatry* 2018;**5**:885–94. doi:10.1016/S2215-0366(18)30345-6

27 Weiser M, Levi L, Burshtein S, *et al.* The effect of minocycline on symptoms in schizophrenia: Results from a randomized controlled trial. *Schizophrenia Research* 2019;**206**:325–32. doi:10.1016/j.schres.2018.10.023

28 Ramaker RC, Bowling KM, Lasseigne BN, *et al.* Post-mortem molecular profiling of three psychiatric disorders. *Genome Medicine* 2017;**9**:72. doi:10.1186/s13073-017-0458-5

29 González-Peñas J, Costas J, Villamayor MJG, *et al.* Enrichment of rare genetic variants in astrocyte gene enriched co-expression modules altered in postmortem brain samples of schizophrenia. *Neurobiology of Disease* 2019;**121**:305–14. doi:10.1016/j.nbd.2018.10.013

30 Gandal MJ, Zhang P, Hadjimichael E, *et al.* Transcriptome-wide isoform-level dysregulation in ASD, schizophrenia, and bipolar disorder. *Science* 2018;**362**:eaat8127. doi:10.1126/science.aat8127

31 Gandal MJ, Haney JR, Parikshak NN, *et al.* Shared molecular neuropathology across major psychiatric disorders parallels polygenic overlap. *Science* 2018;**359**:693–7. doi:10.1126/science.aad6469

32 Toker L, Mancarci BO, Tripathy S, *et al.* Transcriptomic Evidence for Alterations in Astrocytes and Parvalbumin Interneurons in Subjects With Bipolar Disorder and Schizophrenia. *Biological Psychiatry* 2018;**84**:787–96. doi:10.1016/j.biopsych.2018.07.010

33 Corsi-Zuelli F, Deakin B. Impaired regulatory T cell control of astroglial overdrive and microglial pruning in schizophrenia. *Neuroscience & Biobehavioral Reviews* 2021;**125**:637–53. doi:10.1016/j.neubiorev.2021.03.004

34 Corsi-Zuelli F, Deakin B, de Lima MHF, *et al.* T regulatory cells as a potential therapeutic target in psychosis? Current challenges and future perspectives. *Brain, Behavior, & Immunity - Health* 2021;**17**:100330. doi:10.1016/j.bbih.2021.100330

35 Jeppesen R, Christensen RHB, Pedersen EMJ, *et al.* Efficacy and safety of anti-inflammatory agents in treatment of psychotic disorders – A comprehensive systematic review and meta-analysis. *Brain, Behavior, and Immunity* 2020;**90**:364–80. doi:10.1016/j.bbi.2020.08.028

36 Girgis RR, Ciarleglio A, Choo T, *et al.* A Randomized, Double-Blind, Placebo-Controlled Clinical Trial of Tocilizumab, An Interleukin-6 Receptor Antibody, For Residual Symptoms in Schizophrenia. *Neuropsychopharmacology : official publication of the American College of Neuropsychopharmacology* 2018;**43**:1317–23. doi:10.1038/npp.2017.258

For peer review only - http://bmjopen.bmj.com/site/about/guidelines.xhtml

37 Mondelli V, Ciufolini S, Belvederi Murri M, *et al.* Cortisol and Inflammatory Biomarkers Predict Poor Treatment Response in First Episode Psychosis. *Schizophrenia bulletin* 2015;**41**:1162–70. doi:10.1093/schbul/sbv028

38 Miller BJ, Culpepper N, Rapaport MH. C-reactive protein levels in schizophrenia: a review and meta-analysis. *Clinical schizophrenia & related psychoses* 2014;**7**:223–30. doi:10.3371/CSRP.MICU.020813

39 Raison CL, Rutherford RE, Woolwine BJ, *et al.* A randomized controlled trial of the tumor necrosis factor antagonist infliximab for treatment-resistant depression: The role of baseline inflammatory biomarkers. *JAMA Psychiatry* 2013;**70**:31–41. doi:10.1001/2013.jamapsychiatry.4

40 Dunleavy C, Elsworthy RJ, Upthegrove R, *et al.* Inflammation in first-episode psychosis: The contribution of inflammatory biomarkers to the emergence of negative symptoms, a systematic review and meta-analysis. *Acta Psychiatrica Scandinavica* 2022;**146**:6–20. doi:10.1111/acps.13416

41 Khandaker GM, Stochl J, Zammit S, *et al.* Association between circulating levels of C-reactive protein and positive and negative symptoms of psychosis in adolescents in a general population birth cohort. *J Psychiatr Res* 2021;**143**:534–42. doi:10.1016/j.jpsychires.2020.11.028

42 Edwards CJ, Cella M, Tarrier N, *et al.* Investigating the empirical support for therapeutic targets proposed by the temporal experience of pleasure model in schizophrenia: A systematic review. *Schizophrenia research* 2015;**168**:120–44. doi:10.1016/j.schres.2015.08.013

43 Lalousis PA, Schmaal L, Wood SJ, *et al.* Neurobiologically Based Stratification of Recent-Onset Depression and Psychosis: Identification of Two Distinct Transdiagnostic Phenotypes. *Biological Psychiatry* 2022;:S0006322322011568. doi:10.1016/j.biopsych.2022.03.021

44 Fioravanti M, Carlone O, Vitale B, *et al.* A Meta-Analysis of Cognitive Deficits in Adults with a Diagnosis of Schizophrenia. *Neuropsychol Rev* 2005;**15**:73–95. doi:10.1007/s11065-005-6254-9

45 Keefe RSE, Bilder RM, Davis SM, *et al.* Neurocognitive Effects of Antipsychotic Medications in Patients With Chronic Schizophrenia in the CATIE Trial. *Archives of General Psychiatry* 2007;**64**:633–47. doi:10.1001/archpsyc.64.6.633

46 Chan A-W, Tetzlaff JM, Altman DG, *et al.* SPIRIT 2013 Statement: defining standard protocol items for clinical trials. *Rev Panam Salud Publica* 2015;**38**:506–14.

47 Calabrese LH, Rose-John S. IL-6 biology: implications for clinical targeting in rheumatic disease. *Nature reviews Rheumatology* 2014;**10**:720–7. doi:10.1038/nrrheum.2014.127

48 Choy EHS, Isenberg DA, Garrood T, *et al.* Therapeutic benefit of blocking interleukin-6 activity with an anti-interleukin-6 receptor monoclonal antibody in rheumatoid arthritis: a randomized, double-blind, placebo-controlled, dose-escalation trial. *Arthritis and rheumatism* 2002;**46**:3143–50. doi:10.1002/art.10623

For peer review only - http://bmjopen.bmj.com/site/about/guidelines.xhtml

49 Woo P, Wilkinson N, Prieur A-M, *et al.* Open label phase II trial of single, ascending doses of MRA in Caucasian children with severe systemic juvenile idiopathic arthritis: proof of principle of the efficacy of IL-6 receptor blockade in this type of arthritis and demonstration of prolonged clinical improvement. *Arthritis research & therapy* 2005;**7**:R1281-8. doi:10.1186/ar1826

50 Gard DE, Gard MG, Kring AM, *et al.* Anticipatory and consummatory components of the experience of pleasure: A scale development study. *Journal of Research in Personality* 2006;**40**:1086–102. doi:10.1016/j.jrp.2005.11.001

For peer review only

FIGURE LEGEND

Figure 1. Overview of study design

For peer review only

For peer review only - http://bmjopen.bmj.com/site/about/guidelines.xhtml

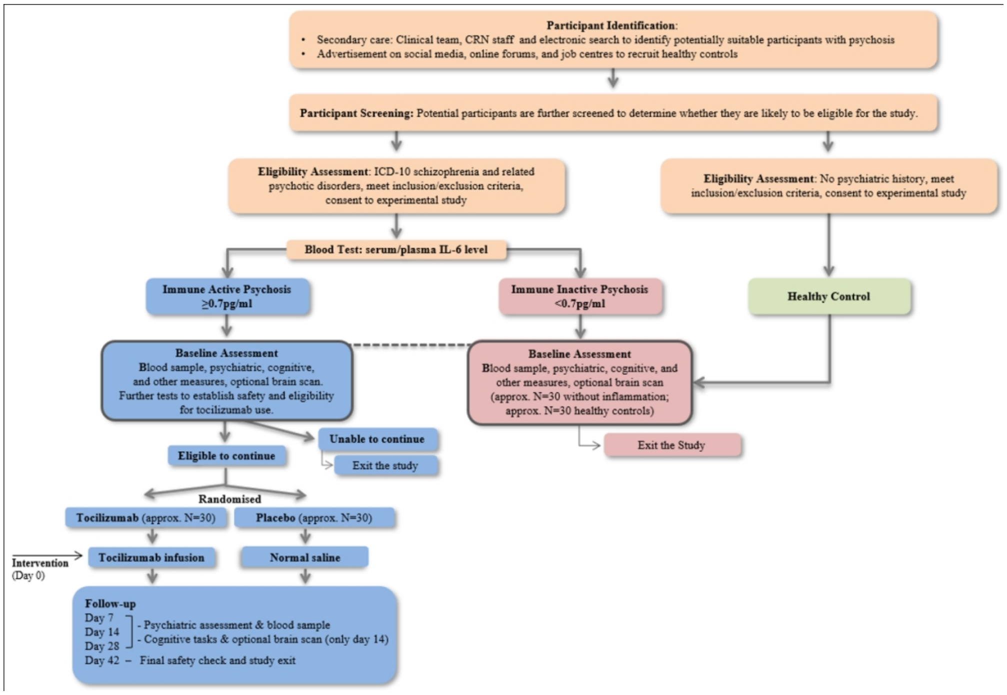

Figure 1. Overview of study design

240x165mm (144 x 144 DPI)

Supplementary Material

Foley et al. Protocol for the Psychosis Immune Mechanism Stratified Medicine (PIMS) trial: A randomised double-blind placebo-controlled trial of single dose tocilizumab in patients with psychosis.

Table of Contents

eTable1: SPIRIT 2013 Checklist 2

Appendix I – Consent Form for Screening: All Participants 8

Appendix II – Informed Consent Form for Study Participation: Patients 10

Appendix III – Informed Consent Form for Study Participation: Healthy Controls........ 13

For peer review only

eTable1: SPIRIT 2013 Checklist – Recommended items to address in a clinical trial protocol and related documents*

SPIRIT

STANDARD PROTOCOL ITEMS: RECOMMENDATIONS FOR INTERVENTIONAL TRIALS

Section/item	Item No	Description	Addressed on page number
**Administrative information**			
Title	1	Descriptive title identifying the study design, population, interventions, and, if applicable, trial acronym	1
Trial registration	2a	Trial identifier and registry name. If not yet registered, name of intended registry	4
	2b	All items from the World Health Organization Trial Registration Data Set	4 (ISRCTN 23256704)
Protocol version	3	Date and version identifier	-
Funding	4	Sources and types of financial, material, and other support	24 - 25
Roles and responsibilities	5a	Names, affiliations, and roles of protocol contributors	1, 24
	5b	Name and contact information for the trial sponsor	23
	5c	Role of study sponsor and funders, if any, in study design; collection, management, analysis, and interpretation of data; writing of the report; and the decision to submit the report for publication, including whether they will have ultimate authority over any of these activities	23-25

For peer review only

For peer review only - http://bmjopen.bmj.com/site/about/guidelines.xhtml

	5d	Composition, roles, and responsibilities of the coordinating centre, steering committee, endpoint adjudication committee, data management team, and other individuals or groups overseeing the trial, if applicable (see Item 21a for data monitoring committee)	23 - 25
**Introduction**			
Background and rationale	6a	Description of research question and justification for undertaking the trial, including summary of relevant studies (published and unpublished) examining benefits and harms for each intervention	6-9
	6b	Explanation for choice of comparators	6-9
Objectives	7	Specific objectives or hypotheses	9-10
Trial design	8	Description of trial design including type of trial (eg, parallel group, crossover, factorial, single group), allocation ratio, and framework (eg, superiority, equivalence, noninferiority, exploratory)	9
**Methods: Participants, interventions, and outcomes**			
Study setting	9	Description of study settings (e.g., community clinic, academic hospital) and list of countries where data will be collected. Reference to where list of study sites can be obtained	9, 11
Eligibility criteria	10	Inclusion and exclusion criteria for participants. If applicable, eligibility criteria for study centres and individuals who will perform the interventions (eg, surgeons, psychotherapists)	12, Table 1
Interventions	11a	Interventions for each group with sufficient detail to allow replication, including how and when they will be administered	11, 19, 21-22, Figure 1
	11b	Criteria for discontinuing or modifying allocated interventions for a given trial participant (eg, drug dose change in response to harms, participant request, or improving/worsening disease)	22
	11c	Strategies to improve adherence to intervention protocols, and any procedures for monitoring adherence (eg, drug tablet return, laboratory tests)	-
	11d	Relevant concomitant care and interventions that are permitted or prohibited during the trial	12, Table 1

For peer review only - http://bmjopen.bmj.com/site/about/guidelines.xhtml

Outcomes	12	Primary, secondary, and other outcomes, including the specific measurement variable (eg, systolic blood pressure), analysis metric (eg, change from baseline, final value, time to event), method of aggregation (eg, median, proportion), and time point for each outcome. Explanation of the clinical relevance of chosen efficacy and harm outcomes is strongly recommended	9-10, Figure 1, Table 2
Participant timeline	13	Time schedule of enrolment, interventions (including any run-ins and washouts), assessments, and visits for participants. A schematic diagram is highly recommended (see Figure)	18-19, Figure 1
Sample size	14	Estimated number of participants needed to achieve study objectives and how it was determined, including clinical and statistical assumptions supporting any sample size calculations	17
Recruitment	15	Strategies for achieving adequate participant enrolment to reach target sample size	18
**Methods: Assignment of interventions (for controlled trials)**			
Allocation:			
Sequence generation	16a	Method of generating the allocation sequence (eg, computer-generated random numbers), and list of any factors for stratification. To reduce predictability of a random sequence, details of any planned restriction (eg, blocking) should be provided in a separate document that is unavailable to those who enrol participants or assign interventions	17
Allocation concealment mechanism	16b	Mechanism of implementing the allocation sequence (eg, central telephone; sequentially numbered, opaque, sealed envelopes), describing any steps to conceal the sequence until interventions are assigned	17
Implementation	16c	Who will generate the allocation sequence, who will enrol participants, and who will assign participants to interventions	17
Blinding (masking)	17a	Who will be blinded after assignment to interventions (eg, trial participants, care providers, outcome assessors, data analysts), and how	17
	17b	If blinded, circumstances under which unblinding is permissible, and procedure for revealing a participant's allocated intervention during the trial	22

**Methods: Data collection, management, and analysis**			
Data collection methods	18a	Plans for assessment and collection of outcome, baseline, and other trial data, including any related processes to promote data quality (eg, duplicate measurements, training of assessors) and a description of study instruments (eg, questionnaires, laboratory tests) along with their reliability and validity, if known. Reference to where data collection forms can be found, if not in the protocol	10-15, 18-19, Table 2
	18b	Plans to promote participant retention and complete follow-up, including list of any outcome data to be collected for participants who discontinue or deviate from intervention protocols	17-18
Data management	19	Plans for data entry, coding, security, and storage, including any related processes to promote data quality (eg, double data entry; range checks for data values). Reference to where details of data management procedures can be found, if not in the protocol	23-24
Statistical methods	20a	Statistical methods for analysing primary and secondary outcomes. Reference to where other details of the statistical analysis plan can be found, if not in the protocol	17-18
	20b	Methods for any additional analyses (eg, subgroup and adjusted analyses)	17-18
	20c	Definition of analysis population relating to protocol non-adherence (eg, as randomised analysis), and any statistical methods to handle missing data (eg, multiple imputation)	-
**Methods: Monitoring**			
Data monitoring	21a	Composition of data monitoring committee (DMC); summary of its role and reporting structure; statement of whether it is independent from the sponsor and competing interests; and reference to where further details about its charter can be found, if not in the protocol. Alternatively, an explanation of why a DMC is not needed	23
	21b	Description of any interim analyses and stopping guidelines, including who will have access to these interim results and make the final decision to terminate the trial	-
Harms	22	Plans for collecting, assessing, reporting, and managing solicited and spontaneously reported adverse events and other unintended effects of trial interventions or trial conduct	20-22

Auditing	23	Frequency and procedures for auditing trial conduct, if any, and whether the process will be independent from investigators and the sponsor	-
**Ethics and dissemination**			
Research ethics approval	24	Plans for seeking research ethics committee/institutional review board (REC/IRB) approval	22
Protocol amendments	25	Plans for communicating important protocol modifications (eg, changes to eligibility criteria, outcomes, analyses) to relevant parties (eg, investigators, REC/IRBs, trial participants, trial registries, journals, regulators)	22
Consent or assent	26a	Who will obtain informed consent or assent from potential trial participants or authorised surrogates, and how (see Item 32)	22-23
	26b	Additional consent provisions for collection and use of participant data and biological specimens in ancillary studies, if applicable	22-23
Confidentiality	27	How personal information about potential and enrolled participants will be collected, shared, and maintained in order to protect confidentiality before, during, and after the trial	23-24
Declaration of interests	28	Financial and other competing interests for principal investigators for the overall trial and each study site	25
Access to data	29	Statement of who will have access to the final trial dataset, and disclosure of contractual agreements that limit such access for investigators	23
Ancillary and post-trial care	30	Provisions, if any, for ancillary and post-trial care, and for compensation to those who suffer harm from trial participation	22
Dissemination policy	31a	Plans for investigators and sponsor to communicate trial results to participants, healthcare professionals, the public, and other relevant groups (eg, via publication, reporting in results databases, or other data sharing arrangements), including any publication restrictions	24
	31b	Authorship eligibility guidelines and any intended use of professional writers	24
	31c	Plans, if any, for granting public access to the full protocol, participant-level dataset, and statistical code	-

For peer review

**Appendices**			
Informed consent materials	32	Model consent form and other related documentation given to participants and authorised surrogates	Appendix I, II and III
Biological specimens	33	Plans for collection, laboratory evaluation, and storage of biological specimens for genetic or molecular analysis in the current trial and for future use in ancillary studies, if applicable	23-24

*It is strongly recommended that this checklist be read in conjunction with the SPIRIT 2013 Explanation & Elaboration for important clarification on the items. Amendments to the protocol should be tracked and dated. The SPIRIT checklist is copyrighted by the SPIRIT Group under the Creative Commons "Attribution-NonCommercial-NoDerivs 3.0 Unported" license.

For peer review only

Appendix I – Consent Form for Screening: All Participants

Informed Consent Form for Screening
REC No. 22/EE/0010, Date: 08.02.2022, Version: 1.2

Psychosis Immune Mechanism Stratified Medicine Trial: The PIMS Trial

PARTICIPANT ID: ________

Thank you for considering taking part in the PIMS Trial. The research team must explain the eligibility assessment of the study to you before you agree to take part. If you have any questions arising from the Participant Information Sheet or from the explanation already given to you, please ask a member of the research team before you decide to participate. You will be given a copy of this Informed Consent Form to keep for future reference.

Please read the statements below and insert your initial in the box next to each statement if you agree with them:

Statement	Initial Here	
1. I have read and understood the information sheet version XX, DD.MM.YYYY and have had the opportunity to ask questions.		
2. I understand that my participation is voluntary and I am free to withdraw at any time without giving a reason, without my current or future medical care or legal rights being affected.		
3. I agree to provide blood samples for eligibility screening. I understand my blood samples will be analysed to test for evidence of immune activation. The purposes and possible risks of donating these samples have been explained to me. I understand that donated samples will be considered a gift but I will have the right to withdraw permission for analysis.		
4. I agree that my GP can be told that I am participating in the eligibility assessment of the PIMS Trial, and can be informed if any unexpected results are found pertaining specifically to my health.		
5. I consent for my GP/Psychiatrist to share information from my medical record in order to confirm my eligibility to take part in this study. The study team may access my GP/Psychiatrist records if necessary.		
Optional (Not agreeing to these will not exclude you from this study). Please tick Yes / No (as appropriate)	Yes	No
6. I agree that the samples can be stored after completion of the screening analysis, for use in future, ethically approved, non-genetic studies, even if I am deemed non-eligible to partake in the PIMS study.		
7. I agree that data and samples can be stored after completion of the PIMS Trial for use in future, ethically approved, genetic studies. This includes the main stocks of any genetic material collected, such as DNA and RNA.		

If you want to participate in the screening session of the PIMS Trial, please sign your name below:

Participant Signature ______________________

Participant Full Name _____________________

For peer review only - http://bmjopen.bmj.com/site/about/guidelines.xhtml

Date ____/ ____/ _______

The researcher who has explained this study to you also needs to sign this form:

Staff Signature ______________________

Staff Full Name _____________________

Date ____/ ____/ _______

Thank you for your help.

By completing and returning this form, you are giving us your consent that the personal information you provide will be treated as strictly confidential and handled in accordance with the provisions of the UK Data Protection Act 2018.

**When completed: 1 for participant; and 1 for researcher site file.*

For peer review only

Appendix II – Informed Consent Form for Study Participation: Patients

Informed Consent Form for Study Participation

REC No. 22/EE/0010, Date: 24.08.2022, Version: 1.5

Psychosis Immune Mechanism Stratified Medicine Trial: The PIMS Trial

PARTICIPANT ID: ________

Thank you for taking part in the PIMS Trial eligibility assessment. Based on this assessment, you are eligible to take part in the PIMS Trial. Before you agree to take part, the research team must explain the study to you. If you have any questions arising from the Participant Information Sheet or from the explanation already given to you, please ask a member of the research team before you decide to participate. You will be given a copy of this Informed Consent Form to keep for future reference.

Please read the statements below and insert your initial in the box next to each statement if you agree with them:

Statement	Initial Here
1. I have read and understood the information sheet version XX dated DD.MM.YYYY and have had the opportunity to ask questions.	
2. I agree to take part in the PIMS Trial. I understand that my participation is voluntary and I am free to withdraw at any time without giving a reason, without my current or future medical care or legal rights being affected.	
3. I understand that confidentiality and anonymity will be maintained and it will not be possible to identify me in any publications.	
4. I agree to partake in interviews, complete questionnaires, and cognitive tests as part of this study. I understand what will happen during the study assessments.	
5. I agree to provide blood samples. The purposes and possible risks of donating these samples have been explained to me. I understand that donated samples will be considered a gift but I will have the right to withdraw permission for analysis.	
6. I understand that blood samples collected from me will be used to measure non-genetic factors such as biochemical changes in the blood.	
7. I agree that the samples and information I provide can be stored, used and shared between PIMS Trial sites and with collaborators/contractors for the purpose of the study.	
8. I understand that blood samples collected will be stored at PIMS Trial centres.	
9. I understand that any of my samples (labelled with an anonymous ID only), or any information obtained from them, including the sequence of my genetic material, may be sent to specialist research laboratories in the UK and abroad for analyses and the results returned to PIMS Trial centres. Researchers at these laboratories have no access to personal information about study participants.	
10. I agree, if necessary, to provide blood/urine samples to test for pregnancy, COVID-19 immunity, Hepatitis B, Hepatitis C, HIV, VZV and Tuberculosis, and to undergo a chest X ray.	
11. I agree to being randomised into the tocilizumab or placebo group if deemed eligible to take part.	

For peer review only

12. I understand that taking part will involve the administration of a single intravenous infusion of the anti-inflammatory drug tocilizumab or normal saline.		
13. I agree that my GP can be told that I am participating in this study, and about any findings that require further attention.		
14. I understand that information related to my participation in this study may be accessed by responsible individuals from the sponsors of this study for quality control purposes. I give permission for these individuals to have access to this data.		
15. I understand that relevant sections of my medical notes and data collected during the study, may be looked at by individuals from research team, from regulatory authorities or from the NHS Trust, where it is relevant to my taking part in this research. I give permission for these individuals to have access to my records.		
Optional (Not agreeing to these will not exclude you from this study). Please tick Yes / No (as appropriate)	Yes	No
16. I agree to undergo brain scans as part of the PIMS Trial.		
17. I agree to be contacted in future by researchers to participate in follow up studies to this project, or in future studies of a similar nature.		
18. I understand that researchers may use the blood samples for genetic analysis.		
19. I agree that the samples can be stored after completion of the PIMS Trial for use in future, ethically approved, non-genetic studies.		
20. I agree that the information I give can be stored after completion of the PIMS Trial for use in future, ethically approved, non-genetic studies.		
21. I agree that data and samples can be stored after completion of the PIMS Trial for use in future, ethically approved, genetic studies. This includes the main stocks of any genetic material collected, such as DNA and RNA.		

If you want to participate in the PIMS Trial, please sign your name below:

Participant Signature ______________________

Participant Full Name _____________________

Date ____/ ____/ _______

Research staff who has explained this study to you also needs to sign this form:

Staff Signature ______________________

Staff Full Name _____________________

Date ____/ ____/ _______

For peer review only

Thank you for your help.

By completing and returning this form, you are giving us your consent that the personal information you provide will be treated as strictly confidential and handled in accordance with the provisions of the UK Data Protection Act 2018.

***When completed: 1 for participant; and 1 for researcher site file.*

For peer review only

Appendix III – Informed Consent Form for Study Participation: Healthy Controls

Healthy Controls Informed Consent Form for Study Participation

REC No. 22/EE/0010, Date: 17.02.2022, Version: 1

Psychosis Immune Mechanism Stratified Medicine Trial: The PIMS Trial

PARTICIPANT ID: ________

Thank you for taking part in the PIMS Trial eligibility assessment. Based on this assessment, you are eligible to take part in the PIMS Trial. Before you agree to take part, the research team must explain the study to you. If you have any questions arising from the Participant Information Sheet or from the explanation already given to you, please ask a member of the research team before you decide to participate. You will be given a copy of this Informed Consent Form to keep for future reference.

Please read the statements below and insert your initial in the box next to each statement if you agree with them:

**Statement**	**Initial Here**	
1. I have read and understood the information sheet version XX dated DD.MM.YYYY and have had the opportunity to ask questions.		
2. I agree to take part in the PIMS Trial. I understand that my participation is voluntary, and I am free to withdraw at any time without giving a reason, without my current or future medical care or legal rights being affected.		
3. I understand that confidentiality and anonymity will be maintained, and it will not be possible to identify me in any publications.		
4. I agree to partake in interviews, complete questionnaires, and cognitive tests as part of this study. I understand what will happen during the study assessments.		
5. I agree to provide blood samples. The purposes and possible risks of donating these samples have been explained to me. I understand that donated samples will be considered a gift, but I will have the right to withdraw permission for analysis.		
6. I understand that my blood samples collected will be stored at PIMS Trial centres.		
7. I understand that blood samples collected from me will be used to measure non-genetic factors such as biochemical changes in the blood.		
8. I understand that any of my samples (labelled with an anonymous ID only), or any information obtained from them, including the sequence of my genetic material, may be sent to specialist research laboratories in the UK and abroad for analyses and the results returned to PIMS Trial centres. Researchers at these laboratories have no access to personal information about study participants.		
9. I agree that the samples and information I provide can be stored, used and shared between PIMS Trial sites and with collaborators/contractors for the purpose of the study.		
10. I understand that information related to my participation in this study may be accessed by responsible individuals from the sponsors of this study for quality control purposes. I give permission for these individuals to have access to this data.		
Optional (Not agreeing to these will not exclude you from this study). Please tick Yes / No (as appropriate)	Yes	No

For peer review only - http://bmjopen.bmj.com/site/about/guidelines.xhtml

11. I agree to undergo brain scans as part of the PIMS Trial.		
12. I agree to be contacted in future by researchers to participate in follow up studies to this project, or in future studies of a similar nature.		
13. I understand that researchers may use the blood samples for genetic analysis.		
14. I agree that the samples can be stored after completion of the PIMS Trial for use in future, ethically approved, non-genetic studies.		
15. I agree that the information I give can be stored after completion of the PIMS Trial for use in future, ethically approved, non-genetic studies.		
16. I agree that data and samples can be stored after completion of the PIMS Trial for use in future, ethically approved, genetic studies. This includes the main stocks of any genetic material collected, such as DNA and RNA.		

If you want to participate in the PIMS Trial, please sign your name below:

Participant Signature ______________________

Participant Full Name _____________________

Date ____/ ____/ _______

Research staff who has explained this study to you also needs to sign this form:

Staff Signature ______________________

Staff Full Name _____________________

Date ____/ ____/ _______

For peer review only

Thank you for your help.

By completing and returning this form, you are giving us your consent that the personal information you provide will be treated as strictly confidential and handled in accordance with the provisions of the UK Data Protection Act 2018.

**When completed: 1 for participant; and 1 for researcher site file.*

For peer review only - http://bmjopen.bmj.com/site/about/guidelines.xhtml

BMJ Open

Protocol for the Psychosis Immune Mechanism Stratified Medicine (PIMS) trial: A randomised double-blind placebo-controlled trial of single dose tocilizumab in patients with psychosis

Journal:	*BMJ Open*
Manuscript ID	bmjopen-2022-067944.R2
Article Type:	Protocol
Date Submitted by the Author:	25-Jan-2023
Complete List of Authors:	Foley, Éimear; University of Bristol, MRC Integrative Epidemiology Unit, Population Health Sciences; Bristol Medical School, Centre for Academic Mental Health, Population Health Sciences<br>Griffiths, Sian Lowri; University of Birmingham, Institute for Mental Health and Centre for Human Brain Health<br>Murray, Alexander; University of Birmingham, Institute for Mental Health and Centre for Human Brain Health<br>Rogers, Jack; University of Birmingham, Institute for Mental Health and Centre for Human Brain Health<br>Corsi-Zuelli, Fabiana; University of Birmingham, Institute for Mental Health and Centre for Human Brain Health; University of Sao Paulo, Department of Neuroscience and Behaviour<br>Hickinbotham, Hannah; University of Cambridge, Department of Psychiatry<br>Warwick, Ella; University of Birmingham, Institute for Mental Health and Centre for Human Brain Health<br>Wilson, Martin; University of Birmingham, Institute for Mental Health and Centre for Human Brain Health<br>Kaser, Muzaffer; University of Cambridge, Psychiatry; Cambridgeshire and Peterborough NHS Foundation Trust<br>Murray, Graham K.; University of Cambridge, Psychiatry; Cambridgeshire and Peterborough NHS Foundation Trust<br>Deakin, Bill; The University of Manchester, Faculty of Medicine, Biology and Health<br>Jadon, Deepak; University of Cambridge, Medicine<br>Suckling, John; University of Cambridge, Psychiatry; Cambridgeshire and Peterborough NHS Foundation Trust<br>Barnes, Nicholas ; University of Birmingham, Institute of Clinical Sciences<br>Upthegrove, Rachel; University of Birmingham, Institute for Mental Health and Centre for Human Brain Health; Early Intervention Service, Birmingham Women's and Children's NHS Foundation Trust<br>Khandaker, Golam M.; University of Bristol, MRC Integrative Epidemiology Unit, Population Health Sciences; NIHR Bristol Biomedical Research Centre
**Primary Subject Heading**:	Mental health

Secondary Subject Heading:	Immunology (including allergy)
Keywords:	Schizophrenia & psychotic disorders < PSYCHIATRY, IMMUNOLOGY, Clinical trials < THERAPEUTICS, Magnetic resonance imaging < RADIOLOGY & IMAGING

SCHOLARONE™
Manuscripts

Protocol for the Psychosis Immune Mechanism Stratified Medicine (PIMS) trial: A randomised double-blind placebo-controlled trial of single dose tocilizumab in patients with psychosis

Éimear M. Foley[a,b]; Sian Lowri Griffiths[c]; Alexander Murray[c]; Jack Rogers[c]; Fabiana Corsi-Zuelli[c,d,e]; Hannah Hickinbotham[f]; Ella Warwick[c]; Martin Wilson[c]; Muzaffer Kaser[f,g]; Graham K. Murray[f,g]; Bill Deakin[h]; Deepak Jadon[i]; John Suckling[f,g]; Nicholas M. Barnes[e;] Rachel Upthegrove[c,j,†]; and Golam M. Khandaker[a,b,k,l,†,*]; for the PIMS Collaboration

[a] MRC Integrative Epidemiology Unit, Population Health Sciences, Bristol Medical School, University of Bristol, Bristol, United Kingdom

[b] Centre for Academic Mental Health, Population Health Sciences, Bristol Medical School, University of Bristol, Bristol, United Kingdom

[c] Institute for Mental Health and Centre for Human Brain Health, University of Birmingham, Birmingham, United Kingdom

[d] Department of Neuroscience and Behaviour, Division of Psychiatry, Ribeirão Preto Medical School, University of São Paulo, São Paulo, Brazil

[e] Institute of Clinical Sciences, College of Medical and Dental Sciences, University of Birmingham, United Kingdom

[f] Department of Psychiatry, University of Cambridge, United Kingdom

[g] Cambridgeshire and Peterborough NHS Foundation Trust, United Kingdom

[h] Faculty of Biology, Medicine and Health, Division of Neuroscience and Experimental Psychology, School of Biological Sciences, University of Manchester, Manchester Academic Health Science Centre, Manchester, United Kingdom

[i] Department of Medicine, University of Cambridge, United Kingdom

For peer review only

[j] Early Intervention Service, Birmingham Women's and Children's NHS Foundation Trust

[k] NIHR Bristol Biomedical Research Centre, Bristol, United Kingdom

[l] Avon and Wiltshire Mental Health Partnership NHS Trust, Bristol, United Kingdom

† Joint senior authors

* **Corresponding author and address:** Prof Golam M. Khandaker, MRC Integrative Epidemiology Unit, Oakfield House, Oakfield Grove, Clifton, Bristol, BS8 2BN, UK

Email: golam.khandaker@bristol.ac.uk

For peer review only

For peer review only - http://bmjopen.bmj.com/site/about/guidelines.xhtml

ABSTRACT

Introduction: Evidence suggests a potentially causal role of interleukin 6 (IL-6), a pleiotropic cytokine that generally promotes inflammation, in the pathogenesis of psychosis. However, no interventional studies in patients with psychosis, stratified using inflammatory markers, have been conducted to assess the therapeutic potential of targeting IL-6 in psychosis and to elucidate potential mechanism of effect. Tocilizumab is a humanised monoclonal antibody targeting the IL-6 receptor to inhibit IL-6 signalling licensed in the UK for treatment of rheumatoid arthritis. The primary objective of this study is to test whether IL-6 contributes to the pathogenesis of first episode psychosis, and to examine potential mechanisms by which IL-6 affects psychotic symptoms. A secondary objective is to examine characteristics of inflammation-associated psychosis.

Methods and analysis: A proof-of-concept study employing a randomised, parallel-group, double-blind, placebo-controlled design testing the effect of IL-6 inhibition on anhedonia in patients with psychosis. Approximately 60 participants with diagnosis of schizophrenia and related psychotic disorders (ICD-10 codes F20, F22, F25, F28, F29) with evidence of low-grade inflammation (IL-6 ≥0.7pg/ml) will receive either one intravenous infusion of tocilizumab (4.0mg/kg; max 800mg) or normal saline. Psychiatric measures and blood samples will be collected at baseline, and 7-, 14-, and 28-days post-infusion. Cognitive and neuroimaging data will be collected at baseline and 14 days post-infusion. In addition, approximately 30 patients with psychosis without evidence of inflammation (IL-6 <0.7pg/ml) and 30 matched healthy controls will be recruited to complete identical baseline assessments to allow for comparison of the characteristic features of inflammation-associated psychosis.

Ethics and dissemination: The study is sponsored by the University of Bristol and has been approved by the Cambridge East Research Ethics Committee (reference: 22/EE/0010; IRAS

For peer review only

For peer review only - http://bmjopen.bmj.com/site/about/guidelines.xhtml

project ID: 301682). Study findings will be published in peer-review journals. Findings will also be disseminated by scientific presentation and other means.

Trial registration number: ISRCTN 23256704

KEYWORDS: Psychotic Disorders; Negative Symptoms; Interleukin 6; Immunotherapy; Tocilizumab; Clinical Trial.

For peer review only

ARTICLE SUMMARY

Strengths and limitations of this study

- Adopting a randomised controlled trial (RCT) design and patient selection based on elevated level of IL-6 (in addition to other criteria) will help examine the causal role of IL-6, and the therapeutic potential of targeting IL-6 pathway, in psychosis.
- The use of target specific intervention (anti-IL6R monoclonal antibody tocilizumab) will help assess the clinical relevance of IL-6 and related up and downstream inflammatory cytokines in psychosis.
- The use of neuroimaging, cognitive tests, and extensive peripheral blood biomarker exploration before and after tocilizumab treatment to assess potential mechanisms of effect.
- One dose of tocilizumab is unlikely to be sufficient to test the efficacy of this drug as potential treatment for psychosis, which is not the intention of this study.
- Tocilizumab inhibits both IL-6 classical and trans signalling, and consequently the trial will not be able to distinguish between the effects of modulating these two signalling pathways specifically in psychosis.

For peer review only - http://bmjopen.bmj.com/site/about/guidelines.xhtml

Word count: 4,336

INTRODUCTION

Scientific background and study rationale

The neuroimmune hypothesis of schizophrenia proposes that mild peripheral immune activation gives rise to an inflammatory response in the brain and neurobiological changes associated with psychotic illness [1–4]. Meta-analytic evidence is clear that circulating concentrations of interleukin 6 (IL-6) and other inflammatory proteins, such as C-reactive protein (CRP), are increased in patients with psychosis, including treatment naïve first episode psychosis (FEP) [5], compared with controls [6–9]. Prospective cohort studies show that these indices of mild immune activation precede the onset of symptoms [10,11]. Furthermore, genetic variants known to increase IL-6 concentrations are associated with genetic risk of schizophrenia [12,13]. These Mendelian randomization studies eliminate the possibility that raised IL-6 concentrations are a consequence of environmental exposures associated with schizophrenia, such as obesity and smoking and instead suggest that IL-6 has a causal role in psychosis. Extending this approach using the UK Biobank population, we found that genetically-predicted levels of IL-6 were associated with reduced grey matter primarily in the middle temporal gyrus, a region whose gene expression profile is enriched for IL-6 pathway proteins and for neuropsychiatric disorder ontologies [14]. Moreover, clinical studies report correlations between IL-6 levels and structural brain changes in individuals with schizophrenia [15], with reduced grey matter volume being exaggerated in patients with psychosis and elevated inflammatory cytokines [16]. Though this causal evidence strongly implicates IL-6, only an intervention study in patients can test the causal hypothesis.

The neuroimmune hypothesis generally assumes that microglia, the brain's resident immune cells, are activated and pathogenic in schizophrenia. This is supported by traditional

For peer review only

neuropathological studies and initial in-vivo PET imaging studies [17–19], possibly reflecting impaired cellular control of inflammation or oxidative defence. Inflammatory damage may also account for evidence of oxidative stress from MRS glutathione studies [20]. However, whether microglia are the direct target of IL-6 is unclear and it is not certain that IL-6 can cross the blood-brain barrier and/or increase its permeability to circulating inflammatory cells, cytokines, and chemokines [2,21]. Additionally, it is increasingly uncertain whether microglial inflammation, as traditionally understood, occurs in schizophrenia [22,23]. Recent meta-analyses of PET radioligand binding studies report decreased rather than increased radioligand binding to activated microglia [24,25]. This may account for the unexpected lack of therapeutic benefit of the anti-microglial antibiotic, minocycline, in recent large clinical trials [26,27]. Furthermore, large transcriptomic studies in post-mortem brains report no change or reduction in microglial gene expression but increases in astrocytic expression [23,28–32]. It is increasingly understood that both peripheral immune responses and brain glial function are regulated by specialised T cells (Tregs), a subset of which reside in brain parenchyma [33,34]. A novel proposal is that Treg hypofunction accounts for mild peripheral immune disinhibition and dysregulated astroglial-microglial interaction, such that microglia are driven into a developmental, synapse-pruning phenotype while astroglia disrupt neurotransmitter function [33,34]. Importantly, there are bidirectional interactions between IL-6 and Treg function [34]. Crucially, we will measure IL-6 in addition to cellular and molecular markers of immune function and investigate how they correlate with central markers and clinical state.

Previous attempts testing the inflammatory hypothesis in therapeutic clinical trials have been attempted. However, little evidence of overall efficacy has been found [35]. These trials have generally tested broad spectrum agents, such as non-steroidal anti-inflammatory drugs, with no attempt to stratify patients according to evidence of inflammation. A trial

For peer review only

using tocilizumab, a humanised monoclonal antibody (mAb) against the IL-6 receptor currently licensed in the UK for treatment of rheumatoid arthritis (RA) and severe coronavirus disease, reported no improvements in any clinical measure in a small sample of 36 patients with established schizophrenia [36]. However, as mentioned previously, no stratification by inflammatory markers or any mechanistic immune measures was applied. Low-grade inflammation is associated with poor response to antipsychotic drugs [37], but immunotherapy is unlikely to be relevant for all patients with psychosis. Meta-analysis suggests that evidence of immune activation, defined by elevated CRP levels, is present in a quarter to one third of patients with schizophrenia [38]. A randomised controlled trial of infliximab, an anti-tumour necrosis factor alpha (TNF-α) mAb, reported that antidepressant response was associated with higher CRP levels at baseline [39], suggesting that patients with evidence of immune activation may be better candidates for immunotherapy trials. As far as we are aware, no previous clinical trial has selected patients with schizophrenia based on evidence of immune activation.

Selection of patients with particular symptom profiles and/or stage of illness may also be a useful strategy that needs to be employed in immunotherapy trials for schizophrenia. A wide variety of symptoms occur in schizophrenia such as hallucinations, delusions, anhedonia, cognitive dysfunction, and affective symptoms and presentation of these symptoms differ from one individual to another. Some symptoms may be more related to inflammation than others. For instance, meta-analytic data suggests that elevated proinflammatory cytokines are associated with negative psychotic symptomatology [40]. Moreover, a recent study from the ALSPAC birth cohort reported that out of 20 positive and negative symptoms, CRP is particularly associated with anhedonia and auditory hallucinations [41]. Lastly, results from work we have completed to date as part of the MRC-funded larger PIMS collaboration (MR/S037675/1), suggest that anhedonia may be a

For peer review only - http://bmjopen.bmj.com/site/about/guidelines.xhtml

promising target in early phases of established psychotic disorder. Anhedonia and amotivation are strongly associated with poor functional outcomes in depression and schizophrenia, and present a formidable barrier to returning to work or building relationships [42,43]. Patients with psychotic disorders also present with cognitive deficits in a range of domains [44]. Available antipsychotic medications have a limited effects on poor cognitive functioning in psychosis [45]. Illness stage may also be of relevance. Meta-analytic data has revealed no differences in IL-6 levels between stable, medicated patients with schizophrenia and controls, although compared with controls, IL-6 levels were similarly elevated in patients with FEP and those with acute relapse [7]. A separate meta-analysis found evidence of elevated blood cytokine levels in acutely and chronically ill patients with schizophrenia [6]. Focusing on particular inflammation-related symptoms, such as anhedonia, and/or illness stage may increase the chance of success for immunotherapy trials.

Proposed study

The proposed study is a UK multi-site (Birmingham, Bristol, and Cambridge) proof-of-concept, randomised, parallel-group, double-blind, placebo-controlled trial.

Study aims and hypotheses

The primary aim of this trial is to examine potential mechanisms by which IL-6 affects anhedonia, psychotic symptoms, and cognition. Our primary hypothesis is that inhibition of IL-6 signalling with a single intravenous infusion of anti-IL6R monoclonal antibody, tocilizumab, in individuals with psychosis and elevated IL-6 at baseline will attenuate symptoms of anhedonia and amotivation in patients with psychosis, relative to placebo. This will provide further evidence for a potential causal role of inflammation in psychosis. Our secondary hypothesis is that reduction in peripheral inflammation after tocilizumab infusion

For peer review only

in patients with psychosis and evidence of inflammation will be associated with central measures of oxidative stress and relevant resting state brain function.

We will also conduct deep immunophenotyping of peripheral blood mononuclear cell subsets ($CD4^+$, $CD8^+$, Tregs, natural killer and natural killer-T cells, monocytes, and B cells) to characterise their absolute number, frequency, and function. The level of IL-6/STAT3 signalling inhibition within both innate and adaptive immune cells will also be examined using multi-colour flow cytometry with an established optimised pSTAT3 phosflow assay. This will help identify the potential cellular impact of peripheral inflammation in psychosis, which is largely unknown. Functional assessment of IL-6/STAT3 signalling in immune cell subsets and their response to exogenous IL-6 stimulation will inform abnormal immune response in psychosis and allow measurement of response to tocilizumab at the cellular level.

A secondary objective is to carry out an observational study to examine clinical and biomarker differences and similarities between patients with psychotic disorder with and without evidence of inflammation and healthy controls (HCs). We hypothesise that individuals with psychotic disorder and evidence of inflammation, compared to those without evidence of inflammation and HCs, will have increased symptoms of anhedonia and amotivation, poorer cognitive functioning, and cellular and brain-based measures of immune dysfunction.

For peer review only

METHODS

This protocol has been prepared in accordance with the Standard Protocol Items: Recommendations for Interventional Trials (SPIRIT) 2013 statement [46]. Please see supplementary eTable 1 for the SPIRIT checklist. The planned start date for the PIMS trial was 1st November 2021, however, this was delayed due to the COVID-19 pandemic. We began recruiting at our site in Birmingham in November 2022, and we soon expect

For peer review only - http://bmjopen.bmj.com/site/about/guidelines.xhtml

recruitment to begin at our Bristol and Cambridge sites. The planned end date is 31st May 2024.

Patient and public involvement

The study protocol was prepared in collaboration with individuals with lived experience of mental illness who contributed to the development of participant information sheet, consent forms (Appendix I, II, and III), and data collection procedures.

Study design and sample

See Figure 1 for an overview of study design. Individuals residing in Birmingham, Bristol, or Cambridge in the United Kingdom will be recruited. Approximately 60 participants with first episode psychosis and evidence of inflammation (i.e., IL-6 ≥0.7pg/ml) will be randomised to receive either one intravenous infusion of tocilizumab (drug) or normal saline (placebo). For the secondary, observational study, we will compare baseline characteristics of the intervention cohort with approximately 30 participants with psychosis without evidence of inflammation (i.e., IL-6 <0.7pg/ml), and approximately 30 HCs across Birmingham and Cambridge. Participants without evidence of inflammation and controls will not be randomised as they will not receive any intervention. Neuroimaging will only be undertaken by those without MRI contraindications who have given specific informed consent for MRI. Participants not eligible or not consenting for MRI will take part in all other aspects of the study.

Intervention

Single intravenous infusion of tocilizumab (4.0mg/kg; max 800mg in total) or normal saline given to participants with psychosis and evidence of inflammation. Tocilizumab blocks both

For peer review only

IL-6 classic and trans-signalling – the latter being responsible for most of the inflammatory effects of IL-6 – providing broad inhibition of IL-6 signalling and a strong test of a casual role for IL-6 in psychosis [47]. Tocilizumab is the first-in-class, humanized monoclonal antibody against the IL-6R, commercially available and licensed in the UK for treatment of RA. Approved dosage of tocilizumab for treatment of RA is 2, 4, or 8mg/kg; max 800mg in total. In RA, a single tocilizumab infusion has shown to improve clinical and laboratory measures within 48 hours, with most noticeable results in one-to-two weeks [48,49]. The follow-up schedule for our study is in keeping with this observation.

For peer review only

Eligibility criteria

We will recruit participants aged 18-40 years. Patient participants must meet International Classification of Diseases 10th Revision (ICD-10) criteria for a diagnosis of schizophrenia and related psychoses (ICD-10 code F20, F22, F25, F28, F29) at the time of eligibility assessment, be within three years of first diagnosis of psychotic disorder, be on a stable treatment regime with no recent (within two weeks) initiation, cessation, or change in class of antipsychotic medication, and have a Positive and Negative Syndrome Scale (PANSS) item score ≥3 on P1 (delusions), P2 (conceptual disorganisation), P3 (hallucinatory behaviour), or P6 (suspiciousness/persecution). Additionally, patients recruited to the interventional arm will be required to have serum IL-6 levels ≥0.7pg/ml and a Temporal Experience of Pleasure Scale (TEPS) anticipatory pleasure score ≤41 (based on item numbers 1, 3, 7, 11, 12, 14, 15, 16, 17, and 8) and consummatory pleasure score ≤36 (based on item numbers 2, 4, 5, 6, 8, 9, 10, and 13). The threshold of serum IL-6 ≥0.7pg/mL as evidence of inflammation for this particular trial was chosen based on observations from the Personalised Prognostic Tools for Early Psychosis Management (PRONIA) cohort [https://www.pronia.eu]. In 192 first-episode psychosis patients included in the PRONIA study, the median value of serum IL-6 was

For peer review only - http://bmjopen.bmj.com/site/about/guidelines.xhtml

0.49pg/mL (25th percentile 0.22pg/mL; 75th percentile 1.11pg/mL), and the mean was 0.79pg/mL (SD ± 0.84). Based on these observations, we chose the cut-off of 0.7pg/mL for patient selection in current trial. Finally, COVID-19 anti-body titre test will be used to determine adequate levels of immune response via the following cut-offs (for poor response): 400IU Roche/700IU Abbot assay.

HCs will have no current or lifetime history of psychiatric diagnosis, as determined by the Mini-International Neuropsychiatric Interview (MINI). See Table 1 for complete inclusion and exclusion criteria. HCs will be matched to patient participants at the group level by age and sex.

Table 1. PIMS Trial inclusion and exclusion criteria.

Group	Inclusion criteria	Exclusion criteria
All participants	- Provide informed consent.<br>- Understand written and spoken English.<br>- Able and willing to consent to blood sampling.<br>- Willing to abstain from strenuous exercise for 72 hours prior to assessment.	- Pregnancy (confirmed by urine pregnancy test) or breast feeding.<br>- Body mass index >35.<br>- Current or lifetime diagnosis of antisocial personality disorder, autism or other neurodevelopmental disorder, major traumatic brain injury.<br>- Currently active diagnosed eating disorder likely to compromise ability to take part.<br>- History of alcohol or substance use disorder (abuse/dependence) within six months prior to eligibility assessment (nicotine and caffeine dependence are not exclusionary).<br>- Current use of medication likely to compromise interpretation of immunological data.<br>- Known active current or history of recurrent bacterial, viral, fungal, mycobacterial, or other opportunistic infections.<br>- Current infection with VZV, TB, Hepatitis B, Hepatitis C, or HIV confirmed by blood test. Chest X-ray will also be performed to assess for TB.<br>- Any major episode of infection requiring hospitalisation or treatment with IV antibiotics within 4 weeks of eligibility assessment.

For peer review only

		- Unstable cardiac, pulmonary, renal, hepatic, endocrine, hematologic, or active infectious disease, including current or prior malignancy.<br>- Diverticulitis, inflammatory bowel disease, or uncontrolled gastric/duodenal ulcer.<br>- Concomitant auto-immune or auto-inflammatory rheumatological disease.<br>- Concomitant treatment with any biologic drugs.<br>- Current and active ischemic heart disease.<br>- Uncontrolled hypertension defined as systolic blood pressure > 170 or diastolic blood pressure > 110.<br>- History of severe allergic or anaphylactic reactions to human, humanized or murine monoclonal antibodies.<br>- No history of chicken pox infection or no history of varicella zoster immunity.
Additional criteria for neuroimaging (optional)	- Able and willing to consent to MRI scanning	- Contraindications to MRI.
Additional criterion for healthy controls	- No current or lifetime psychiatric diagnosis.	
Additional criteria for all individuals with psychosis	- Meet ICD-10 criteria for a diagnosis of schizophrenia and related psychoses (code F20, F22, F25, F28, F29) at the time of eligibility assessment, as determined by the Mini-International Neuropsychiatric Interview.<br>- Be within three years of first diagnosis of psychotic disorder.<br>- On stable treatment regime with no recent (within 2 weeks) initiation, cessation, or change in class of antipsychotic medication.<br>- No indication or other reason for preclusion into research (e.g., significant risk of suicidal behaviour or risk to others) as determined by their clinical team.<br>- Positive and Negative Syndrome Scale item score ≥3 on P1 (delusions), P2 (conceptual disorganisation), P3 (hallucinatory behaviour), OR P6 (suspiciousness/persecution).	

For peer review only - http://bmjopen.bmj.com/site/about/guidelines.xhtml

Additional criteria for intervention group	- Serum IL-6 level ≥0.7pg/ml at eligibility and baseline assessment.<br>- Temporal Experience of Pleasure Scale anticipatory pleasure score ≤41 (based upon item numbers 1, 3, 7, 11, 12, 14, 15, 16, 17, and 8) and consummatory pleasure score ≤36 (based upon item numbers 2, 4, 5, 6, 8, 9, 10, and 13).<br>- Evidence of COVID-19 immunity required prior to infusion, confirmed before randomisation using evidence of vaccination and antibody titre test.	
Additional criterion for patients with psychosis without inflammation	- Serum IL-6 level <0.7pg/ml at eligibility and baseline assessment.	

For peer review only

Study outcomes

The primary outcome is anhedonia, defined as anticipatory and consummatory pleasure scores, assessed by the TEPS [50] at approximately day 14 post-infusion. We will also collect data on several secondary/exploratory measures including 1) clinical outcomes, namely positive and negative symptoms of psychosis, depressive symptoms, fatigue, general quality of life and subjective wellbeing, 2) cognitive function (psychomotor speed, attention, memory, and executive function), 3) neuroimaging outcomes based on comparisons of brain structure, function, and oxidative stress levels using MRI and MRS outcomes, 4) blood biomarker outcomes, namely peripheral blood inflammatory markers, biochemical assays, including cortisol and cardiometabolic markers, and peripheral blood cellular immunophenotyping, and 5) genetic outcomes including DNA and RNA sequencing and epigenetic mechanism assessment with methylation assays(see Table 2 for more details).Where possible, blood samples will be collected during working hours and time of

For peer review only - http://bmjopen.bmj.com/site/about/guidelines.xhtml

sampling will be recorded. However, a specified time window will not be given to ease burden on patients and to maximise participation.

Table 2. PIMS trial study measures

Domain	Tool	Source	Validated Tool	Time of assessment
Sociodemographic/lifestyle	Screening questionnaire	Self-report		Screening
	Medical History Questionnaire	Self-report/General practice		Eligibility
	Substance Use Questionnaire	Self-report		Eligibility
	Physical Measurements Form	Self-report		Baseline
	Sociodemographic Questionnaire	Self-report		Baseline
Psychiatric	The Temporal Experience of Pleasure Scale	Self-report	✓	Eligibility, baseline, follow-ups
	The Positive and Negative Syndrome Scale	Interviewer assessed	✓	Eligibility, baseline, follow-ups
	The Mini-International Neuropsychiatric Interview	Interviewer assessed	✓	Eligibility
	Psychiatric History Questionnaire	Self-report		Baseline
	The Scale for the Assessment of Negative Symptoms	Self-report	✓	Baseline, follow-ups
	The Calgary Depression Scale for Schizophrenia	Interviewer assessed	✓	Baseline, follow-ups
	Multi-dimensional Fatigue Inventory	Self-report	✓	Baseline, follow-ups
	European Quality of Life-5 Dimensions Three-Level Version	Self-report	✓	Baseline, follow-ups
	Visual Analogue Scale for Subjective Wellbeing	Self-report	✓	Baseline, follow-ups
Cognitive	National Adult Reading Test for	Interviewer assessed	✓	Baseline, follow-up 2

For peer review only - http://bmjopen.bmj.com/site/about/guidelines.xhtml

For peer review only

	estimated premorbid IQ			
	CANTAB Reaction Time test	Computer task	✓	Baseline, follow-up 2
	Symbol Coding Test	Paper task	✓	Baseline, follow-up 2
	CANTAB Rapid Visual Information Processing test	Computer task	✓	Baseline, follow-up 2
	CANTAB Paired Associates Learning test	Computer task	✓	Baseline, follow-up 2
	CANTAB One Touch Stockings of Cambridge test	Computer task	✓	Baseline, follow-up 2
Biologic	Inflammatory markers, cardiometabolic markers, IDO activation, white cell phenotyping	Laboratory tests		Baseline, follow-ups
Genetic	RNA and DNA sequencing, methylation assay	Blood (RNA, DNA)		Baseline, follow-ups
Neuroimaging	MRI Screening Questionnaire			Baseline, follow-up 2
	Structural MRI, 1H-MRS measure of glutathione in the prefrontal cortex area, resting state fMRI			Baseline, follow-up 2

For peer review only

Sample size and statistical power

We will recruit approximately 60 patients with psychosis. However, currently there are no trials of immunotherapies for anhedonia in schizophrenia making accurate power calculation difficult. This study is a proof-of-concept experiment designed to test whether inhibition of IL-6 signalling leads to changes in psychotic symptoms. It could also inform likely statistical power for future trials testing efficacy of the drug as a treatment of schizophrenia, which is not the intention of this study. The exact statistical tests and techniques that will be applied to

For peer review only - http://bmjopen.bmj.com/site/about/guidelines.xhtml

the data will depend on the objective of specific analysis and data characteristics (e.g., variable type, distribution). These details will be specified in analysis plans and registered online before participants are unblinded and any data analysis is performed.

Randomisation and blinding

An external agency independent of the study team will arrange random allocation to tocilizumab or normal saline group 1:1, ensuring two groups are comparable regarding anhedonia severity and sex. Randomisation will be stratified by site. Randomising agency will provide the randomisation code to the relevant hospital pharmacy who will dispense tocilizumab or normal saline according to the randomisation schedule. Dispensing pharmacies will keep a log of products dispensed. Infusions will be prepared and administered at clinical research facilities (CRFs). Infusion packs will be prepared by trained staff not part of the core study team, ensuring blinding of treatment allocation. Infusion packs containing drug or placebo will be visually indistinguishable from each other, ensuring that both participants and study team remain blind regarding treatment allocation.

For peer review only

Statistical analysis

For randomised participants, an intention-to-treat approach will be taken for data analysis by including all randomised participants in statistical analyses, regardless of the treatment they received (if any). We will compare outcome measures between treatment and placebo groups controlling for baseline scores. This mechanistic experiment will focus on overall pattern of results and their effect sizes rather than *P*-values for individual tests of statistical significance. The secondary mechanistic and observational analysis will compare psychotic symptoms, cognitive function, blood, neuroimaging, and other biomarkers between and across study groups using appropriate statistical tests.

STUDY PROCEDURE

An overview of study procedures is presented in Figure 1 and all study measures are detailed in Table 2. Recruitment will take place in Birmingham, Bristol, and Cambridge and assessments at University and NHS research facilities.

Participant identification

Potential participants with psychosis will be identified by NHS Psychosis Early Intervention (EI) teams. HCs will be recruited through advertisement methods in Birmingham and Cambridge. Potential participants will complete a screening questionnaire to confirm their eligibility to participate. If deemed eligible, participants will be invited to an appointment to complete a full eligibility assessment.

Eligibility assessment

Assessments will be carried out to establish eligibility and to obtain informed consent. Patients will complete the MINI to confirm ICD-10 diagnosis of schizophrenia and related psychoses, the PANSS to confirm the presence of positive symptoms of psychosis, and the TEPS to confirm eligibility based on anticipatory and consummatory pleasure sum scores. A blood sample will be collected from patients for serum IL-6 measurement. An MRI screening questionnaire will be administered to those willing to give informed consent for neuroimaging.

Baseline assessment

All participants (60 inflamed psychosis, 30 non-inflamed psychosis, and 30 HCs) will attend a baseline assessment comprising psychiatric measures, cognitive tasks, blood sampling, and

For peer review only

For peer review only - http://bmjopen.bmj.com/site/about/guidelines.xhtml

neuroimaging (optional). This will be the final study contact for patients without evidence of inflammation and HCs. Patients with evidence of inflammation will undergo further tests to establish safety/eligibility to receive tocilizumab, including a chest X-ray and blood tests to exclude pregnancy and certain infections, such as TB, HIV, and COVID-19. Eligible participants will be randomised and invited for infusion.

Intervention

Intravenous infusion of tocilizumab or normal saline will be given continuously over one hour at CRFs in Birmingham, Bristol, and Cambridge by trained clinical staff under the supervision of a designated study doctor. Participants will remain under clinical observation for a further 1-hour period after the end of infusion.

Follow-up assessments

Follow-up assessments will take place approximately 7-, 14-, and 28-days post-infusion, and will collect similar data to the baseline assessment. Cognitive tasks and neuroimaging (optional) will be administered only on day 14. Around 42 days post-infusion, participants will be contacted by phone to provide a final debrief; at which point they will exit the study.

RISK MANAGEMENT

Psychosis-related risks

All patients will be under the care of a specialist NHS psychosis EI service. Participation will not involve any treatment modifications or significant delays in receiving treatment. If a patient becomes distressed during an assessment, or does not wish to continue for any reason, the researcher will stop the assessment. Participants may withdraw at any time without giving

For peer review only

a reason. If there is any concern for the participant's safety, the research team will liaise with participant's GP and/or mental health team as needed.

Procedure-related risks

Venepuncture

Blood taking is associated with mild discomfort and other side effects are rare. Efforts will be made to minimise discomfort. Blood taking will be performed by a nurse, doctor, or research team member trained in venepuncture.

Chest X-ray

This study will use a typical effective radiation dose of 0.014 mSv; equivalent to 2.5 days of average natural background radiation in the UK. The risk of developing cancer as a consequence of participating in this study is 0.0001%. Only non-pregnant, adult participants will be included.

Neuroimaging

Discomfort during MRI will be minimised by using mirrors to allow participants to view outside of the machine, providing ear plugs and a panic button, and allowing participants to communicate with the researcher and scan operator throughout. Mild transient vertigo may be experienced when being moved into the MRI machine. Risk of dislodgement or malfunction of medical implants or metallic foreign objects will be minimised by screening participants to ensure no metal is present on or within the body.

IL-6 levels

For peer review only - http://bmjopen.bmj.com/site/about/guidelines.xhtml

We expect some 30-50% of patient participants to have evidence of inflammation in the blood (IL-6 ≥0.7pg/ml). This is not a cause for concern. Reasons for elevated IL-6 in the absence of an acute infection or chronic inflammatory illness could include obesity, smoking, alcohol use, and lack of exercise, so knowledge of 'inflammation status' may prompt participants to adopt a healthier lifestyle. If serum IL-6 level is high (i.e., IL-6 ≥0.7pg/ml) along with elevated CRP (>20mg/L) without any apparent explanation, such as infection or chronic inflammatory illness, we will inform the participant's GP and the participant will be excluded from the study.

Risk to research staff

Staff will follow local safety procedures when lone working. No other risks are anticipated.

Safety considerations for infusion and monitoring of adverse reaction

Before infusion

Participants will be selected based on strict inclusion and exclusion criteria. Additionally, we will carry out tests for TB, HIV, VZV antibody, and Hepatitis B and C because, though unlikely after a single dose, tocilizumab could make these infections worse. Female participants of childbearing age will be given a pregnancy test, which must be negative. Participants who are sexually active will be asked to use at least one form of effective contraception for six weeks post-infusion. Male participants will also be asked not to donate sperm samples for six weeks post-infusion.

During infusion

For peer review only

Infusions will be given under supervision of a designated study doctor. Participants will be monitored for possible side effects, which will be managed in line with use of tocilizumab for treating patients with RA.

After infusion

Participants will remain under observation for one-hour post-infusion. Participants will be advised to seek help if they feel unwell after leaving the assessment centre and will be given an information sheet containing a telephone number their health professionals can call. If necessary, we will unblind the participant and inform their health professional whether they received tocilizumab or normal saline. Adverse reactions will be recorded at each follow-up visit. Additional, safety blood tests will be done at second follow-up (e.g., WBC count, liver function, lipids).

ETHICS AND DISSEMINATION

The study will be conducted in accordance with the REC, Health Research Authority (HRA), and local Research and Development (R&D) department approvals and guidelines (REC reference: 22/EE/0010). The study team will prepare protocol amendments as required and ethics approval will be sought before implementing any changes to the approved protocol. The ISRCTN Trial Registry and the Research Governance Office will be informed of any amendments to the protocol.

Consent

Informed consent will be obtained prior to eligibility assessments for participation in the study (Appendix I, II, and II). This will include consent to randomise, for contact with their GP to inform them about participation, access GP/psychiatric records to verify medical

For peer review only - http://bmjopen.bmj.com/site/about/guidelines.xhtml

history to establish eligibility, and to inform the participant's GP any results/outcomes as necessary. Consent for additional tests to establish safety for tocilizumab infusion and for storing biological samples will also be obtained.

Study management

The study is sponsored by the University of Bristol. The sponsor, the Chief Investigator (GMK), and the co-Lead (RU) will have overall responsibility for the study. A named principal investigator will take clinical responsibility for study activities at each site. The study does not require the formal arrangement of a steering committee because, according to the HRA, it is not a Clinical Trial of an Investigational Medicinal Product. However, to enhance monitoring of the study, a study management group will be established, comprising academic and clinical experts in psychiatry, rheumatology, neuroscience, and immunology.

Data management and retention of samples

All potential participants will be assigned a unique study-specific participant ID number. All data will be subject to good practice as laid down in the Data Protection Act. Each study stage is tracked so that participant's (de-identified) status within the study is known, and assessment and other appointment dates are forecasted. This information is held on a secure, password-protected database. Anonymised data from assessments will be uploaded to a secure, password-protected database using secure web-based data entry systems. Minimal personal data (age, sex) will be indexed by each participant's unique ID number. Blood samples collected in this study may be stored for up to 10 years after the completion for additional research. Stored samples will be coded throughout the sample storage and analysis process and will not be labelled with personal identifiers. Participants may withdraw their consent for their samples to be stored for future research.

For peer review only

Dissemination plan

Study results will be published in peer-review journals and will conform to the guidelines of the International Committee of Medical Journal Editors. Findings will be disseminated at conferences, departmental talks, and via social and traditional media.

AUTHORS CONTRIBUTIONS

ÉMF wrote first draft of the PIMS trial protocol and of this manuscript. SLG, MK, GKM, BD, DJ, JS, and NMB contributed to study design and protocol development and revised manuscript drafts. RU contributed to study design and study protocol, and revised manuscript drafts. GMK devised study design and trial protocol, and revised drafts. ÉMF and SLG developed study materials and liaised with REC and HRA regarding approvals. AM, JR, FCZ, HH, EW, and MW contributed to the revision of the manuscript and validation of operating procedures and mechanistic protocols. RU and GMK co-lead the MRC grant that funds the PIMS trial and provide overall supervision and oversight for the project.

FUNDING STATEMENT

The PIMS trial is funded by a Medical Research Council (MRC) grant to RU and GMK; Grant Ref: MR/S037675/1. ÉMF is supported by an MRC Integrative Epidemiology Unit PhD Studentship. AM is supported by funding from the MRC for doctoral training (MR/2434208). FCZ receives a PhD Fellowship from the São Paulo Research Foundation (2019/13229-2 and 2021/07448-3). HH is supported by the PIMS Trial MRC grant (MR/S037675/1). DJ is supported by the Cambridge Arthritis Research Endeavour and the National Institute for Health Research (NIHR) Cambridge Biomedical Research Centre (BRC-1215-20014). NMB acknowledges funding support from the MRC (MR/R006008/1

For peer review only

and MR/N019016/1), Ministry of Defence (702931454), Diabetes UK (20/0006296), NIHR (14/WM/0093), and Innovate UK (84361). RU has grants from MRC, NIHR: Health Technology Assessment, European Commission - Research: The Seventh Framework Programme, and personal fees from Sunovion, outside the submitted work. GMK acknowledges funding support from the Wellcome Trust (Grant No. 201486/Z/16/Z), the Medical Research Council, UK (Grant No. MC_UU_00011/1; Grant No. MR/S037675/1; and Grant No. MR/W014416/1), and the National Institute of Health Research Bristol Biomedical Research Centre, UK (Grant No. NIHR203315). The funders had no role in the design of this study.

COMPETING INTERESTS STATEMENT

ÉMF, SLG, AM, JR, FCZ, HH, EW, MW, MK, GKM, BD, DJ, JS, RU, and GMK have no conflicts of interest to report. NMB holds shares and is a Director of Celentyx Ltd.

PIMS COLLABORATION

Members of the PIMS Collaboration include Golam Khandaker, Rachel Upthegrove, Alice Egerton, Anthony David, Bill Deakin, Carmine Pariante, David Cotter, Ed Bullmore, Eva Meisenzahl, Gary Donohoe, Georgios Gkoutos, Jack Rogers, James MacCabe, Joanna Neill, John Suckling, Neil Harrison, Nicholas Barnes, Nikos Koutsouleris, Paola Dazzan, Peter Jones, Stephen Burgess, Stephen Wood, Valeria Mondelli.

REFERENCES

1 Khandaker GM, Cousins L, Deakin J, *et al.* Inflammation and immunity in schizophrenia: implications for pathophysiology and treatment. *Lancet Psychiatry* 2015;**2**:258–70. doi:10.1016/S2215-0366(14)00122-9

For peer review only - http://bmjopen.bmj.com/site/about/guidelines.xhtml

2 Khandaker GM, Dantzer R. Is there a role for immune-to-brain communication in schizophrenia? *Psychopharmacology* 2016;**233**:1559–73. doi:10.1007/s00213-015-3975-1

3 Khandaker GM, Dantzer R, Jones PB. Immunopsychiatry: important facts. *Psychol Med* 2017;**47**:2229–37. doi:10.1017/S0033291717000745

4 Upthegrove R, Khandaker G. Cytokines, Oxidative Stress and Cellular Markers of Inflammation in Schizophrenia. *Current topics in behavioral neurosciences* 2020;**44**:49–66. doi:10.1007/7854_2018_88

5 Upthegrove R, Manzanares-Teson N, Barnes NM. Cytokine function in medication-naive first episode psychosis: a systematic review and meta-analysis. *Schizophr Res* 2014;**155**:101–8. doi:10.1016/j.schres.2014.03.005

6 Goldsmith DR, Rapaport MH, Miller BJ. A meta-analysis of blood cytokine network alterations in psychiatric patients: comparisons between schizophrenia, bipolar disorder and depression. *Mol Psychiatry* 2016;**21**:1696–709. doi:10.1038/mp.2016.3

7 Miller BJ, Buckley P, Seabolt W, *et al.* Meta-analysis of cytokine alterations in schizophrenia: clinical status and antipsychotic effects. *Biological psychiatry* 2011;**70**:663–71. doi:10.1016/j.biopsych.2011.04.013

8 Potvin S, Stip E, Sepehry AA, *et al.* Inflammatory cytokine alterations in schizophrenia: a systematic quantitative review. *Biol Psychiatry* 2008;**63**:801–8. doi:10.1016/j.biopsych.2007.09.024

9 Wang AK, Miller BJ. Meta-analysis of Cerebrospinal Fluid Cytokine and Tryptophan Catabolite Alterations in Psychiatric Patients: Comparisons Between Schizophrenia, Bipolar Disorder, and Depression. *Schizophr Bull* 2018;**44**:75–83. doi:10.1093/schbul/sbx035

10 Khandaker GM, Zimbron J, Dalman C, *et al.* Childhood infection and adult schizophrenia: a meta-analysis of population-based studies. *Schizophr Res* 2012;**139**:161–8. doi:10.1016/j.schres.2012.05.023

11 Khandaker GM, Zimbron J, Lewis G, *et al.* Prenatal maternal infection, neurodevelopment and adult schizophrenia: a systematic review of population-based studies. *Psychol Med* 2013;**43**:239–57. doi:10.1017/S0033291712000736

12 Khandaker GM, Zammit S, Burgess S, *et al.* Association between a functional interleukin 6 receptor genetic variant and risk of depression and psychosis in a population-based birth cohort. *Brain Behav Immun* 2018;**69**:264–72. doi:10.1016/j.bbi.2017.11.020

13 Perry BI, Upthegrove R, Kappelmann N, *et al.* Associations of immunological proteins/traits with schizophrenia, major depression and bipolar disorder: A bi-directional two-sample mendelian randomization study. *Brain, Behavior, and Immunity* 2021;**97**:176–85. doi:10.1016/j.bbi.2021.07.009

14 Williams JA, Burgess S, Suckling J, *et al.* Inflammation and Brain Structure in Schizophrenia and Other Neuropsychiatric Disorders: A Mendelian Randomization Study. *JAMA Psychiatry* 2022;**79**:498. doi:10.1001/jamapsychiatry.2022.0407

For peer review only - http://bmjopen.bmj.com/site/about/guidelines.xhtml

15 Kose M, Pariante CM, Dazzan P, *et al.* The Role of Peripheral Inflammation in Clinical Outcome and Brain Imaging Abnormalities in Psychosis: A Systematic Review. *Front Psychiatry* 2021;**12**:612471. doi:10.3389/fpsyt.2021.612471

16 Lesh TA, Careaga M, Rose DR, *et al.* Cytokine alterations in first-episode schizophrenia and bipolar disorder: relationships to brain structure and symptoms. *Journal of Neuroinflammation* 2018;**15**:165. doi:10.1186/s12974-018-1197-2

17 Trépanier MO, Hopperton KE, Mizrahi R, *et al.* Postmortem evidence of cerebral inflammation in schizophrenia: a systematic review. *Mol Psychiatry* 2016;**21**:1009–26. doi:10.1038/mp.2016.90

18 van Kesteren CFMG, Gremmels H, de Witte LD, *et al.* Immune involvement in the pathogenesis of schizophrenia: a meta-analysis on postmortem brain studies. *Transl Psychiatry* 2017;**7**:e1075–e1075. doi:10.1038/tp.2017.4

19 Marques TR, Ashok AH, Pillinger T, *et al.* Neuroinflammation in schizophrenia: meta-analysis of in vivo microglial imaging studies. *Psychological Medicine* 2019;**49**:2186–96. doi:10.1017/S0033291718003057

20 Murray AJ, Rogers JC, Katshu MZUH, *et al.* Oxidative Stress and the Pathophysiology and Symptom Profile of Schizophrenia Spectrum Disorders. *Frontiers in Psychiatry* 2021;**12**.https://www.frontiersin.org/articles/10.3389/fpsyt.2021.703452 (accessed 9 Aug 2022).

21 Upthegrove R. The immune system and schizophrenia: An update for clinicians. *Advances in Psychiatric Treatment* 2014;**20**:83–91. doi:10.1192/apt.bp.113.011452

22 Conen S, Gregory CJ, Hinz R, *et al.* Neuroinflammation as measured by positron emission tomography in patients with recent onset and established schizophrenia: implications for immune pathogenesis. *Mol Psychiatry* 2021;**26**:5398–406. doi:10.1038/s41380-020-0829-y

23 van Mierlo HC, Schot A, Boks MPM, *et al.* The association between schizophrenia and the immune system: Review of the evidence from unbiased 'omic-studies.' *Schizophrenia Research* 2020;**217**:114–23. doi:10.1016/j.schres.2019.05.028

24 Plavén-Sigray P, Matheson GJ, Collste K, *et al.* Positron Emission Tomography Studies of the Glial Cell Marker Translocator Protein in Patients With Psychosis: A Meta-analysis Using Individual Participant Data. *Biological Psychiatry* 2018;**84**:433–42. doi:10.1016/j.biopsych.2018.02.1171

25 Plavén-Sigray P, Matheson GJ, Coughlin JM, *et al.* Meta-analysis of the Glial Marker TSPO in Psychosis Revisited: Reconciling Inconclusive Findings of Patient-Control Differences. *Biol Psychiatry* 2021;**89**:e5–8. doi:10.1016/j.biopsych.2020.05.028

26 Deakin B, Suckling J, Barnes TRE, *et al.* The benefit of minocycline on negative symptoms of schizophrenia in patients with recent-onset psychosis (BeneMin): a randomised, double-blind, placebo-controlled trial. *The Lancet Psychiatry* 2018;**5**:885–94. doi:10.1016/S2215-0366(18)30345-6

For peer review only

For peer review only - http://bmjopen.bmj.com/site/about/guidelines.xhtml

27 Weiser M, Levi L, Burshtein S, *et al.* The effect of minocycline on symptoms in schizophrenia: Results from a randomized controlled trial. *Schizophrenia Research* 2019;**206**:325–32. doi:10.1016/j.schres.2018.10.023

28 Ramaker RC, Bowling KM, Lasseigne BN, *et al.* Post-mortem molecular profiling of three psychiatric disorders. *Genome Medicine* 2017;**9**:72. doi:10.1186/s13073-017-0458-5

29 González-Peñas J, Costas J, Villamayor MJG, *et al.* Enrichment of rare genetic variants in astrocyte gene enriched co-expression modules altered in postmortem brain samples of schizophrenia. *Neurobiology of Disease* 2019;**121**:305–14. doi:10.1016/j.nbd.2018.10.013

30 Gandal MJ, Zhang P, Hadjimichael E, *et al.* Transcriptome-wide isoform-level dysregulation in ASD, schizophrenia, and bipolar disorder. *Science* 2018;**362**:eaat8127. doi:10.1126/science.aat8127

31 Gandal MJ, Haney JR, Parikshak NN, *et al.* Shared molecular neuropathology across major psychiatric disorders parallels polygenic overlap. *Science* 2018;**359**:693–7. doi:10.1126/science.aad6469

32 Toker L, Mancarci BO, Tripathy S, *et al.* Transcriptomic Evidence for Alterations in Astrocytes and Parvalbumin Interneurons in Subjects With Bipolar Disorder and Schizophrenia. *Biological Psychiatry* 2018;**84**:787–96. doi:10.1016/j.biopsych.2018.07.010

33 Corsi-Zuelli F, Deakin B. Impaired regulatory T cell control of astroglial overdrive and microglial pruning in schizophrenia. *Neuroscience & Biobehavioral Reviews* 2021;**125**:637–53. doi:10.1016/j.neubiorev.2021.03.004

34 Corsi-Zuelli F, Deakin B, de Lima MHF, *et al.* T regulatory cells as a potential therapeutic target in psychosis? Current challenges and future perspectives. *Brain, Behavior, & Immunity - Health* 2021;**17**:100330. doi:10.1016/j.bbih.2021.100330

35 Jeppesen R, Christensen RHB, Pedersen EMJ, *et al.* Efficacy and safety of anti-inflammatory agents in treatment of psychotic disorders – A comprehensive systematic review and meta-analysis. *Brain, Behavior, and Immunity* 2020;**90**:364–80. doi:10.1016/j.bbi.2020.08.028

36 Girgis RR, Ciarleglio A, Choo T, *et al.* A Randomized, Double-Blind, Placebo-Controlled Clinical Trial of Tocilizumab, An Interleukin-6 Receptor Antibody, For Residual Symptoms in Schizophrenia. *Neuropsychopharmacology : official publication of the American College of Neuropsychopharmacology* 2018;**43**:1317–23. doi:10.1038/npp.2017.258

37 Mondelli V, Ciufolini S, Belvederi Murri M, *et al.* Cortisol and Inflammatory Biomarkers Predict Poor Treatment Response in First Episode Psychosis. *Schizophrenia bulletin* 2015;**41**:1162–70. doi:10.1093/schbul/sbv028

38 Miller BJ, Culpepper N, Rapaport MH. C-reactive protein levels in schizophrenia: a review and meta-analysis. *Clinical schizophrenia & related psychoses* 2014;**7**:223–30. doi:10.3371/CSRP.MICU.020813

For peer review only - http://bmjopen.bmj.com/site/about/guidelines.xhtml

39 Raison CL, Rutherford RE, Woolwine BJ, *et al.* A randomized controlled trial of the tumor necrosis factor antagonist infliximab for treatment-resistant depression: The role of baseline inflammatory biomarkers. *JAMA Psychiatry* 2013;**70**:31–41. doi:10.1001/2013.jamapsychiatry.4

40 Dunleavy C, Elsworthy RJ, Upthegrove R, *et al.* Inflammation in first-episode psychosis: The contribution of inflammatory biomarkers to the emergence of negative symptoms, a systematic review and meta-analysis. *Acta Psychiatrica Scandinavica* 2022;**146**:6–20. doi:10.1111/acps.13416

41 Khandaker GM, Stochl J, Zammit S, *et al.* Association between circulating levels of C-reactive protein and positive and negative symptoms of psychosis in adolescents in a general population birth cohort. *J Psychiatr Res* 2021;**143**:534–42. doi:10.1016/j.jpsychires.2020.11.028

42 Edwards CJ, Cella M, Tarrier N, *et al.* Investigating the empirical support for therapeutic targets proposed by the temporal experience of pleasure model in schizophrenia: A systematic review. *Schizophrenia research* 2015;**168**:120–44. doi:10.1016/j.schres.2015.08.013

43 Lalousis PA, Schmaal L, Wood SJ, *et al.* Neurobiologically Based Stratification of Recent-Onset Depression and Psychosis: Identification of Two Distinct Transdiagnostic Phenotypes. *Biological Psychiatry* 2022;:S0006322322011568. doi:10.1016/j.biopsych.2022.03.021

44 Fioravanti M, Carlone O, Vitale B, *et al.* A Meta-Analysis of Cognitive Deficits in Adults with a Diagnosis of Schizophrenia. *Neuropsychol Rev* 2005;**15**:73–95. doi:10.1007/s11065-005-6254-9

45 Keefe RSE, Bilder RM, Davis SM, *et al.* Neurocognitive Effects of Antipsychotic Medications in Patients With Chronic Schizophrenia in the CATIE Trial. *Archives of General Psychiatry* 2007;**64**:633–47. doi:10.1001/archpsyc.64.6.633

46 Chan A-W, Tetzlaff JM, Altman DG, *et al.* SPIRIT 2013 Statement: defining standard protocol items for clinical trials. *Rev Panam Salud Publica* 2015;**38**:506–14.

47 Calabrese LH, Rose-John S. IL-6 biology: implications for clinical targeting in rheumatic disease. *Nature reviews Rheumatology* 2014;**10**:720–7. doi:10.1038/nrrheum.2014.127

48 Choy EHS, Isenberg DA, Garrood T, *et al.* Therapeutic benefit of blocking interleukin-6 activity with an anti-interleukin-6 receptor monoclonal antibody in rheumatoid arthritis: a randomized, double-blind, placebo-controlled, dose-escalation trial. *Arthritis and rheumatism* 2002;**46**:3143–50. doi:10.1002/art.10623

49 Woo P, Wilkinson N, Prieur A-M, *et al.* Open label phase II trial of single, ascending doses of MRA in Caucasian children with severe systemic juvenile idiopathic arthritis: proof of principle of the efficacy of IL-6 receptor blockade in this type of arthritis and demonstration of prolonged clinical improvement. *Arthritis research & therapy* 2005;**7**:R1281-8. doi:10.1186/ar1826

For peer review only

For peer review only - http://bmjopen.bmj.com/site/about/guidelines.xhtml

50 Gard DE, Gard MG, Kring AM, *et al.* Anticipatory and consummatory components of the experience of pleasure: A scale development study. *Journal of Research in Personality* 2006;**40**:1086–102. doi:10.1016/j.jrp.2005.11.001

For peer review only

FIGURE LEGEND

Figure 1. Overview of study design

For peer review only

For peer review only - http://bmjopen.bmj.com/site/about/guidelines.xhtml

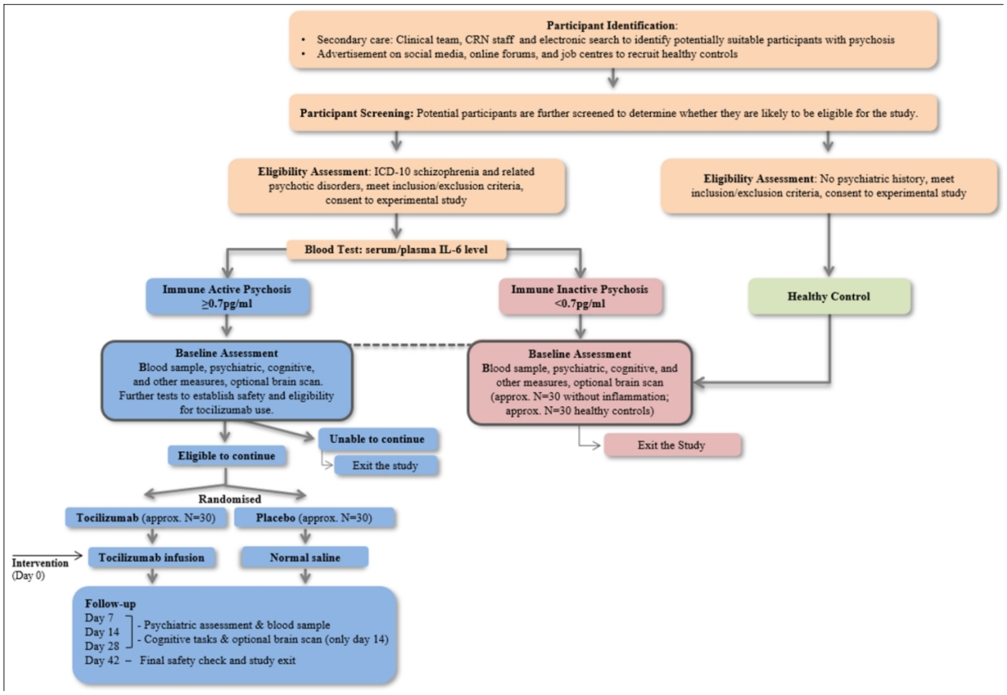

Figure 1. Overview of study design

240x165mm (144 x 144 DPI)

Supplementary Material

Foley et al. Protocol for the Psychosis Immune Mechanism Stratified Medicine (PIMS) trial: A randomised double-blind placebo-controlled trial of single dose tocilizumab in patients with psychosis.

Table of Contents

eTable1: SPIRIT 2013 Checklist 2

Appendix I – Consent Form for Screening: All Participants 8

Appendix II – Informed Consent Form for Study Participation: Patients 10

Appendix III – Informed Consent Form for Study Participation: Healthy Controls 13

For peer review only

eTable1: SPIRIT 2013 Checklist – Recommended items to address in a clinical trial protocol and related documents*

SPIRIT

STANDARD PROTOCOL ITEMS: RECOMMENDATIONS FOR INTERVENTIONAL TRIALS

For peer review only

Section/item	Item No	Description	Addressed on page number
**Administrative information**			
Title	1	Descriptive title identifying the study design, population, interventions, and, if applicable, trial acronym	1
Trial registration	2a	Trial identifier and registry name. If not yet registered, name of intended registry	4
	2b	All items from the World Health Organization Trial Registration Data Set	4 (ISRCTN 23256704)
Protocol version	3	Date and version identifier	-
Funding	4	Sources and types of financial, material, and other support	24 - 25
Roles and responsibilities	5a	Names, affiliations, and roles of protocol contributors	1, 24
	5b	Name and contact information for the trial sponsor	23
	5c	Role of study sponsor and funders, if any, in study design; collection, management, analysis, and interpretation of data; writing of the report; and the decision to submit the report for publication, including whether they will have ultimate authority over any of these activities	23-25

For peer review only - http://bmjopen.bmj.com/site/about/guidelines.xhtml

	5d	Composition, roles, and responsibilities of the coordinating centre, steering committee, endpoint adjudication committee, data management team, and other individuals or groups overseeing the trial, if applicable (see Item 21a for data monitoring committee)	23 - 25
**Introduction**			
Background and rationale	6a	Description of research question and justification for undertaking the trial, including summary of relevant studies (published and unpublished) examining benefits and harms for each intervention	6-9
	6b	Explanation for choice of comparators	6-9
Objectives	7	Specific objectives or hypotheses	9-10
Trial design	8	Description of trial design including type of trial (eg, parallel group, crossover, factorial, single group), allocation ratio, and framework (eg, superiority, equivalence, noninferiority, exploratory)	9
**Methods: Participants, interventions, and outcomes**			
Study setting	9	Description of study settings (e.g., community clinic, academic hospital) and list of countries where data will be collected. Reference to where list of study sites can be obtained	9, 11
Eligibility criteria	10	Inclusion and exclusion criteria for participants. If applicable, eligibility criteria for study centres and individuals who will perform the interventions (eg, surgeons, psychotherapists)	12, Table 1
Interventions	11a	Interventions for each group with sufficient detail to allow replication, including how and when they will be administered	11, 19, 21-22, Figure 1
	11b	Criteria for discontinuing or modifying allocated interventions for a given trial participant (eg, drug dose change in response to harms, participant request, or improving/worsening disease)	22
	11c	Strategies to improve adherence to intervention protocols, and any procedures for monitoring adherence (eg, drug tablet return, laboratory tests)	-
	11d	Relevant concomitant care and interventions that are permitted or prohibited during the trial	12, Table 1

For peer review only - http://bmjopen.bmj.com/site/about/guidelines.xhtml

Outcomes	12	Primary, secondary, and other outcomes, including the specific measurement variable (eg, systolic blood pressure), analysis metric (eg, change from baseline, final value, time to event), method of aggregation (eg, median, proportion), and time point for each outcome. Explanation of the clinical relevance of chosen efficacy and harm outcomes is strongly recommended	9-10, Figure 1, Table 2
Participant timeline	13	Time schedule of enrolment, interventions (including any run-ins and washouts), assessments, and visits for participants. A schematic diagram is highly recommended (see Figure)	18-19, Figure 1
Sample size	14	Estimated number of participants needed to achieve study objectives and how it was determined, including clinical and statistical assumptions supporting any sample size calculations	17
Recruitment	15	Strategies for achieving adequate participant enrolment to reach target sample size	18
**Methods: Assignment of interventions (for controlled trials)**			
Allocation:			
Sequence generation	16a	Method of generating the allocation sequence (eg, computer-generated random numbers), and list of any factors for stratification. To reduce predictability of a random sequence, details of any planned restriction (eg, blocking) should be provided in a separate document that is unavailable to those who enrol participants or assign interventions	17
Allocation concealment mechanism	16b	Mechanism of implementing the allocation sequence (eg, central telephone; sequentially numbered, opaque, sealed envelopes), describing any steps to conceal the sequence until interventions are assigned	17
Implementation	16c	Who will generate the allocation sequence, who will enrol participants, and who will assign participants to interventions	17
Blinding (masking)	17a	Who will be blinded after assignment to interventions (eg, trial participants, care providers, outcome assessors, data analysts), and how	17
	17b	If blinded, circumstances under which unblinding is permissible, and procedure for revealing a participant's allocated intervention during the trial	22

**Methods: Data collection, management, and analysis**			
Data collection methods	18a	Plans for assessment and collection of outcome, baseline, and other trial data, including any related processes to promote data quality (eg, duplicate measurements, training of assessors) and a description of study instruments (eg, questionnaires, laboratory tests) along with their reliability and validity, if known. Reference to where data collection forms can be found, if not in the protocol	10-15, 18-19, Table 2
	18b	Plans to promote participant retention and complete follow-up, including list of any outcome data to be collected for participants who discontinue or deviate from intervention protocols	17-18
Data management	19	Plans for data entry, coding, security, and storage, including any related processes to promote data quality (eg, double data entry; range checks for data values). Reference to where details of data management procedures can be found, if not in the protocol	23-24
Statistical methods	20a	Statistical methods for analysing primary and secondary outcomes. Reference to where other details of the statistical analysis plan can be found, if not in the protocol	17-18
	20b	Methods for any additional analyses (eg, subgroup and adjusted analyses)	17-18
	20c	Definition of analysis population relating to protocol non-adherence (eg, as randomised analysis), and any statistical methods to handle missing data (eg, multiple imputation)	-
**Methods: Monitoring**			
Data monitoring	21a	Composition of data monitoring committee (DMC); summary of its role and reporting structure; statement of whether it is independent from the sponsor and competing interests; and reference to where further details about its charter can be found, if not in the protocol. Alternatively, an explanation of why a DMC is not needed	23
	21b	Description of any interim analyses and stopping guidelines, including who will have access to these interim results and make the final decision to terminate the trial	-
Harms	22	Plans for collecting, assessing, reporting, and managing solicited and spontaneously reported adverse events and other unintended effects of trial interventions or trial conduct	20-22

For peer review only - http://bmjopen.bmj.com/site/about/guidelines.xhtml

Auditing	23	Frequency and procedures for auditing trial conduct, if any, and whether the process will be independent from investigators and the sponsor	-
**Ethics and dissemination**			
Research ethics approval	24	Plans for seeking research ethics committee/institutional review board (REC/IRB) approval	22
Protocol amendments	25	Plans for communicating important protocol modifications (eg, changes to eligibility criteria, outcomes, analyses) to relevant parties (eg, investigators, REC/IRBs, trial participants, trial registries, journals, regulators)	22
Consent or assent	26a	Who will obtain informed consent or assent from potential trial participants or authorised surrogates, and how (see Item 32)	22-23
	26b	Additional consent provisions for collection and use of participant data and biological specimens in ancillary studies, if applicable	22-23
Confidentiality	27	How personal information about potential and enrolled participants will be collected, shared, and maintained in order to protect confidentiality before, during, and after the trial	23-24
Declaration of interests	28	Financial and other competing interests for principal investigators for the overall trial and each study site	25
Access to data	29	Statement of who will have access to the final trial dataset, and disclosure of contractual agreements that limit such access for investigators	23
Ancillary and post-trial care	30	Provisions, if any, for ancillary and post-trial care, and for compensation to those who suffer harm from trial participation	22
Dissemination policy	31a	Plans for investigators and sponsor to communicate trial results to participants, healthcare professionals, the public, and other relevant groups (eg, via publication, reporting in results databases, or other data sharing arrangements), including any publication restrictions	24
	31b	Authorship eligibility guidelines and any intended use of professional writers	24
	31c	Plans, if any, for granting public access to the full protocol, participant-level dataset, and statistical code	-

For peer review

**Appendices**			
Informed consent materials	32	Model consent form and other related documentation given to participants and authorised surrogates	Appendix I, II and III
Biological specimens	33	Plans for collection, laboratory evaluation, and storage of biological specimens for genetic or molecular analysis in the current trial and for future use in ancillary studies, if applicable	23-24

*It is strongly recommended that this checklist be read in conjunction with the SPIRIT 2013 Explanation & Elaboration for important clarification on the items. Amendments to the protocol should be tracked and dated. The SPIRIT checklist is copyrighted by the SPIRIT Group under the Creative Commons "Attribution-NonCommercial-NoDerivs 3.0 Unported" license.

For peer review only

Appendix I – Consent Form for Screening: All Participants

Informed Consent Form for Screening
REC No. 22/EE/0010, Date: 08.02.2022, Version: 1.2

Psychosis Immune Mechanism Stratified Medicine Trial: The PIMS Trial

PARTICIPANT ID: ________

Thank you for considering taking part in the PIMS Trial. The research team must explain the eligibility assessment of the study to you before you agree to take part. If you have any questions arising from the Participant Information Sheet or from the explanation already given to you, please ask a member of the research team before you decide to participate. You will be given a copy of this Informed Consent Form to keep for future reference.

Please read the statements below and insert your initial in the box next to each statement if you agree with them:

Statement	Initial Here	
1. I have read and understood the information sheet version XX, DD.MM.YYYY and have had the opportunity to ask questions.		
2. I understand that my participation is voluntary and I am free to withdraw at any time without giving a reason, without my current or future medical care or legal rights being affected.		
3. I agree to provide blood samples for eligibility screening. I understand my blood samples will be analysed to test for evidence of immune activation. The purposes and possible risks of donating these samples have been explained to me. I understand that donated samples will be considered a gift but I will have the right to withdraw permission for analysis.		
4. I agree that my GP can be told that I am participating in the eligibility assessment of the PIMS Trial, and can be informed if any unexpected results are found pertaining specifically to my health.		
5. I consent for my GP/Psychiatrist to share information from my medical record in order to confirm my eligibility to take part in this study. The study team may access my GP/Psychiatrist records if necessary.		
Optional (Not agreeing to these will not exclude you from this study). Please tick Yes / No (as appropriate)	Yes	No
6. I agree that the samples can be stored after completion of the screening analysis, for use in future, ethically approved, non-genetic studies, even if I am deemed non-eligible to partake in the PIMS study.		
7. I agree that data and samples can be stored after completion of the PIMS Trial for use in future, ethically approved, genetic studies. This includes the main stocks of any genetic material collected, such as DNA and RNA.		

If you want to participate in the screening session of the PIMS Trial, please sign your name below:

Participant Signature ______________________

Participant Full Name _____________________

For peer review only - http://bmjopen.bmj.com/site/about/guidelines.xhtml

Date ____/ ____/ _______

The researcher who has explained this study to you also needs to sign this form:

Staff Signature ______________________

Staff Full Name _____________________

Date ____/ ____/ _______

Thank you for your help.

By completing and returning this form, you are giving us your consent that the personal information you provide will be treated as strictly confidential and handled in accordance with the provisions of the UK Data Protection Act 2018.

**When completed: 1 for participant; and 1 for researcher site file.*

For peer review only

Appendix II – Informed Consent Form for Study Participation: Patients

Informed Consent Form for Study Participation

REC No. 22/EE/0010, Date: 24.08.2022, Version: 1.5

Psychosis Immune Mechanism Stratified Medicine Trial: The PIMS Trial

PARTICIPANT ID: ________

Thank you for taking part in the PIMS Trial eligibility assessment. Based on this assessment, you are eligible to take part in the PIMS Trial. Before you agree to take part, the research team must explain the study to you. If you have any questions arising from the Participant Information Sheet or from the explanation already given to you, please ask a member of the research team before you decide to participate. You will be given a copy of this Informed Consent Form to keep for future reference.

Please read the statements below and insert your initial in the box next to each statement if you agree with them:

Statement	Initial Here
1. I have read and understood the information sheet version XX dated DD.MM.YYYY and have had the opportunity to ask questions.	
2. I agree to take part in the PIMS Trial. I understand that my participation is voluntary and I am free to withdraw at any time without giving a reason, without my current or future medical care or legal rights being affected.	
3. I understand that confidentiality and anonymity will be maintained and it will not be possible to identify me in any publications.	
4. I agree to partake in interviews, complete questionnaires, and cognitive tests as part of this study. I understand what will happen during the study assessments.	
5. I agree to provide blood samples. The purposes and possible risks of donating these samples have been explained to me. I understand that donated samples will be considered a gift but I will have the right to withdraw permission for analysis.	
6. I understand that blood samples collected from me will be used to measure non-genetic factors such as biochemical changes in the blood.	
7. I agree that the samples and information I provide can be stored, used and shared between PIMS Trial sites and with collaborators/contractors for the purpose of the study.	
8. I understand that blood samples collected will be stored at PIMS Trial centres.	
9. I understand that any of my samples (labelled with an anonymous ID only), or any information obtained from them, including the sequence of my genetic material, may be sent to specialist research laboratories in the UK and abroad for analyses and the results returned to PIMS Trial centres. Researchers at these laboratories have no access to personal information about study participants.	
10. I agree, if necessary, to provide blood/urine samples to test for pregnancy, COVID-19 immunity, Hepatitis B, Hepatitis C, HIV, VZV and Tuberculosis, and to undergo a chest X ray.	
11. I agree to being randomised into the tocilizumab or placebo group if deemed eligible to take part.	

For peer review only

12. I understand that taking part will involve the administration of a single intravenous infusion of the anti-inflammatory drug tocilizumab or normal saline.		
13. I agree that my GP can be told that I am participating in this study, and about any findings that require further attention.		
14. I understand that information related to my participation in this study may be accessed by responsible individuals from the sponsors of this study for quality control purposes. I give permission for these individuals to have access to this data.		
15. I understand that relevant sections of my medical notes and data collected during the study, may be looked at by individuals from research team, from regulatory authorities or from the NHS Trust, where it is relevant to my taking part in this research. I give permission for these individuals to have access to my records.		
Optional (Not agreeing to these will not exclude you from this study). Please tick Yes / No (as appropriate)	Yes	No
16. I agree to undergo brain scans as part of the PIMS Trial.		
17. I agree to be contacted in future by researchers to participate in follow up studies to this project, or in future studies of a similar nature.		
18. I understand that researchers may use the blood samples for genetic analysis.		
19. I agree that the samples can be stored after completion of the PIMS Trial for use in future, ethically approved, non-genetic studies.		
20. I agree that the information I give can be stored after completion of the PIMS Trial for use in future, ethically approved, non-genetic studies.		
21. I agree that data and samples can be stored after completion of the PIMS Trial for use in future, ethically approved, genetic studies. This includes the main stocks of any genetic material collected, such as DNA and RNA.		

If you want to participate in the PIMS Trial, please sign your name below:

Participant Signature _______________________

Participant Full Name ______________________

Date ____/ ____/ _______

Research staff who has explained this study to you also needs to sign this form:

Staff Signature _______________________

Staff Full Name ______________________

Date ____/ ____/ _______

For peer review only

For peer review only - http://bmjopen.bmj.com/site/about/guidelines.xhtml

Thank you for your help.

By completing and returning this form, you are giving us your consent that the personal information you provide will be treated as strictly confidential and handled in accordance with the provisions of the UK Data Protection Act 2018.

***When completed: 1 for participant; and 1 for researcher site file.*

For peer review only

Appendix III – Informed Consent Form for Study Participation: Healthy Controls

Healthy Controls Informed Consent Form for Study Participation
REC No. 22/EE/0010, Date: 17.02.2022, Version: 1

Psychosis Immune Mechanism Stratified Medicine Trial: The PIMS Trial

PARTICIPANT ID: ________

Thank you for taking part in the PIMS Trial eligibility assessment. Based on this assessment, you are eligible to take part in the PIMS Trial. Before you agree to take part, the research team must explain the study to you. If you have any questions arising from the Participant Information Sheet or from the explanation already given to you, please ask a member of the research team before you decide to participate. You will be given a copy of this Informed Consent Form to keep for future reference.

Please read the statements below and insert your initial in the box next to each statement if you agree with them:

Statement	Initial Here	
1. I have read and understood the information sheet version XX dated DD.MM.YYYY and have had the opportunity to ask questions.		
2. I agree to take part in the PIMS Trial. I understand that my participation is voluntary, and I am free to withdraw at any time without giving a reason, without my current or future medical care or legal rights being affected.		
3. I understand that confidentiality and anonymity will be maintained, and it will not be possible to identify me in any publications.		
4. I agree to partake in interviews, complete questionnaires, and cognitive tests as part of this study. I understand what will happen during the study assessments.		
5. I agree to provide blood samples. The purposes and possible risks of donating these samples have been explained to me. I understand that donated samples will be considered a gift, but I will have the right to withdraw permission for analysis.		
6. I understand that my blood samples collected will be stored at PIMS Trial centres.		
7. I understand that blood samples collected from me will be used to measure non-genetic factors such as biochemical changes in the blood.		
8. I understand that any of my samples (labelled with an anonymous ID only), or any information obtained from them, including the sequence of my genetic material, may be sent to specialist research laboratories in the UK and abroad for analyses and the results returned to PIMS Trial centres. Researchers at these laboratories have no access to personal information about study participants.		
9. I agree that the samples and information I provide can be stored, used and shared between PIMS Trial sites and with collaborators/contractors for the purpose of the study.		
10. I understand that information related to my participation in this study may be accessed by responsible individuals from the sponsors of this study for quality control purposes. I give permission for these individuals to have access to this data.		
Optional (Not agreeing to these will not exclude you from this study). Please tick Yes / No (as appropriate)	Yes	No

For peer review only - http://bmjopen.bmj.com/site/about/guidelines.xhtml

11. I agree to undergo brain scans as part of the PIMS Trial.		
12. I agree to be contacted in future by researchers to participate in follow up studies to this project, or in future studies of a similar nature.		
13. I understand that researchers may use the blood samples for genetic analysis.		
14. I agree that the samples can be stored after completion of the PIMS Trial for use in future, ethically approved, non-genetic studies.		
15. I agree that the information I give can be stored after completion of the PIMS Trial for use in future, ethically approved, non-genetic studies.		
16. I agree that data and samples can be stored after completion of the PIMS Trial for use in future, ethically approved, genetic studies. This includes the main stocks of any genetic material collected, such as DNA and RNA.		

If you want to participate in the PIMS Trial, please sign your name below:

Participant Signature ______________________

Participant Full Name _____________________

Date ____/ ____/ _______

Research staff who has explained this study to you also needs to sign this form:

Staff Signature ______________________

Staff Full Name _____________________

Date ____/ ____/ _______

Thank you for your help.

By completing and returning this form, you are giving us your consent that the personal information you provide will be treated as strictly confidential and handled in accordance with the provisions of the UK Data Protection Act 2018.

**When completed: 1 for participant; and 1 for researcher site file.*

For peer review only
